# New Cysteine Protease Inhibitors: Electrophilic (Het)arenes and Unexpected Prodrug Identification for the *Trypanosoma* Protease Rhodesain

**DOI:** 10.3390/molecules25061451

**Published:** 2020-03-23

**Authors:** Philipp Klein, Patrick Johe, Annika Wagner, Sascha Jung, Jonas Kühlborn, Fabian Barthels, Stefan Tenzer, Ute Distler, Waldemar Waigel, Bernd Engels, Ute A. Hellmich, Till Opatz, Tanja Schirmeister

**Affiliations:** 1 Department of Chemistry, Organic Chemistry Section, Johannes Gutenberg-Universität, 55128 Mainz, Germany; klein@uni-mainz.de (P.K.); jokuehlb@uni-mainz.de (J.K.); 2 Institute of Pharmaceutical and Biomedical Sciences, Johannes Gutenberg-Universität, 55128 Main, Germany; pajohe@uni-mainz.de (P.J.); sascha.jung@tu-dortmund.de (S.J.);; 3 Department of Chemistry, Biochemistry Section, Johannes Gutenberg-Universität, 55128 Mainz, Germany; a.wagner@uni-mainz.de (A.W.); u.hellmich@uni-mainz.de (U.A.H.); 4 Present address: Faculty of Chemistry and Chemical Biology, TU Dortmund University, 44227 Dortmund, Germany; 5 Institute of Immunology, University Medical Center, Johannes Gutenberg-Universität Mainz, 55131 Mainz, Germany; tenzer@uni-mainz.de (S.T.); ute.distler@uni-mainz.de (U.D.); 6 Institute of Physical and Theoretical Chemistry, Universität Würzburg, 97074 Würzburg, Germany; waldemar.waigel@uni-wuerzburg.de (W.W.); bernd.engels@uni-wuerzburg.de (B.E.); 7 Centre for Biomolecular Magnetic Resonance (BMRZ), Goethe-University Frankfurt, 60323 Frankfurt, Germany

**Keywords:** cysteine protease, rhodesain, electrophilic (het)arene, nucleophilic aromatic substitution, Meisenheimer complex, π-complex, prodrug

## Abstract

Electrophilic (het)arenes can undergo reactions with nucleophiles yielding π- or Meisenheimer (σ-) complexes or the products of the S_N_Ar addition/elimination reactions. Such building blocks have only rarely been employed for the design of enzyme inhibitors. Herein, we demonstrate the combination of a peptidic recognition sequence with such electrophilic (het)arenes to generate highly active inhibitors of disease-relevant proteases. We further elucidate an unexpected mode of action for the trypanosomal protease rhodesain using NMR spectroscopy and mass spectrometry, enzyme kinetics and various types of simulations. After hydrolysis of an ester function in the recognition sequence of a weakly active prodrug inhibitor, the liberated carboxylic acid represents a highly potent inhibitor of rhodesain (*K*_i_ = 4.0 nM). The simulations indicate that, after the cleavage of the ester, the carboxylic acid leaves the active site and re-binds to the enzyme in an orientation that allows the formation of a very stable π-complex between the catalytic dyad (Cys-25/His-162) of rhodesain and the electrophilic aromatic moiety. The reversible inhibition mode results because the S_N_Ar reaction, which is found in an alkaline solvent containing a low molecular weight thiol, is hindered within the enzyme due to the presence of the positively charged imidazolium ring of His-162. Comparisons between measured and calculated NMR shifts support this interpretation.

## 1. Introduction

Inhibition of enzymes may occur either reversibly or irreversibly. In the case of irreversible inhibition, a covalent reaction between a nucleophilic amino acid (in most cases, an activated Cys, Ser or Thr side chain), either of the active or an allosteric site, reacts with an electrophilic functional group of the inhibitor, the so-called “warhead”. Besides this reactive building block, enzyme inhibitors also contain a recognition unit fitting into the substrate binding pockets. In the case of protease inhibitors these are mainly peptidic or peptidomimetic sequences. However, not all covalent reactions of enzyme and inhibitor lead to irreversible inhibition, and this has received increasing attention in drug development in the last years to design covalent, but reversible inhibitors due to several advantages over non-covalent inhibitors such as longer target residence times or higher potency and ligand efficiency (for reviews on this topic see [1,2,3,4,5,6,7,8]).

In contrast to classical electrophilic building blocks like Michael-acceptor systems [9,10,11,12,13,14,15,16,17], nitriles [18,19,20,21], aldehydes, ketones [22,23,24], three-membered heterocycles [25,26,27,28] or a iodoacetic acid moiety [29,30], (hetero)aromatic electrophiles, which can react via nucleophilic addition (yielding π- or σ-complexes) or substitution reactions (S_N_Ar) have only rarely been employed [31,32,33,34,35]. Such aromatic moieties have also seldom been investigated as non-covalently binding parts of the recognition units of enzyme inhibitors [36,37].

We therefore aimed to explore the potential of such groups as new building blocks for cysteine proteases of the papain family, specifically the human cathepsins B and L, and the *Trypanosoma* protease rhodesain. The human enzymes of this family play crucial roles in tumor diseases [38,39]. The cathepsin L-like protease rhodesain from *Trypanosoma brucei rhodesiense*, the parasite causing human African trypanosomiasis (HAT), is essential for the parasite’s survival, since it is involved in several pathological processes in the host. These include, e.g., the crossing of the parasite through the blood–brain barrier as well as the turnover of variant surface glycoproteins (VSGs), and degradation of host immunoglobulins [40,41].

In the current proof-of-principle study, we tested the overall potency of (hetero)aromatics as building blocks for peptidic protease inhibitors. Since we were interested in the inhibition properties of the (hetero)aromatic moiety, we used the same recognition unit, the dipeptide sequence H_2_N-l-Phe-l-Leu-OBn, and attached various (hetero)aromatic electrophiles to its N-terminus. This dipeptide was chosen for two reasons: (1) It was previously shown to be an appropriate recognition unit for cathepsin L-like cysteine proteases supposed to bind into the substrate-binding pockets in an anti-substrate orientation [14,15,42]; (2) In contrast to amino acids with functionalized side chains, no additional protection/deprotection steps are necessary during synthesis.

For one inhibitor, we observed that the benzyl ester of the recognition unit was hydrolyzed by the target proteases. Intriguingly, the resulting free acid (with HN-l-Phe-l-Leu-OH moiety) represents a nanomolar inhibitor against rhodesain.

The inhibition potencies of the new inhibitors were tested on the human cathepsins B and L (cath. B, cath. L), the cathepsin L-like protease rhodesain (rhod.) from *T. b. rhodesiense*, and, in order to check their selectivity and to exclude promiscuous inhibition, on the *Staphylococcus aureus* cysteine protease and transpeptidase sortase A with a “reversely protonated” catalytic cysteine residue [43], as well as on the serine protease of the Dengue virus (DENV PR), which shares the P1 specificity for Arg with cathepsins.

The formation of a complex between inhibitor and the target protease rhodesain was indicated by mass spectrometry and NMR spectroscopy. Possible binding modes of selected inhibitors were analyzed by docking, followed by subsequent molecular dynamics (MD) simulations. Additionally, we performed quantum mechanical (QM) computations for model systems and quantum mechanics / molecular mechanics (QM/MM) simulations, which include the enzyme environment.

## 2. Results

### 2.1. Syntheses

**Scheme 1:**
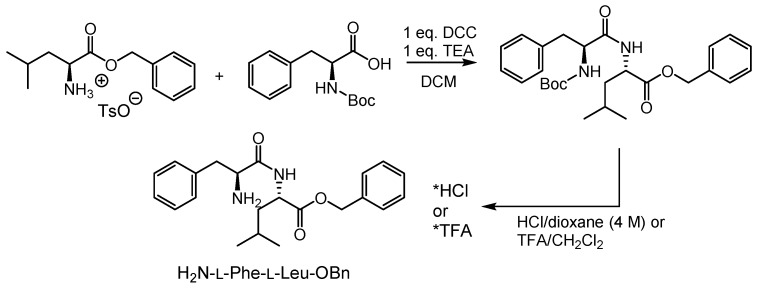
Synthesis of the dipeptide sequence of new protease inhibitors.

The synthesis of the new inhibitors started from commercially available l-leucine benzyl ester *p*-toluenesulfonate and Boc-protected l-phenylalanine, which were coupled by carbodiimide chemistry. The dipeptide product was easily purified by recrystallization (Scheme 1). Deprotection with either hydrochloric acid in dioxane (4 M) or trifluoro-acetic acid (TFA) in dichloromethane (DCM)(40%) afforded the ammonium salt, which was directly used in the subsequent steps.

**Scheme 2:**
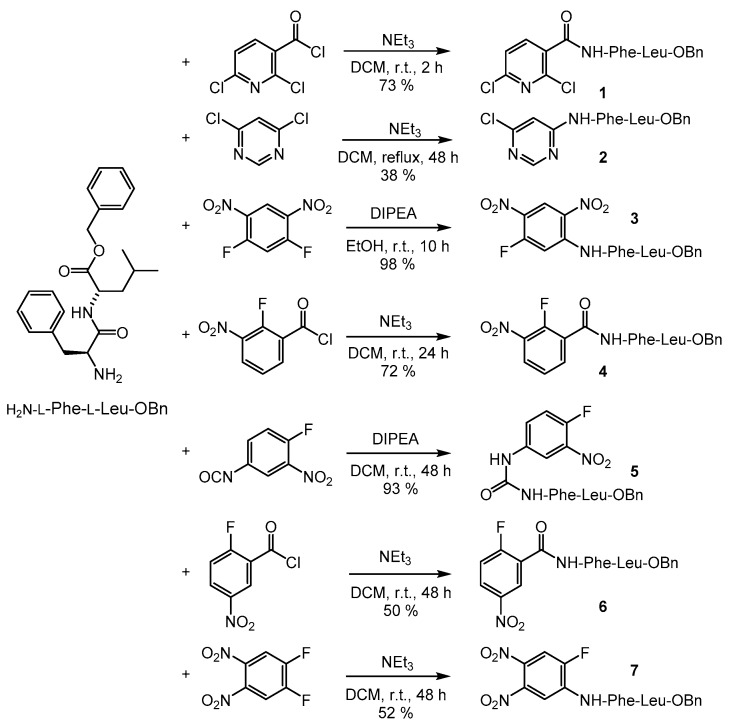
Syntheses of potential protease inhibitors of the general structure R-NH-l-Phe-l-Leu-OBn with (hetero)aromatic units (R).

The potential inhibitors were synthesized either by S*_N_*Ar reaction on a (hetero)aromatic unit carrying two potential leaving groups (compounds **2**, **3**, and **7**) or by reaction with an electrophilic group attached to the ring system (e.g., acid chloride, isocyanate and compounds **1**, **4**, **5**, and **6**, Scheme 2).

**Scheme 3:**
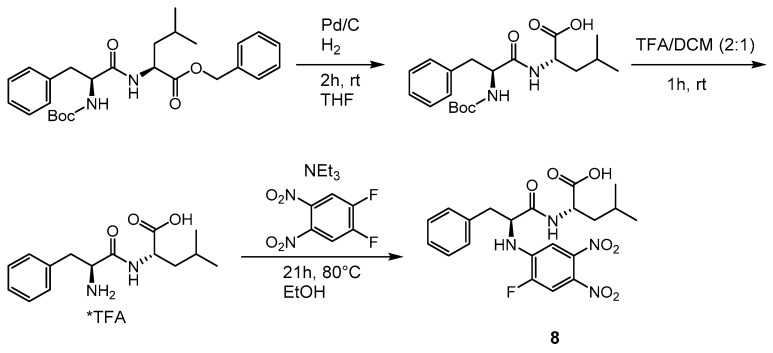
Synthesis of acid **8** (R-NH-l-Phe-l-Leu-OH).

To further investigate whether the hydrolysis product of compound **7** is the active compound as indicated by MS, NMR and hydrolysis studies (see Section 2.3, Section 2.4 and Section 2.5), the corresponding free acid **8** was also synthesized (Scheme 3). First attempts to cleave the benzyl ester of compound **7** with PPL (porcine pancreatic lipase) were unsuccessful. The compound was synthesized starting from the fully protected dipeptide Boc-NH-l-Phe-l-Leu-OBn. In a first step, the benzyl ester was removed by hydrogenolysis. A short filtration over celite yielded a crude material that was subjected to acidolytic removal of the Boc-protecting group with trifluoroacetic acid (TFA). The solvent was removed to perform the reaction with the warhead (1,2-difluoro-4,5-dinitrobenzene) in ethanol in the presence of triethylamine. After 21 h at 80 °C, the desired acid **8** was obtained after purification by preparative high-performance liquid chromatography (HPLC).

### 2.2. Enzyme Assays

**Figure 1:**
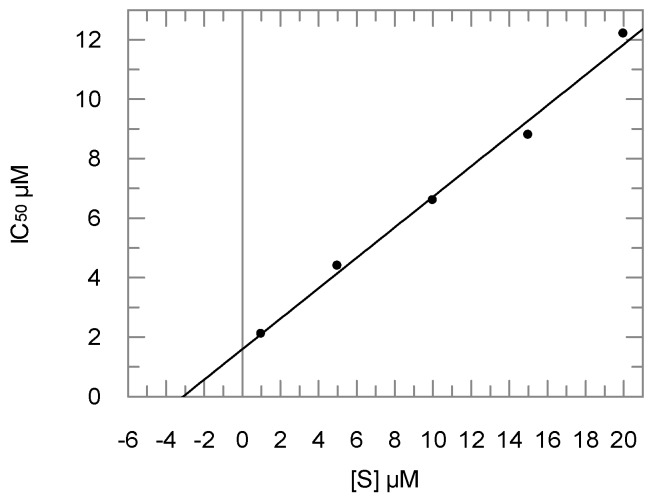
Dependence of inhibition potency (IC_50_ values) on substrate concentration for inhibition of cathepsin L by compound **7**. Increasing IC_50_ values at increasing substrate concentrations indicate a competitive inhibition mode.

Inhibition of cathepsins and rhodesain was tested with the fluorogenic substrate Cbz-Phe-Arg-AMC as described previously [19]. First, a screening at 20 µM inhibitor concentration was performed. For compounds showing >60% inhibition at this concentration, the IC_50_ values were determined. For the most active compounds, namely **4** and **7**, the IC_50_ values were determined at different substrate concentrations in order to check for competitive or non-competitive inhibition [44,45]. IC_50_ values were found to increase linearly with increasing substrate concentrations in both cases, indicating competitive inhibition (see exemplarily Figure 1 for compound **7**). The respective *K*_i_ values (Table 1) were determined from plots of the IC_50_ values against substrate concentration according to the Cheng–Prusoff relationship [46].

The progress curves for inhibition of cathepsins and rhodesain by the compounds were found to be linear in all cases, i.e., no time-dependent inhibition was observed, pointing to fast reversible inhibition.

The highest inhibition of cathepsin L was found with the benzoic acid amide **4** (*K*_i_ = 0.66 µM) and the amine **7** (*K*_i_ = 1.6 µM)**.** The latter also turned out to be the best inhibitor of cathepsin B and the Dengue virus (DENV) protease [47,48], however with lower inhibitory potency (Cath. B: *K*_i_ = 4.4 µM; DENV PR: 31% at 20 µM). These two compounds were also tested for inhibition of the cathepsin L-like parasitic protease rhodesain. While only a weak inhibitory potency (45% inhibition at 20 µM) was detected for compound **7**, the amide **4** displayed a *K*_i_ value of 0.29 µM. 

**Figure 2:**
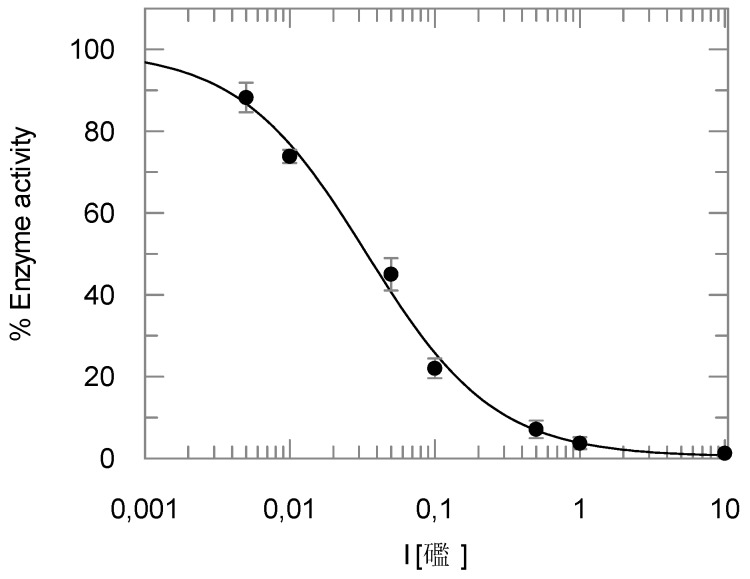
Inhibition of rhodesain by compound **8**.

The benzyl ester **7** was found to be hydrolyzed, yielding the free acid **8** (see Section 2.3, Section 2.4 and Section 2.5). Thus, the enzyme assays were also performed with the acid **8**. *K*_i_ values of 3.0 ± 0.9 µM for cathepsin L (*n* = 10), 1.50 ± 0.29 µM for cathepsin B (*n* = 6) and, most interestingly, 4.0 ± 1.3 nM (*n* = 14) for rhodesain, were determined (Figure 2). This indicates that acid **8** and ester **7** are similarly active on cathepsins L and B, whereas in the case of rhodesain, an enormous increase in inhibition potency was observed comparing the benzyl ester **7** and the respective free acid **8**.

To investigate the inhibitory effects against a cysteine protease with a protonated thiol instead of an imidazolium/thiolate dyad, compounds **4**, **7** and **8** were tested for inhibition of the *S. aureus* sortase A. None of the inhibitors showed significant inhibition at a final inhibitor concentration of 20 µM (compound **4**: 11%; compound **7**: 16%; compound **8**: 0%), indicating selectivity for cysteine proteases from the CA-clan (cathepsins and rhodesain) over the CL-clan sortase A [49].

The progress curves for inhibition of cathepsins L and B and rhodesain by compound **8** did not clearly show time-dependent behavior. Since the inhibition mechanism (reversible vs. irreversible) can unequivocally not be deduced from the progress curves, we also used other methods to ascertain the (ir)reversibility of the inhibition: (A) The IC_50_ values for inhibition of rhodesain were determined in dependence of incubation time of enzyme and inhibitor prior to substrate addition (10 min vs. 45 min). No differences were observed indicating reversible inhibition; (B) Furthermore, dilution assays were performed with rhodesain. To this end, the enzyme was completely inhibited by an inhibitor concentration 100 fold higher than the IC_50_ value. After dilution of the enzyme-inhibitor mixture with a buffer by a factor of 100, slow and time-dependent reactivation of the enzyme was observed, finally proving the reversibility of inhibition (see Supplementary Material).

### 2.3. ^19^F NMR Spectroscopy

**Figure 3:**
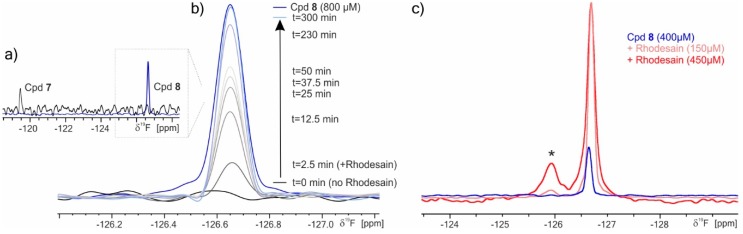
^19^F NMR spectra: (**a**) Ester **7** and acid **8** are readily distinguishable by their chemical shifts. (**b**) Time-resolved ^19^F NMR shows rhodesain-dependent turnover of **7** into **8**. As a control, a separate spectrum of the expected amount of compound **8** that should have formed from **7** in the NMR-based enzymatic assay is recorded (blue trace). Due to the poor solubility of compound **7** (compare (a)), no peak was observed within the 128 scans run for each time-trace. Upon addition of rhodesain, the turbid sample gradually turned clear and the resonance for compound **8** appeared. Therefore, the spectral region has been confined to that displaying the chemical shift for compound **8**. (**c**) Acid **8** forms a stable complex (asterisk) with rhodesain. The peak intensity depends on the amount of protein. For reference, the spectrum of free compound **8** (blue) is included (as shown in (**a**)).

The interaction of the fluorinated inhibitors **7** and **8** with rhodesain was probed via ^19^F NMR spectroscopy (Figure 3). Compound **7** displayed some solubility issues apparent from the observed turbidity of the sample and the reduced signal-to-noise ratio in the spectrum (Figure 3a). To probe the rhodesain-mediated turnover of **7** into **8**, we recorded time-resolved ^19^F NMR spectra of **7** (800 µM) in the presence of 4 µM rhodesain (Figure 3b). During the measurement, the solution became clear and a dramatic difference in the chemical shift occurred to values identical to those observed for compound **8**. The reaction was subsequently analyzed by MS (see Section 2.4), which confirmed the formation of the free acid **8**.

To observe the chemical shift of the rhodesain-bound reaction product, a higher concentration of rhodesain (150 or 450 µM) was incubated with **8**. Indeed, a new, broad peak (Figure 3c, asterisk) was observed, which probably results from an enzyme-inhibitor adduct (see Section 2.6 and Section 2.7).

### 2.4. Mass Spectrometry

**Figure 4:**
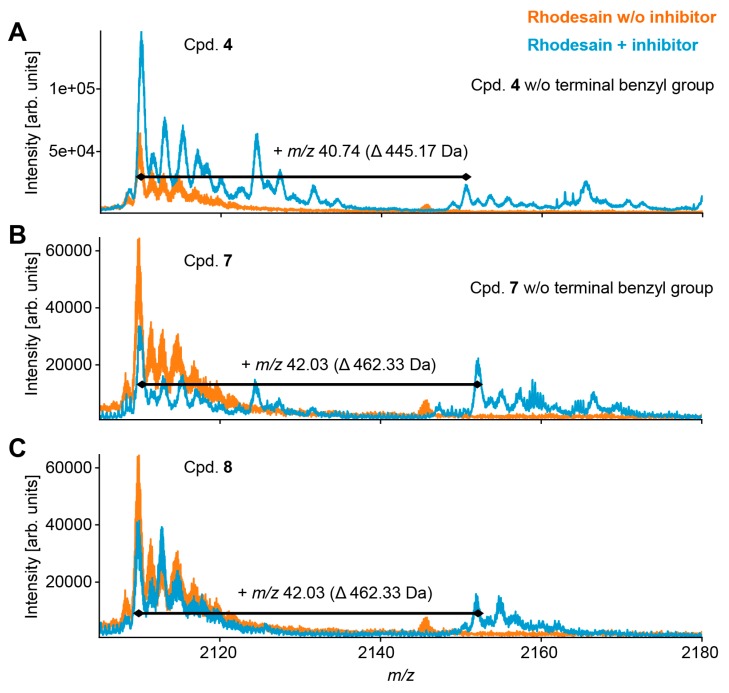
ESI-MS mass spectra of rhodesain ([M + 11 H]^11+^ at *m*/*z* 2109.9 +/– 20 ppm) in the absence (orange) or presence (blue) of compounds **4** (**A**), **7** (**B**) and **8** (**C**).

To further characterize the interaction between rhodesain and the different inhibitors, we performed a liquid chromatography-mass spectrometric (LC–MS coupled to nano-ESI-Q-TOF) analysis of rhodesain that had been incubated with compounds **4** or **7**. Samples were analyzed in positive ion mode on a quadrupole time-of-flight mass spectrometer (Synapt G2-S HDMS, Waters Corporation) using electrospray ionization (ESI). Rhodesain without inhibitor served as a control. Both compounds reacted with rhodesain (Figure 4). Interestingly, we found that rhodesain catalyzes the hydrolysis of the benzyl esters of the dipeptide recognition units to the corresponding acids, indicated by a mass shift of 90 Da corresponding to the loss of the terminal benzyl group (Figure 4). Notably, only reaction products of the hydrolyzed compounds with rhodesain were detectable by LC–MS analysis. Since the NMR experiments also showed conversion of ester **7** into the acid **8** catalyzed by rhodesain, the LC–MS analyses were repeated with the acid **8**. These experiments revealed the formation of an adduct with rhodesain for both acid **8** and ester **7** showing exactly the same pattern (Figure 4). These data are in line with the previous NMR spectroscopic observation that the benzyl ester of the dipeptide recognition unit is first hydrolyzed by rhodesain, which subsequently forms a very stable complex with the hydrolysis product. This may also hold true for inhibitor **4**. Since the complex formation is reversible—as shown by the enzyme assays discussed above—and the mass spectra show the mass of the adduct of enzyme and acid **8** and not the mass of an adduct resulting from nucleophilic substitution of either the fluoride or a nitro group, the S_N_Ar reaction cannot be completed. Hence, the data are only consistent with a π- or a Meisenheimer (σ-) complex.

### 2.5. Hydrolysis of Ester 7 by Rhodesain

**Figure 5:**
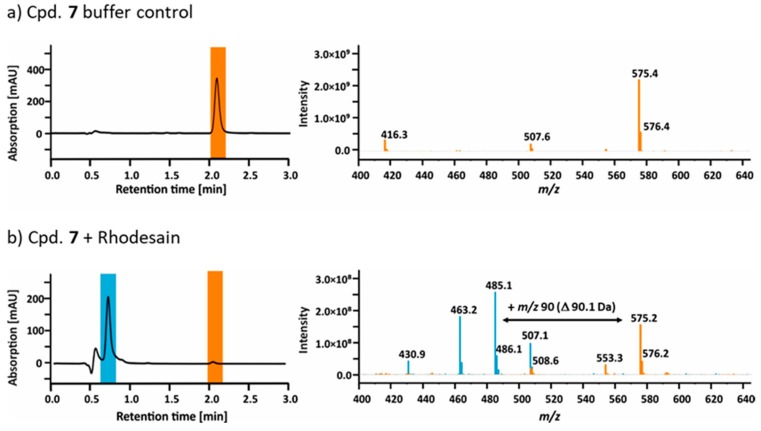
Chromatograms and mass spectra of the LC–MS runs of compound **7** preincubated with (**a**) buffer or (**b**) buffer and catalytically active rhodesain. The elution peaks and mass signals of the unconverted ester **7** are presented in orange, the signals of the resulting acid **8** are highlighted in blue.

In order to verify that the ester cleavage of compound **7** indeed occurs by enzymatic hydrolysis catalyzed by the target protease rhodesain, the compound was incubated with: (a) catalytically active rhodesain; (b) reaction buffer; or (c) rhodesain inactivated by the vinyl sulfone K11777 [50]. The reaction mixtures were subsequently analyzed by LC–MS. Unconverted ester **7** and acid **8** were eluted at t(**8**) = 0.9 min and t(**7**) = 2.1 min. The detected masses matched the theoretical values ([**7** + H^+^] = 553.3 m/z, [**7** + Na^+^] = 575.4 m/z, [**8** + H^+^] = 463.3 m/z and [**8** + Na^+^] = 485.3 m/z). A complete conversion of the ester **7** into acid **8** was observed in the case of catalytically active rhodesain (a) (Figure 5), but not with the irreversibly inactivated enzyme (c). A control showed that the ester was not hydrolyzed by the reaction buffer (b) (Figure 5).

### 2.6. Reaction of Inhibitor 7 with a Low Molecular Weight Thiol

**Scheme 4:**
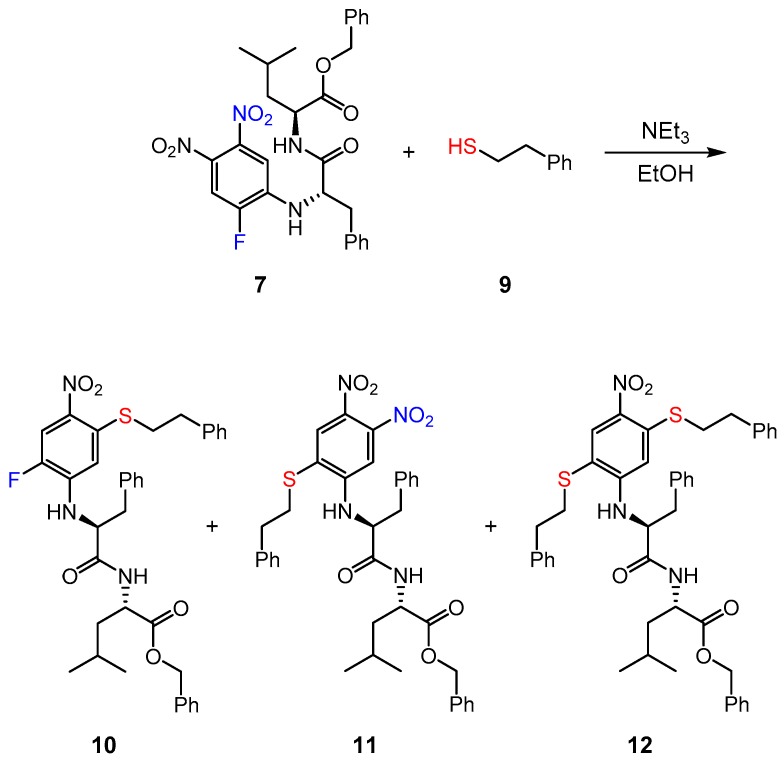
Reaction of compound **7** with 2-phenylethanethiol and observed products.

Since cathepsins and rhodesain are cysteine proteases, we also investigated the interaction between the inhibitor **7** and a low molecular weight thiol (LMW thiol). The reaction of compound **7** was carried out with 2-phenylethanethiol (**9**) in the presence of a base in ethanol (Scheme 4). All three possible reaction products resulting from substitution of the nitro- (**10**), fluoro- (**11**) and of both groups (**12**) were obtained in the experiments, in which the reaction temperature (rt or 80 °C), the amounts of thiol and base (excess or two equivalents each) and the reaction time (4 h, 16 h, 28 h) were varied. The main product (ca. 60–70%) detected in the 16 h reaction at 80 °C with a large excess of thiol and triethylamine was the double-substitution product (**12**) accompanied by small amounts of **10** and **11**. At rt with two eq. base and thiol, ca. 70–80% unreacted starting material, accompanied by small amounts of all three reaction products (**10**–**12**), was detected. After 28 h at rt (2 eq. base and thiol), the amount of starting material decreased (to ca. 40%) and the amounts of the three substitution products (**10**–**12**) increased (to ca. 15–25% each). These reactions show that the chosen electrophilic aromatic moiety is indeed competent to undergo nucleophilic aromatic substitutions. 

The regiochemistry of the substitution in product **12** could not be unequivocally determined by the usual 2D NMR methods, so that DFT calculations and a DP4-probability analysis were performed. The observed NMR chemical shifts were compared with those of values for both possible regioisomers computed by DFT calculations. Conformational analyses were performed on a PM6 (Parametric Method 6) [51] or MMFF (Merck Molecular Force Field) [52] level of theory and the geometries of all conformers found were subsequently reoptimized [B3LYP/6-31G(d)] [53,54,55,56,57,58,59]. For the calculation of the NMR shielding tensors, the *m*PW1PW91 [60] functional was used in combination with the 6-31 + G(d,p) [57,58,59] basis set and the gauge-independent atomic orbital (GIAO) method [61]. For this computation, undirected solvent effects were taken into account using the integral equation formalism polarizable continuum model (IEFPCM) [62]. After Boltzmann weighting, the DP4+ probabilities [63] (see Supplementary Material) were calculated, which unambiguously show that the most likely regioisomer is the one with both thioether moieties in *para*-position to each other (**12**).

**Figure 6:**
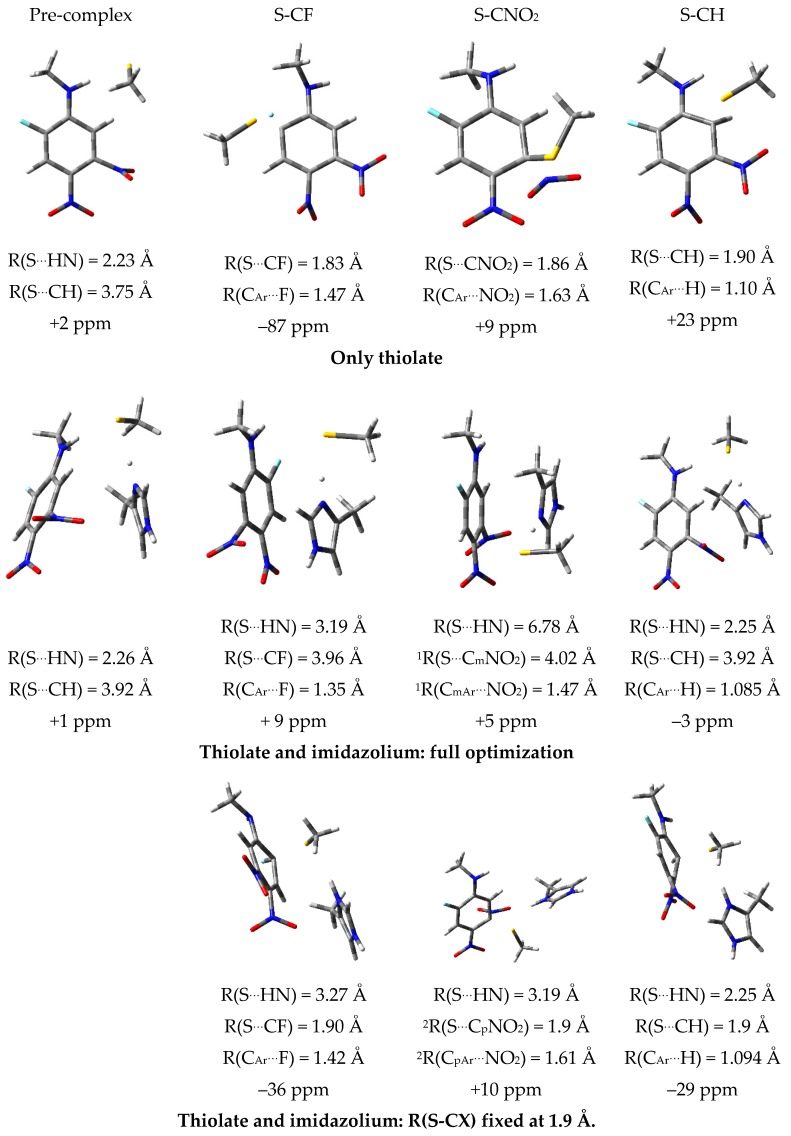
Computed structures and ^19^F shifts relative to the ^19^F shift of the free inhibitor for the different model systems. For the undistorted inhibitor the following distances are obtained: R(CAr^…^F) = 1.35 Å, R(CAr^…^NO_2_) = 1.47 Å and R(CAr^…^H) = 1.085 Å. For further explanation see main text; ^1^ with respect to the NO_2_ group in meta-position to F; ^2^ with respect to the NO_2_ group in *para*-position to F. MP2, second-order Møller–Plesset perturbation theory.

High level quantum chemical calculations for appropriate model systems are often very helpful to understand the situation in an enzyme [28,64,65,66]. Hence, to shed some light on possible reaction intermediates, we also computed the relative stabilities of the involved Meisenheimer complexes in a polar solvent. To mimic the polar environment, we used the IEFPCM approach for a water environment [62]. To obtain information about the influence of the employed theoretical approach, we used density functional (ωB97XD [67]/6-31 + G* [59]) as well as the more reliable 2nd order perturbation theory without and with the SCS (spin-component-scaled) [68] approximation. For these computations we also employed the 6-31 + G* basis set. All computations were performed with the GAUSSIAN program package [69]. The data are summarized in Table 2. The computed structures are depicted in Figure 6. In all model systems, the inhibitor was approximated by the substituted benzene ring. 

The model system without an imidazole ring mimics the situation of the reaction with 2-phenylethanethiol (**9**) in the presence of a base in ethanol, in which the reaction can be viewed as a simple thiolate attack at the substituted ring. The corresponding complexes are depicted in Figure 6 in the row “only thiolate”. They were obtained by a full geometry optimization starting with a S^…^CX distance (X = F, NO_2_, H) of 1.9 Å. The resulting complexes represent Meisenheimer complexes as can be deduced from the distances: the distances R(S^…^CX) remain at about 1.9 Å and the distances R(C_Ar_^…^X) are already considerably elongated. According to the more accurate MP2 or SCS-MP2 computations, the Meisenheimer complexes represent stable minima on the hypersurface, which lie about 4–13 kcal/mol lower than the reactants. The computations also indicate a stable pre-complex stabilized by a hydrogen bond between the NH group and the attacking thiolate, which according to the more accurate MP2- and SCS-MP2-calculations, is similarly stable as the Meisenheimer complexes. It should be noted that wB97XD considerably underestimates the stability of the Meisenheimer complexes. The computations are in good agreement with the experiment, which finds S_N_Ar reactions between 2-phenylethanethiol (**9**) in the presence of a base in ethanol at rt. Figure 6 also contains the ^19^F NMR shifts relative to the value computed for the inhibitor. The computed variation upon the formation of the Meisenheimer complexes are magnitudes larger than the variation found experimentally for a possible inhibitor-enzyme complex (≈ 1 ppm).

Possible reactions between the substituted aromatic ring and the catalytic dyad of the enzyme differ from the solvent reaction in several ways. Besides steric restrictions, the thiolate group of the active-site cysteine residue is always stabilized by a strong salt bridge to the positively charged imidazolium ring of the active-site histidine residue, which might reduce the thiolate reactivity. To mimic this situation, we added a positively charged imidazolium ring to the model system. In the first calculation, we put the thiolate at a distance of about 1.9 Å from the attacked carbon center of the aromatic ring and performed a full geometry optimization. The resulting complexes are depicted in Figure 6 as “thiolate and imidazolium: full optimization”. The more reliable MP2- or SCS-MP2-computations predict that the resulting complexes are stable with respect to the fragmentation into the inhibitor and thiolate/imidazolium complex. The computed complexes, however, are not Meisenheimer complexes as found for the situation without the imidazolium (only thiolate). The R(S^…^CX) distances are about 3.9–4.0 Å and the R(C_Ar_^…^X) are not elongated. Nevertheless, the stability of this complex upon fragmentation into inhibitor and thiolate/imidazolium complex is about 20 kcal/mol, i.e., they are even more stable than the Meisenheimer complexes found for the “only thiolate” situation. It should be noted that the situation found for the attack at C−NO_2_ does not reflect the situation in the enzyme, because the thiolate and the imidazolium ring interchange their positions during the full optimization to maximize the interaction energy. The same holds true for the attack at C−F due to the orientation of the substituted ring. Both the pre-complex and the situation found for the attack at the CH group of the ring could also take place in the enzyme (see Section 2.7). To get more insights, we also built a complex in which we fixed the S–CX bond lengths to about 1.9 Å, i.e., we enforced a kind of Meisenheimer complex in which the positively charged imidazolium is present. According to MP2 or SCS-MP2, the resulting complexes (Figure 6: thiolate and imidazolium: R(S−CX fixed at 1.9 Å)) are 4–10 kcal/mol less stable than the complex obtained by the full optimization. This indicates that a thiolate/imidazolium complex should not be able to form a Meisenheimer complex. Again, the ^19^F NMR shifts were computed. Their variations with respect to the ^19^F NMR shift of the pure inhibitor are also given in Figure 6. It can be seen that the NMR shift variations found for the complexes are considerably smaller than those found for the corresponding Meisenheimer complexes. Furthermore, they are of similar size as the experimental shift difference. The comparison between the employed theoretical approaches clearly shows that the DFT functional ωB97XD underestimates the stability of the complexes by up to 10 kcal/mol.

Our model calculations indicate that the catalytic dyad of the enzyme, mimicked by a thiolate/imidazolium complex, behaves differently than a single attacking thiolate. Consequently, the formation of a Meisenheimer complex would not be expected for the enzyme, but instead, the formation of a quite stable complex with larger distances between the catalytic dyad and the substituted aromatic ring would be expected. From the orientation of the fragments, it resembles a π-complex. Despite this relatively long distance, the complex is quite stable. The magnitudes of the computed NMR shifts also indicate that a π- instead of a Meisenheimer complex is formed.

### 2.7. Theoretical Investigations of the Enzyme-Inhibitor Complex

Docking studies using the crystal structure of rhodesain (pdb 2p7u) were performed with acid **8** and its benzyl ester **7** to understand the molecular basis of the observed enzyme inhibition by acid **8** and of the observed enzymatic hydrolysis of ester **7** in more detail. Docking was performed with the FlexX/LeadIT 2.3.2 software suite [70]. The detailed procedure is given in the Supplementary Material. 

**Figure 7:**
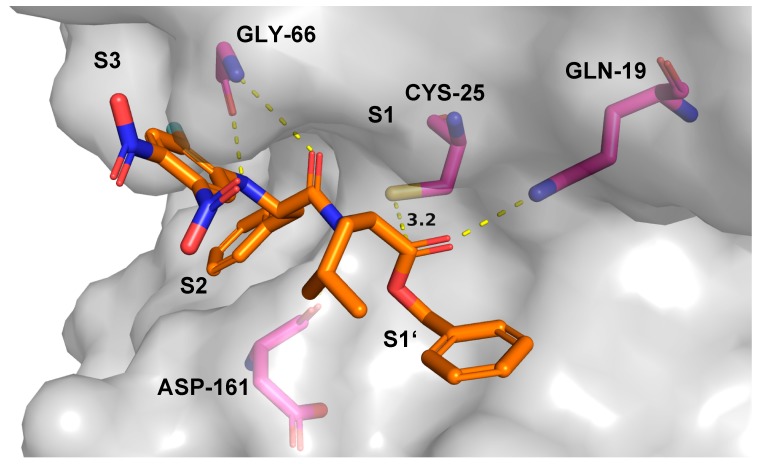
Substrate-like binding mode of ester **7**. Light grey: solvent accessible surface of rhodesain; magenta: carbon atoms of rhodesain amino acid residues; orange: carbon atoms of ester **7**; blue: nitrogen; red: oxygen; yellow: sulfur; cyan: fluorine.

For the benzyl ester **7**, three different kinds of poses were found. In the highest ranked pose (i.e., the pose with the most negative score of –20.56), the electrophilic aromatic ring binds to the S1′ pocket and is not close to the cysteine (see Figure S3 in the Supplementary Material for more details). In various other highly stable poses, this inhibitor moiety sits in the active site and hence is close to the active site cysteine (score –18.26, see Figure S4 in the Supplementary Material for more details). Additionally, multiple poses with a substrate-like orientation of compound **7** were predicted with the benzyl ester being in a position close to the cysteine (distance 3.2 Å, score –13.77, Figure 7). In this orientation, the ester carbonyl carbon atom is in a suitable position for nucleophilic attack initiating the hydrolysis of the ester. 

In this reverse, i.e., substrate-like orientation, the carbonyl oxygen of the ester group points towards Gln-19. The benzyl group extends into the S1′ pocket and the leucine residue is located in the S1 pocket. The phenylalanine side chain occupies the S2 pocket, and the aromatic ring is placed in the S3 pocket.

To investigate the stability of the poses in more detail, three MD simulations for each pose were performed. The poses in which the electrophilic aromatic ring sits in the active site were found to be quite stable, although in some cases parts of the inhibitor left the enzyme. However, the enzyme-inhibitor complex never split up completely. Furthermore, the distances between the cysteine moiety and the aromatic ring remained at distances above 4.5 Å, i.e., for these poses, an attack at the substituted aromatic ring is unlikely.

**Figure 8:**
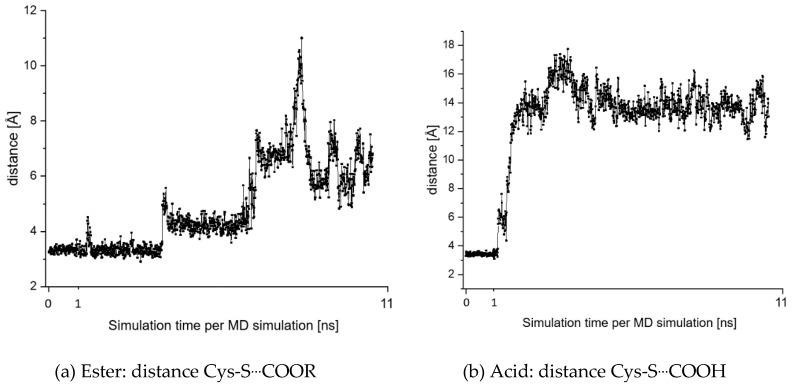
Fluctuations in the distance between the sulfur center of Cys-25 and (**a**) the carbon center of the ester group of compound **7** and (**b**) the carbon center of the acid group. The simulations were started from the pose given in Figure 7.

MD simulations were also performed for the reverse, i.e., substrate-like binding mode given in Figure 7. These simulations indicate a moderately stable complex. However, as shown in Figure 8a, the distance between the cysteine moiety and the ester group increases along the simulation time. Nevertheless, the ester may remain sufficiently long within the active site for an attack at the carbonyl carbon followed by ester hydrolysis. To investigate possible differences between the ester and the acid we performed an MD simulation starting from the pose given in Figure 7, but replaced the ester group by the corresponding acid group. Figure 8b shows that in this case, the inhibitor leaves the enzyme rapidly and the enzyme-inhibitor complex splits up completely. The behavior may result from the repulsion between the negatively charged carboxylate and the negatively charged thiolate moiety of Cys-25.

**Figure 9:**
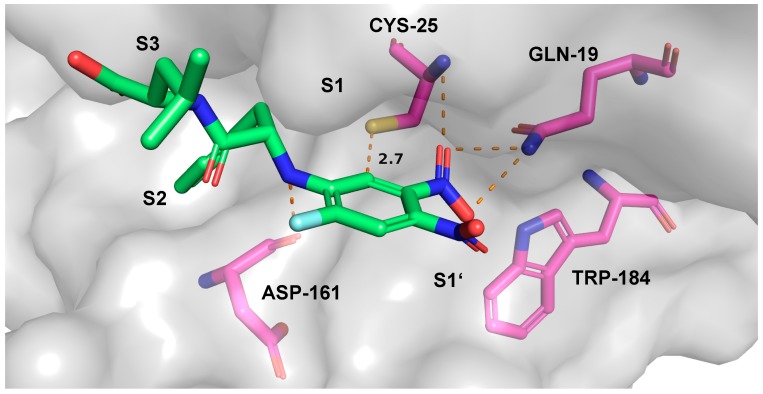
Predicted binding mode of acid **8**. Light grey: solvent accessible surface of rhodesain; magenta: carbon atoms of rhodesain amino acid residues; green: carbon atoms of acid **8**; blue: nitrogen; red: oxygen; yellow: sulfur; cyan: fluorine.

**Figure 10:**
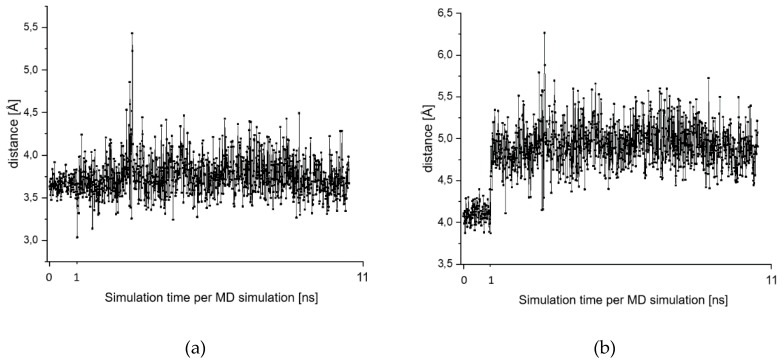

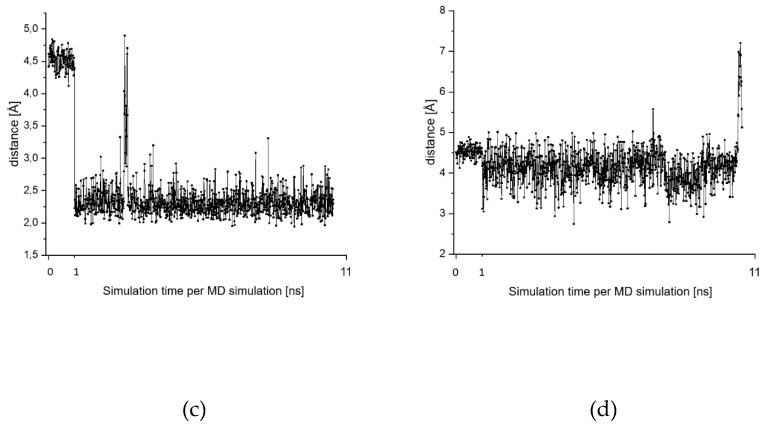
Charts (**a**,**b**) present the fluctuation of the distances between the sulfur center of Cys-25 and the carbon centers (CX) of the aromatic ring: (**a**) = distance S**^…^**CH; (**b**) = S**^…^**CNO_2_. (**c**,**d**) give the fluctuation in the distance of the Cys-S**^…^**H-N hydrogen bond along MD 5 (**c**) and MD 6 (**d**). All simulations start from the pose in Figure 9.

For acid **8** in the highest ranked docking pose, the electrophilic aromatic ring was found to be located close to the nucleophilic cysteine (distance 2.7 Å, score –18.03, Figure 9). The peptide backbone has interactions with Asp-161 and Gly-66, while the phenylalanine side chain occupies the hydrophobic S2 pocket. The leucine side-chain and the carboxylic acid group reside in the S3 pocket. One nitro group forms hydrogen bonds to Gln-19. To investigate the stability of the binding pose, we performed various MD simulations, which indicated a very stable complex. The corresponding fluctuations in the distances between the thiolate moiety and the CH- and CNO_2_-groups of the aromatic ring are depicted in Figure 10a,b, respectively.

**Figure 11:**
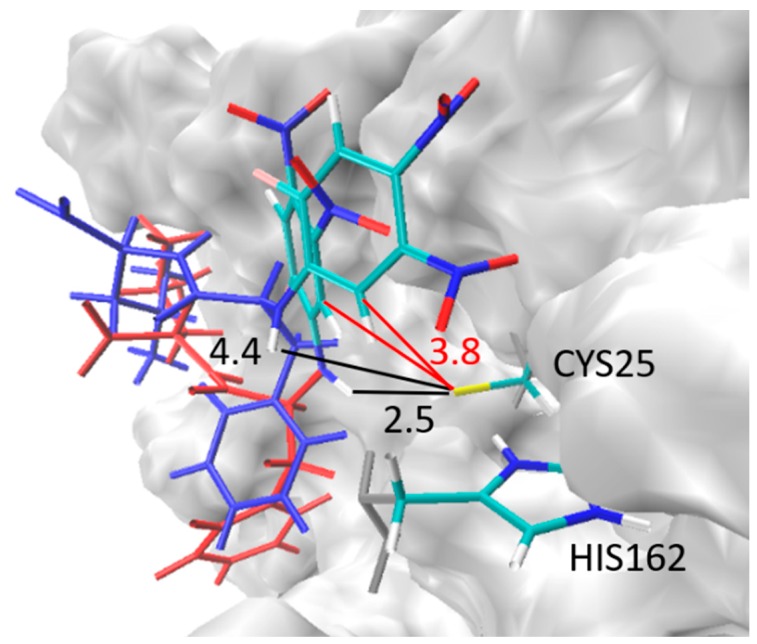
Sketch of the two principle conformations of acid **8** taken along the molecular dynamics (MD) simulations starting from the pose depicted in Figure 9. The electrophilic aromatic ring is given in atomic color while the rest of the inhibitor is given in blue or red. The distances between the S-center of Cys-25 and the NH group (Cys-S**^…^**H-N) are given in black, while the distances to the CH group of the aromatic ring (Cys-S**^…^**H-C) are given in red. For more information, see main text.

Our model computations indicate a stable pre-complex that is stabilized by a strong hydrogen bond between the thiolate and the NH substituent of the aromatic ring. To investigate whether this hydrogen bond is also important for the enzyme-inhibitor complex, we analyzed the distance Cys-S**^…^**H-N between the sulfur center of Cys-25 and the proton of the NH substituent along our MD simulations. The corresponding fluctuations are given in Figure 10c,d for two MD simulations, which reflect the two different conformations that are adopted along these MD simulations. Both conformations are depicted in Figure 11.

Within the pose depicted in Figure 9, the Cys-S**^…^**H-N distance is around 4.5 Å, i.e., all MD simulations started from that distance. For some MDs this conformation was kept for the whole simulation (Figure 10d, Figure 11 conformation with Cys-S**^…^**H-N = 4.4 Å). In other cases (Figure 10c, Figure 11 conformation with Cys-S**^…^**H-N = 2.5 Å), a presumably more stable conformation is adopted in which the Cys-S**^…^**H-N distance decreases strongly and fluctuates around 2.2 Å, indicating a strong hydrogen bond. While the distance Cys-S**^…^**H-N strongly differs between both conformations, the distances between the sulfur center of Cys-25 and the CH group of the substituted aromatic ring (Cys-S**^…^**H-C) remains constant. The latter indicates a strong interaction between the catalytic dyad Cys-25/His-162 and the electrophilic aromatic ring, which was also found for the model system (see Section 2.6). It is worth noting that for poses of the ester **7** in which the electrophilic aromatic ring was also in the vicinity of Cys-25 such a conformational change was not found. In the case of the ester, all poses stayed in conformations with quite long Cys-S**^…^**H-N distances. This indicates that the formation of the Cys-S**^…^**H-N bond is one reason for the strong inhibition potency of the acid, while the corresponding ester does not show a comparably strong inhibition.

A complete nucleophilic substitution reaction would lead to an irreversible inhibition because the eliminated group would diffuse away. Consequently, for a reversible inhibition the reaction must get trapped in a minimum from which the elimination of the leaving group cannot happen. This minimum cannot be a Meisenheimer complex as found for the model system with a single attacking thiolate because in this complex, the bonds to the leaving groups are already elongated, i.e., an elimination could occur. The π-complex found for the model system consisting of a thiolate/imidazolium unit instead of a single thiolate represents such a trap. It is more stable than the reactants and a further approach of the thiolate towards the inhibitor, which is necessary for an S_N_Ar reaction, cannot take place because the underlying potential is repulsive. The question remains whether a similar situation exists for the enzyme.

In many cases the enzyme environment strongly influences the reactivity of a given inhibitor [71,72,73]. To investigate the situation for the present question, possible reaction paths of the addition step of the thiolate moiety to the aromatic ring including the influence of the enzyme environment were computed. For this instance, hybrid QM/MM computations were performed in which the most important part is described quantum-mechanically (QM) while the influence of the rest is taken into account by force field approaches (MM) using the ONIOM (own n-layered integrated molecular orbital and molecular mechanics) approach implemented in GAUSSIAN16 [69]. Within these computations, the QM part consisted of 35 atoms. It was described by SCS-MP2/6-31 + G* because the ωB97XD values underestimated the effects in the model computation. The influence of the rest of the enzyme (more than 5200 atoms) was included into the MM part, which was described by the AMBER16 force field. The electrostatic embedding was employed to include possible polarization effects. For the calculations, some frames of the MD simulation performed for the complex of compound **8** and rhodesain were selected, and a full geometry optimization was conducted using QM/MM. The resulting geometry represents the starting point for a possible addition of the thiolate to the substituted aromatic ring as the first step of the S_N_Ar reaction. We computed the minimal energy path of the addition by decreasing the distance between the thiolate and the two reachable carbon centers (C–H and C–NO_2_). For each distance all other geometrical degrees of freedom were optimized. The results are depicted in Table 3.

Table 3 indeed indicates small minima in the reaction path. They appear at distances for R(S-CH) of about 3.1–3.3 Å or for R(S-CNO_2_) of about 3.9 Å, i.e., in the range of the complex of the model ”thiolate and imidazolium: full optimization” (Figure 6). They seem to be shallower than in the corresponding model system. However, we expect that parts of the attractive forces between the catalytic dyad and the substituted ring are already taken into account by the force field used for the MD simulations or in the subsequent QM/MM geometry optimization, i.e., they already stabilize the minimum from which the computation of the reaction paths started. Additionally, steric interactions may counteract. Nevertheless, beside the indicated strong Cys-S^…^H-N hydrogen bond (see above), the underlying interactions will contribute to the high inhibition potency of the acid **8** for rhodesain. The specific nature and the interplay of the various interactions still have to be deciphered. As for the model system, the computations for the enzyme environment also find a repulsive reaction path, so that a Meisenheimer complex cannot be formed. As a consequence, a complete substitution reaction cannot take place in the enzyme.

In summary, our QM/MM computations indicate that acid **8** and rhodesain form a very stable π-complex, which can explain the high inhibition potency of **8**. Because the reaction path of the formation of a Meisenheimer complex is repulsive, a S_N_Ar reaction as found for the reaction of 2-phenylethanethiol with the ester **7** in the presence of a base in ethanol cannot take place in the enzyme. This explains why acid **8** is a reversible inhibitor. The lower inhibitory potency of ester **7** is a consequence of the non-covalent interactions between inhibitor and enzyme, which prevent the formation of such a strong complex.

### 2.8. T. b. brucei Cell Survival Assay

**Figure 12:**
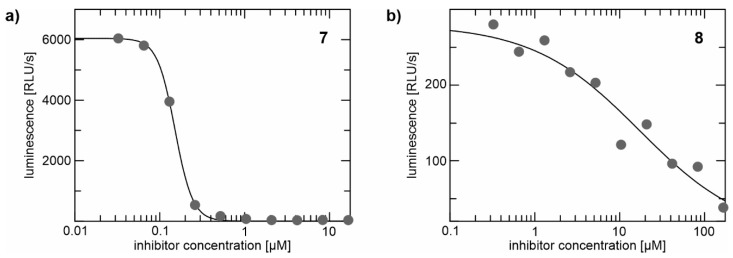
Anti-trypanosomal activity of the (**a**) benzyl ester **7** and (**b**) acid **8** measured by the ATPlite assay. Shown is luminescence (RLU/s = relative light unit per second) in dependence of compound concentration.

Ester **7** and acid **8** were tested for their anti-trypanosomal activity against *T. brucei brucei* using the ATPlite assay according to previously published procedures [74]. After 24 h, EC_50_ values of 0.0953 (±0.0402) µM for the ester **7** and 18.5 (±4.97) µM for the acid **8** were found (Figure 12). The differences in anti-protease and anti-trypanosomal activity between the lipophilic ester **7** and the more hydrophilic acid **8** might be due to different cell permeabilities of the compounds. With the acid **8** being the active protease inhibitor and the more lipophilic benzyl ester **7** being the better anti-trypanosomal compound that is converted to the active protease inhibitor **8** by the target enzyme itself, we presumably have discovered a new prodrug concept for anti-trypanosomal compounds targeting the major trypanosomal cysteine protease rhodesain. These results are in agreement with previous findings [75], which showed that especially rhodesain inhibitors with benzyl ester moieties display high anti-trypanosomal activity.

## 3. Discussion

The realm of potentially S*_N_*Ar-reactive units as building blocks for protease inhibitors is still largely unexplored and as stated in the introduction part, only very few steps have been undertaken in this direction. The evaluation of (hetero)aromatic electrophiles as possible inhibitory fragments or warheads for peptidic cysteine protease inhibitors is a first important step. Among the inhibitors presented herein, which contain the dipeptide HN-l-Phe-l-Leu-OBn as the recognition unit for cathepsin L-like proteases, the 2-fluoro-3-nitro-substituted benzoic acid amide **4** and the 2-fluoro-4,5-dinitro-substituted aniline derivative **7** were discovered as very potent cathepsin L inhibitors. Amide **4** additionally inhibits the trypanosomal cathepsin L-like protease rhodesain. The compounds were found to exhibit a competitive and reversible mode of inhibition. As shown by NMR spectroscopy, mass spectrometry and hydrolysis assays, the benzyl ester **7** was found to be hydrolyzed to the free acid **8** by rhodesain to yield a highly potent reversible rhodesain inhibitor (*K*_i_ = 4.0 nM). Thus, with the benzyl ester **7**, we identified an unexpected prodrug that is converted into the active protease inhibitor by the target enzyme itself. This is supported by the *T. b. brucei* cell survival assays, which revealed the benzyl ester **7** as a compound with significantly enhanced anti-trypanosomal activity. The serendipitous discovery of the dual mode of action of compound **8** implies that other ester- or even amide-type inhibitors may also react similarly. The ester **7** performed S_N_Ar reactions with 2-phenylethanethiol in ethanol in the presence of a base. Such a reaction is also expected for **8** because both possess the same substituted aromatic ring. However, because **8** acts as a reversible inhibitor, such a reaction cannot be the inhibition mechanism of rhodesain.

To investigate possible inhibition mechanisms, QM computations for relevant model systems were performed. Docking and MD simulation were conducted to detect and investigate possible inhibitor-enzyme complexes of the compounds **7** and **8**. Finally, QM/MM computations were carried out to get some insights into possible reactions between **8** and rhodesain.

The model calculations show that a single attacking thiolate behaves completely different than a dyad consisting of a thiolate and a positively charged imidazolium ring. The former is a model for the reaction of phenylethanethiol in ethanol in the presence of a base, while the latter mimics the catalytic dyad of the protease. For a single thiolate, the computations predict stable Meisenheimer complexes, which is in line with the observed S_N_Ar reactions. By contrast, for a thiolate/imidazolium dyad, a stable π-complex is found. The reaction potential of a further approach towards a Meisenheimer complex is repulsive, i.e., the reaction cannot take place. Consequently, while an irreversible reaction is predicted for a single thiolate, a reversible formation of a stable π-complex is computed for the thiolate/imidazolium dyad. Subsequent QM/MM computations simulating the reaction of **8** with rhodesain indicate a similar situation. Hence, we expect that an S_N_Ar reaction of acid **8** with rhodesain does not take place because the reaction is trapped in a very stable π-complex. The complex seems to receive additional stability through a strong hydrogen bridge between the thiolate group of Cys-25 and the NH-substituent of the aromatic ring.

The conducted docking and MD simulations reveal several stable complexes between ester **7** and rhodesain, in which the substituted aromatic ring sits in the active site, i.e., in close vicinity to the reactive dyad. Nevertheless, reactions are not expected because MD simulations show that the distances are still too large. Additionally, no indication for the formation of a stable π-complex or the thiolate-NH bridge is seen. Hence, we expect that these complexes are less stable, which nicely explains the lower inhibitory potency of ester **7** compared to acid **8**. Docking also predicts a reverse, substrate-like binding pose, which is ideal for the hydrolysis of the ester through rhodesain. MD simulations find this pose to be reasonably stable. For the acid **8** in rhodesain, docking and MD find very stable complexes in which the substituted aromatic ring and the catalytic Cys-25/His-162 dyad are sufficiently close. However, as described above, the reaction path towards the Meisenheimer complex only exhibits shallow minima for large distances (R(S-CH) or R(S-CNO_2_) ≈ 3.1 – 4.0 Å) while it is repulsive for a further approach. This finding is in line with the reversible inhibition of rhodesain by acid **8**.

In summary, with the benzyl ester **7**, we present an efficient prodrug that has a strong anti-trypanosomal effect and inhibits the trypanosomal protease rhodesain in vitro via the hydrolysis and subsequent reversible binding of the hydrolysis product **8** via a π-complex. In future studies, we will address the question if esters other than benzyl can also be used as prodrugs.

## 4. Materials and Methods

See Supplementary Materials.

## Figures and Tables

**Table 1:** Inhibition data for inhibitors of the general structure R-NH-l-Phe-l-Leu-OBn, % inhibition at 20 µM and ***K*_i_** [µM] ^1^.

Cpd.	Cath. L	Cath. B	Rhod.	DENV PR	Sortase A
	% inh./*K*_i_	% inh./*K*_i_	% inh./*K*_i_	% inh./*K*_i_	% inh./*K*_i_
**1**	57/nd	16/nd	nd/nd	19/nd	nd/nd
**2**	56/nd	33/nd	nd/nd	24/nd	nd/nd
**3**	60/nd	22/nd	nd/nd	28/nd	nd/nd
**4**	99/0.66	20/nd	75/0.29	10/nd	11/nd
**5**	8/nd	10/nd	nd/nd	11/nd	nd/nd
**6**	50/nd	21/nd	nd/nd	12/nd	nd/nd
**7**	98/1.6	63/4.4	45/nd	31/nd	16/nd

^1^ All values are from at least three independent measurements; standard deviations are 10% or less; nd, not determined; cpd., compound; Cath. L, cathepsin L; Cath. B, cathepsin B; Rhod.; rhodesain, DENV PR, Dengue virus protease.

**Table 2:** Stabilities (only thiolate/thiolate and imidazolium: full optimization/thiolate and imidazolium: R(S-CX) fixed at 1.9 Å) of the Meisenheimer complexes in a polar solvent [in kcal/mol]. For all calculations the 6-31 + G* basis sets were employed. For further information see main text and Figure 6.

Structure	ωB97XD	MP2	SCS-MP2
Pre-complex	–8.8/–9.0/	–12.3/–16.0/-	–10.3/–12.8/
S-CH	+0.8/–11.5/–6.8	–6.6/–16.0/–12.8	–4.1/–12.9/–9.3
S-CF	–3.3/–13.1/–3.1	–13.2/–20.3/–12.5	–11.1/–16.5/–10.1
S-NO_2_	–2.7/–9.3/+3.3	–11.9/–17.0/–5.3	–9.1/–13.3/–2.9

**Table 3:** Shapes of various addition–reaction pathways of the Cys moiety to the substituted aromatic rings. Relative energies [kcal/mol] are given with respect to the full geometry optimization. The full optimization started from the indicated MD frame.

Starting Structure	R(S-CNO_2_)	R(S-CH)	ΔE
MD 6 Frame 963 conformer with short Cys-S^…^H-N distance		3.7	0.0
		3.3	1.4
		3.1	1.7
		2.9	2.5
MD 6 Frame 1096 conformer with short Cys-S^…^H-N distance		3.7	0.0
		3.3	0.7
		3.1	0.8
		2.9	2.6
MD 6 Frame 542 conformer with short Cys-S^…^H-N distance	4.9		0.0
	4.5		1.2
	4.1		3.4
	3.9		0.4
	3.5		5.3

## Supplementary Materials

The following are available online at https://www.mdpi.com/1420-3049/25/6/1451/s1: Syntheses and analytical data of the compounds; Analysis of the reaction products of the reaction with low-molecular weight thiol; Calculation of the chemical shifts/determination of the regiochemistry of the addition products; Mass spectrometry; NMR material and methods; Enzyme and hydrolysis assays; *T. b. brucei* cell survival assay; Docking procedures; NMR spectra of the compounds.
