# Supplementary material for: New Cysteine Protease Inhibitors: Electrophilic (Het)arenes and Unexpected Prodrug Identification for the Trypanosoma Protease Rhodesain
Source: Molecules. 2020 Mar 23;25(6):1451. doi: 10.3390/molecules25061451 (PMC7145299; doi:10.3390/molecules25061451)
Supplement: Supplementary file 1 [file molecules-25-01451-s001.pdf]

molecules

Supplementary Material

New Cysteine Protease Inhibitors: Electrophilic (Het)arenes and Unexpected Prodrug Identification for the *Trypanosoma* Protease Rhodesain

Philipp Klein [1,†], Patrick Johe [2,†], Annika Wagner [3], Sascha Jung [2,4], Jonas Kühlborn [1], Fabian Barthels [2], Stefan Tenzer [5], Ute Distler [5], Waldemar Waigel [6], Bernd Engels [6], Ute A. Hellmich [3,7], Till Opatz [1,*] and Tanja Schirmeister [2,*]

[1] Department of Chemistry, Organic Chemistry Section, Johannes Gutenberg-Universität, 55128 Mainz, Germany; klein@uni-mainz.de (P.K.); jokuehlb@uni-mainz.de (J.K.)
[2] Institute of Pharmaceutical and Biomedical Sciences, Johannes Gutenberg-Universität, 55128 Mainz, Germany; pajohe@uni-mainz.de (P.J.); sascha.jung@tu-dortmund.de (S.J.); barthels@uni-mainz.de
[3] Department of Chemistry, Biochemistry Section, Johannes Gutenberg-Universität, 55128 Mainz, Germany; a.wagner@uni-mainz.de (A.W.); u.hellmich@uni-mainz.de (U.A.H.)
[4] Present address: Faculty of Chemistry and Chemical Biology, TU Dortmund University, 44227 Dortmund, Germany
[5] Institute of Immunology, University Medical Center, Johannes Gutenberg-Universität Mainz, 55131 Mainz, Germany; tenzer@uni-mainz.de (S.T.); ute.distler@uni-mainz.de (U.D.)
[6] Institute of Physical and Theoretical Chemistry, Universität Würzburg, 97074 Würzburg, Germany; waldemar.waigel@uni-wuerzburg.de (W.W.); bernd.engels@uni-wuerzburg.de (B.E.)
[7] Centre for Biomolecular Magnetic Resonance (BMRZ), Goethe-University Frankfurt, 60323 Frankfurt Germany
*Correspondence: opatz@uni-mainz.de (T.O.); schirmei@uni-mainz.de (T.S.); Tel.: +49-(0)6131-39-22272 (T.O.); +49-(0)6131-39-25742 (T.S.)
† Both authors contributed equally.

Table of Contents

1. Syntheses and analytical data of the compounds 2
2. Analysis of the Reaction Products of the Reaction with LMW Thiol 10
3. Calculation of the Chemical Shifts / Determination of the Regiochemistry of the Addition Products 12
4. Mass spectrometry 13
5. NMR material and methods 13
6. Enzyme and hydrolysis assays 13
7. *T. b. brucei* cell survival assay 15
8. Docking procedures 16
9. NMR Spectra of the compounds 17

1. **Syntheses and analytical data of the compounds**

1.1. General Information

All reagents and solvents were obtained from commercial suppliers (Sigma Aldrich, Alfa Aesar, TCI chemicals, ABCR, Acros Organics and Fischer Scientific) and used without further purification. Flash column chromatography was performed using silica gel type 60 M (230-400 mesh, Macherey Nagel). Analytical thin-layer chromatography (TLC) was done on Merck silica gel plates (60 F_{254}) with defined solvent mixtures and visualized under UV light irradiation and/or TLC staining reagents. Melting points were determined in open capillary tube. IR spectra were measured with a JASCO (FT/IR-4100) with a diamond ATR unit and are reported in terms of frequency of absorption (ν, cm^{-1}). NMR experiments were performed on a 300 MHz (300 MHz ^{1}H and 75 MHz ^{13}C), a 400 MHz (400 MHz ^{1}H and 101 MHz ^{13}C) or a 600 MHz (600 MHz ^{1}H and 151 MHz ^{13}C) spectrometer from Bruker using deuterated solvents ((residual) solvent signals: $CDCl_3$: δH = 7.26 ppm, δC = 77.16 ppm; $(CD_3)_2SO$: δH = 2.50 ppm, δC = 39.52 ppm) as internal references and reported in parts per million (ppm, δ) relative to tetramethylsilane (TMS, δ = 0.00 ppm)[1]. Coupling constants (*J*) are reported in Hz, and the multiplet abbreviations used were: s, singlet; d, doublet; t, triplet; m, multiplet; br, broad; app, apparent; and combinations thereof. Electrospray ionization (ESI-) mass spectra were recorded on an Agilent/Bruker LC/MSD trap XCT spectrometer or on a 1200-series HPLC-system (Agilent-Technologies) with binary pump, integrated diode array detector and Ascentis Express C18 column (2.7 µm, 30x2.1 mm). ESI-HRMS spectra were recorded on a Waters Q-TOF-Ultima 3 instrument or an Agilent 6545 QTOF-MS mass spectrometer. Field desorption (FD-) mass spectra were measured on a Finnigan MAT 95 spectrometer. Preparative reverse phase separations were carried out on an Agilent 1290 Infinity II preparative system with a 1290 Infinity II preparative binary pump, 1260 Infinity II DAD and a 1290 Infinity II fraction collector. Optical rotations were measured with a Perkin–Elmer 241 polarimeter at 546 and 579 nm. Extrapolation to 589 nm was performed according to Thaler et al.[2].

1.2. Benzyl-N-(Tert-Butoxycarbonyl)-L-Phenylalanyl-L-Leucinate (A1)

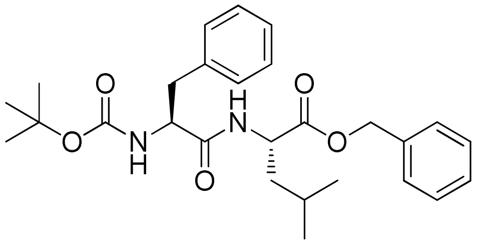

The product was synthesized according to Lawesson et al.[3]. In an inert gas atmosphere, *N*-Boc-L-phenylalanine (0.67 g, 2.54 mmol), L-leucine benzyl ester *p*-toluenesulfonate salt (1.00 g, 2.54 mmol) and triethylamine (360 µL, 2.54 mmol) were dissolved in dichloromethane (7 mL). The suspension was cooled to –18 °C and *N,N′*-dicyclohexylcarbodiimide (0.52 g, 2.54 mmol) was added. The resulting mixture was stirred for 1 h at that temperature and then 20 h at room temperature. The mixture was filtered off and the solvent was removed under reduced pressure. The residue was dissolved in ethyl acetate (10 mL) and washed with hydrochloric acid (0.1 M, 2.5 mL), sodium bicarbonate (0.5 M, 2.5 mL) and brine (2.5 mL). The organic phase was dried over magnesium sulfate and the solvent was removed under reduced pressure. The title compound was obtained as a colorless solid (970 mg, 2.07 mmol, 81 %) by crystallization from diethyl ether:petroleum ether (1:3 v/v, 4 mL).

R_f = 0.70 (petroleum ether:ethyl acetate, 2:1).

^{1}H-NMR, COSY (300 MHz, $CDCl_3$) δ = 7.43–7.15 (m, 10H, H^{arom}), 6.28 (d, *J* = 8.2 Hz, 1H, N*H*), 5.15 (d, *J* = 12.3 Hz, 1H, OCH_2^{Bn}), 5.10 (d, *J* = 12.3 Hz, 1H, OCH_2^{Bn}), 5.00 (br s, 1H, N*H*), 4.61 (m, 1H), 4.34 (dd, *J* = 7.3, 6.3 Hz, 1H), 3.05 (d, *J* = 6.8 Hz, 2H, β-CH_2^{Phe}), 1.67–1.43 (m, 3H, γ-CH^{Leu}, β-CH_2^{Leu}), 1.42 (s, 9H, CH_3^{Boc}), 0.88 (*d*, *J* = 6.1 Hz, 3H, δ-CH_3^{Leu}), 0.86 (*d*, *J* = 6.1 Hz, 3H, δ-CH_3^{Leu}) ppm.

^{13}C-NMR, HMBC, HSQC (75 MHz, $CDCl_3$) δ = 172.4 (*C* = O), 171.0 (*C* = O), 155.5 (*C* = O^{Boc}), 136.7 (γ-C^{Leu}), 135.5 (C_q^{Bn}), 129.5, 128.8, 128.7, 128.5, 128.4, 127.1 (CH^{arom}), 80.4 ($C^{Boc,q}$), 67.1 (CH_2^{Bn}), 55.8 (α-CH^{Phe}), 51.0 (α-CH^{Leu}), 41.7 (β-CH_2^{Leu}), 38.2 (β-CH_2^{Phe}), 28.4 (CH_3^{Boc}), 24.8 (γ-CH^{Leu}), 22.9, 22.0 (δ-CH_3^{Leu}) ppm.

ESI-MS (*m/z*): 469.3 (100) $[M + H]^+$, 491.3 (28) $[M + Na]^+$.

The spectroscopic data are in accordance with literature [3].

1.3. Benzyl-L-Penylalanyl-L-Leucinate Hydrochloride Salt (A2)

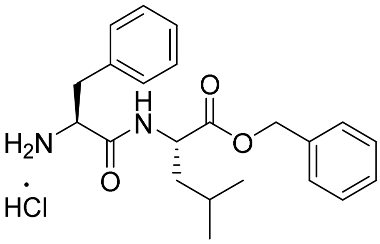

The deprotection was carried out similar to Hruby et al. [4]. In an inert gas atmosphere, the dipeptide **A1** (500 mg, 1.07 mmol) was added to HCl/ dioxane (4 M, 5 mL) at 0 °C. The ice bath was removed and the solution was stirred additional 30 min. The solvent was removed under reduced pressure, the product filtered off and washed with diethyl ether to produce a colorless solid (430 mg, 1.06 mmol, 99 %, Lit.[4]: <99 %) which was used for subsequent transformations without further purification.

^{1}H-NMR, COSY (300 MHz, DMSO-d_6) δ = 9.03 (d, *J* = 7.6 Hz, 1H, NH^{Leu}), 8.19 (br s, 3H, NH_3^{Phe}), 7.45–7.21 (m, 10H, H^{Ar}), 5.17 (d, *J* = 12.4 Hz, 1H, OCH_2^{Bn}), 5.11 (d, *J* = 12.4 Hz, 1H, OCH_2^{Bn}), 4.47–4.33 (m, 1H, α-CH^{Leu}), 4.07 (dd, *J* = 8.0, 5.2 Hz, 1H, α-CH^{Phe}), 3.11 (dd, *J* = 14.1, 5.2 Hz, 1H, β-CH_2^{Phe}), 2.89 (dd, *J* = 14.1, 8.1 Hz, 1H, β-CH_2^{Phe}), 1.61 (m, 3H, β-CH_2^{Leu}, γ-CH^{Leu}), 0.90 (d, *J* = 6.4 Hz, 3H, δ-CH_3^{Leu}), 0.86 (d, *J* = 6.3 Hz, 3H, δ-CH_3^{Leu}) ppm.

The spectroscopic data are in accordance with literature.[4]

1.4. Benzyl-L-Penylalanyl-L-Leucinate Trifluoroacetic Acid Salt (A5)

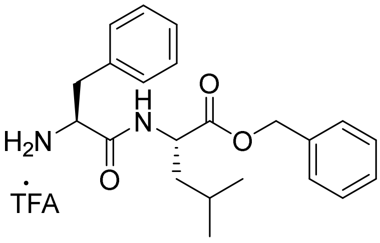

The product was obtained by deprotection with trifluoroacetic acid. The dipeptide **A1** (113 mg, 0.24 mmol) was added to a mixture of TFA (1 mL) and dichloromethane (2 mL) at 0 °C. After 10 min. the ice bath was removed and the solution was stirred for 2 h at room temperature. The solvent was removed under reduced pressure and the product (116 mg, 0.24 mmol, >99 %) was used without further purification.

^{1}H-NMR (300 MHz, DMSO-d_6) δ = 8.89 (d, *J* = 7.7 Hz, 1H, NH^{Leu}), 8.11 (br s, 3H, NH_3^{Phe}), 7.37 – 7.13 (m, 10H, H^{Ar}), 5.17 (d, *J* = 12.4 Hz, 1H, OCH_2^{Bn}), 5.11 (d, *J* = 12.4 Hz, 1H, OCH_2^{Bn}), 4.41 (q, *J* = 7.5 Hz, 1H), 4.03 (dd, *J* = 8.3, 5.0 Hz, 1H), 3.08 (dd, *J* = 14.2, 4.9 Hz, 1H, β-CH_2^{Phe}), 2.86 (dd, *J* = 14.2, 8.4 Hz, 1H, β-CH_2^{Phe}), 1.70 – 1.53 (m, 3H, β-CH_2^{Leu}, γ-CH^{Leu}), 0.91 (d, *J* = 6.2 Hz, 3H, δ-CH_3^{Leu}), 0.86 (d, *J* = 6.2 Hz, 3H, δ-CH_3^{Leu}) ppm.

1.5. Benzyl-N-[(2,6-Dichloropyridin-3-yl)Carbonyl]-L-Phenylalanyl-L-Leucinate (1)

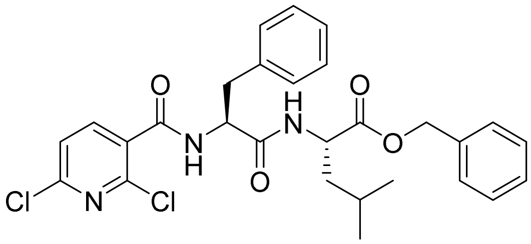

2,6-Dichloronicotinoyl chloride (100 mg, 0.54 mmol) and the hydrochloride of the deprotected dipeptide **A2** (216 mg, 0.54 mmol) were dissolved in dichloromethane (3 mL). The solution was cooled to 0 °C and triethylamine (152 µL, 1.10 mmol) in dichloromethane (1 mL) was added dropwise. The solution was stirred 1 h at 0 °C and 1 h at room temperature. Then the organic layer was washed with water, dried over magnesium sulfate and the solvent removed under reduced pressure. Purification by chromatography (SiO_2, 100 % pentane to pentane:ethyl acetate (4:1)) afforded the title compound (214 mg, 0.38 mmol, 73 %) as a colorless solid.

$\mathbf{R_f}$ = 0.27 (petroleum ether:ethyl acetate, 3:1).

mp: 68–70°C.

^{1}H-NMR, COSY (300 MHz, $CDCl_3$) δ = 7.91 (d, *J* = 8.0 Hz, 1H, H-4^{Pyr}), 7.43–7.18 (m, 12H, H^{Ar}), 6.22 (d, *J* = 8.0 Hz, 1H, NH^{Leu}), 5.17 (d, *J* = 12.4 Hz, 1H, OCH_2^{Bn}), 5.13 (d, *J* = 12.4 Hz, 1H, OCH_2^{Bn}), 4.87 (dd, *J* = 14.2, 7.2 Hz, 1H, α-CH^{Phe}), 4.58 (m, 1H, α-CH^{Leu}), 3.26–3.07 (m, 2H, β-CH_2^{Phe}), 1.66–1.37 (m, 3H, β-CH_2^{Leu}, γ-CH^{Leu}), 0.86 (d, *J* = 6.3 Hz, 3H, δ-CH_3^{Leu}), 0.86 (d, *J* = 6.2 Hz, 3H, δ-CH_3^{Leu}) ppm.

^{13}C-NMR, HMBC, HSQC (75 MHz, $CDCl_3$) δ = 172.0 (C = O^{Leu}), 169.8 (C = O^{Phe}), 163.5 (C = O^{Pyr}), 152.0 (C-Cl^{Pyr}), 146.6 (C-Cl^{Pyr}), 141.8 (C-4^{Pyr}), 136.0 (C_q^{Phe}), 135.2 (C_q^{Bn}), 129.4, 129.1, 128.8, 128.6, 128.5, 128.3, 127.3 (CH^{Ar}), 123.4 (C-5^{Pyr}), 67.2 (CH_2^{Bn}), 55.2 (α-CH^{Phe}), 51.2 (α-CH^{Leu}), 41.3 (β-CH_2^{Leu}), 38.2 (β-CH_2^{Phe}), 24.8 (γ-CH^{Leu}), 22.7, 21.9 (δ-CH_3^{Leu}) ppm.

IR: ν = 3285, 2924, 2853, 1743, 1642, 1577, 1545, 1427, 1382, 1341, 1240, 1188, 1119, 879, 798, 744, 698 cm^{-1}.

ESI-HRMS (*m/z*) calculated for $[C_{28}H_{30}N_3O_4{}^{35}Cl_2]^+$ = 542.1613, found 542.1606.

$[\alpha]_D^{26}$ = –1.4° (c = 1.0, CH_2Cl_2).

1.6. Benzyl-N-(6-Chloropyrimidin-4-yl)-L-Phenylalanyl-L-Leucinate (2)

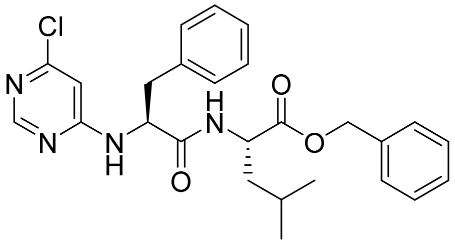

To a solution of 4,6-dichloropyrimidine (140 mg, 0.90 mmol) and the hydrochloride of the deprotected dipeptide **A2** (182 mg, 0.45 mmol) in dichloromethane (3 mL) triethylamine (60 µL, 0.45 mmol) in dichloromethane (1 mL) was added dropwise and the resulting mixture was refluxed for 48 h. The solution was diluted with additional dichloromethane and washed with water. After phase separation, the organic layer was dried over magnesium sulfate and the solvent was removed under reduced pressure. Purification by chromatography (SiO_2, 100 % pentane to pentane:ethyl acetate (4:1)) afforded the title compound (70 mg, 0.17 mmol, 38 %) as a colorless solid.

$\mathbf{R_f}$ = 0.11 (petroleum ether:ethyl acetate, 5:1).

mp: 61–64°C.

^{1}H-NMR, COSY (300 MHz, $CDCl_3$) δ = 8.34 (s, 1H, *H*-2^{Pyr}), 7.29 (m, 10H, *H*^{Ar}), 6.35 (s, 1H, *H*-5^{Pyr}), 6.26 (d, *J* = 8.2 Hz, 1H, N*H*^{Leu}), 5.90 (d, *J* = 7.2 Hz, 1H, N*H*^{Phe}), 5.16 (d, *J* = 12.2 Hz, 1H, OC*H*$_2^{Bn}$), 5.10 (dd, *J* = 12.2 Hz, 1H, OC*H*$_2^{Bn}$), 4.72 (br s, 1H, α-C*H*^{Phe}), 4.66–4.53 (m, 1H, α-C*H*^{Leu}), 3.24–3.01 (m, 2H, β-C*H*$_2^{Phe}$), 1.64–1.40 (m, 3H, β-C*H*$_2^{Leu}$, γ-C*H*^{Leu}), 0.86 (d, *J* = 6.3 Hz, 3H, δ-C*H*$_3^{Leu}$), 0.85 (d, *J* = 6.2 Hz, 3H, δ-C*H*$_3^{Leu}$) ppm.

^{13}C-NMR, HMBC, HSQC (75 MHz, $CDCl_3$) δ = 172.2 (*C* = O^{Leu}), 170.9 (*C* = O^{Phe}), 162.4 (*C*-6^{Pyr}), 159.5 (*C*-4^{Pyr}), 158.5 (*C*-2^{Pyr}), 136.5 (*C*$_q^{Phe}$), 135.3 (*C*$_q^{Bn}$), 129.4, 128.9, 128.8, 128.7, 128.4, 127.4 (CH^{Ar}), 104.4 (*C*-5^{Pyr}), 67.3 (CH$_2^{Bn}$), 56.3 (α-CH^{Phe}), 51.2 (α-CH^{Leu}), 41.6 (β-CH$_2^{Leu}$), 38.4 (β-CH$_2^{Phe}$), 24.9 (γ-CH^{Leu}), 22.8, 22.0 (δ-CH$_3^{Leu}$) ppm.

IR: ν = 3280, 3031, 2957, 1741, 1661, 1582, 1496, 1455, 1387, 1326, 1270, 1186, 1148, 1091, 983, 910, 842, 736, 697, 654 cm^{-1}.

ESI-HRMS (*m*/*z*) calculated for $[C_{26}H_{29}N_4O_3Na^{35}Cl]^+$ = 503.1826, found 503.1821; calculated for $[C_{26}H_{29}N_4O_3Na^{37}Cl]^+$ = 505.1796, found 505.1784.

$[\alpha]_D^{24}$ = −14.5° (c = 1.0, CH_2Cl_2).

1.7. Benzyl-N-(5-Fluoro-2,4-Dinitrophenyl)-L-Phenylalanyl-L-Leucinate (3)

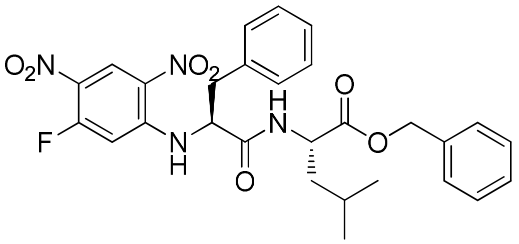

A solution of *N,N*-diisopropylethylamine (85 μL, 0.50 mmol) in ethanol (3 mL) was added dropwise to a solution of 1,3-difluoro-4,6-dinitrobenzene (51 mg, 0.25 mmol) and the hydrochloride of the deprotected dipeptide **A2** (100 mg, 0.25 mmol) in ethanol (3 mL). The resulting mixture was stirred at room temperature for 10 h and quenched with water. The aqueous phase was extracted with ethyl acetate (15 mL), dried over $MgSO_4$ and the solvent evaporated. The crude product was dissolved in ethanol and precipitated by slow addition of petroleum ether and cooling to afford pure product (136 mg, 0.25 mmol, 98 %) as a yellow solid.

R$_f$ = 0.24 (petroleum ether:ethyl acetate, 5:1).

mp: 120–124 °C.

^{1}H-NMR, COSY (300 MHz, $CDCl_3$) δ = 9.06 (d, *J* = 7.9 Hz, 1H, *H*-3^{Head}), 8.80 (d, *J* = 4.8 Hz, 1H, N*H*^{Phe}), 7.38–7.24 (m, 10H, *H*^{Ar}), 6.46 (d, *J* = 13.0 Hz, 1H, *H*-6^{Head}), 6.20 (d, *J* = 8.5 Hz, 1H, N*H*^{Leu}), 5.13 (d, *J* = 12.2 Hz, 1H, OC*H*$_2^{Bn}$), 5.07 (d, *J* = 12.2 Hz, 1H, OC*H*$_2^{Bn}$), 4.75–4.61 (m, 1H, α-C*H*^{Leu}), 4.29–4.20 (m, 1H, α-C*H*^{Phe}), 3.33 (dd, *J* = 14.1, 5.2 Hz, 1H, β-C*H*$_2^{Phe}$), 3.23 (dd, *J* = 14.1, 7.4 Hz, 1H, β-C*H*$_2^{Phe}$), 1.60–1.40 (m, 3H, β-C*H*$_2^{Leu}$, γ-C*H*^{Leu}), 0.92 (d, *J* = 6.1 Hz, 3H, δ-C*H*$_3^{Leu}$), 0.90 (d, *J* = 6.1 Hz, 3H, δ-C*H*$_3^{Leu}$) ppm.

^{13}C-NMR, HMBC, HSQC (75 MHz, $CDCl_3$) δ = 172.1 (*C* = O^{Leu}), 169.3 (*C* = O^{Phe}), 160.0 (d, *J* = 272.5 Hz, *C*-F), 148.3 (d, *J* = 13.2 Hz, *C*-1^{Head}), 135.1 (*C*$_q^{Bn}$), 134.7 (*C*$_q^{Phe}$), 129.4 (d, *J* = 1.9 Hz, *C*-2^{Head}), 128.8, 128.7, 128.3 (*C*H^{Ar}), 127.9 (d, *J* = 35.2 Hz, *C*-4^{Head}), 127.1 (d, *J* = 9.0 Hz, *C*-3^{Head}), 102.8 (d, *J* = 27.0 Hz, *C*-6^{Head}), 67.5 (CH$_2^{Bn}$), 60.2 (α-CH^{Phe}), 51.2 (α-CH^{Leu}), 41.2 (β-CH$_2^{Leu}$), 38.9 (β-CH$_2^{Phe}$), 25.0 (γ-CH^{Leu}), 22.9, 21.8 (δ-CH$_3^{Leu}$) ppm.

IR: ν = 3347, 1752, 1634, 1587, 1519, 1455, 1418, 1337, 1297, 1209, 1190, 1152, 1050, 945, 920, 843, 739, 697 cm^{-1}.

ESI-HRMS (*m*/*z*) calculated for $[C_{28}H_{29}N_4O_7FNa]^+$ = 575.1918, found 575.1907.

$[\alpha]_D^{25}$ = −14.1° (c = 1.0, CH_2Cl_2).

1.8. Benzyl-N-(2-Fluoro-3-Nitrobenzoyl)-L-Phenylalanyl-L-Leucinate (4)

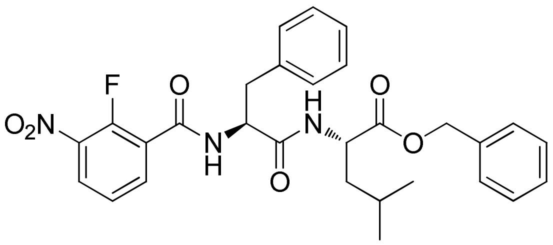

To a solution of 2-fluoro-3-nitrobenzoic acid (56 mg, 0.30 mmol) in toluene (2 mL) thionyl chloride (1 mL, 13.8 mmol) was added. The solution was heated to 80 °C for 5 h. After removal of the solvent, the product crystallized as a colorless solid (61 mg, 0.30 mmol, quant.). The product was used without further purification in the following step. The hydrochloride of dipeptide **A2** (145 mg, 0.30 mmol) was dissolved in dichloromethane (5 mL) and triethylamine (55 µL, 0.40 mmol) was added. After 10 min, a solution of 2-fluoro-3-nitrobenzoyl chloride (61 mg, 0.30 mmol) in dichloromethane (1 mL) was added dropwise. The reaction mixture was stirred at room temperature for 24 h before removing the solvent under reduced pressure. Purification by chromatography (SiO_2, dichloromethane:ethyl acetate (30:1)) afforded the title compound (118 mg, 0.22 mmol, 72 %) as a colorless solid.

$\mathbf{R_f}$ = 0.36 (petroleum ether:ethyl acetate, 5:2).

mp: 140–143 °C.

^{1}H-NMR, COSY (400 MHz, $CDCl_3$) δ = 8.24 (ddd, *J* = 8.1, 6.4, 1.9 Hz, 1H, *H*-6^{Ar}), 8.14 (ddd, *J* = 8.1, 7.1, 1.9 Hz, 1H, *H*-4^{Ar}), 7.46–7.18 (m, 12H, *H*^{Ar}, N*H*^{Phe}), 6.18 (d, *J* = 8.0 Hz, 1H, N*H*^{Leu}), 5.18 (d, *J* = 12.2 Hz, 1H, OC*H*$_2$^{Bn}), 5.14 (d, *J* = 12.2 Hz, 1H, OC*H*$_2$^{Bn}), 4.95–4.83 (m, 1H, *α*-C*H*^{Phe}), 4.66–4.53 (m, 1H, *α*-C*H*^{Leu}), 3.22 (dd, *J* = 13.9, 6.3 Hz, 1H, β-C*H*$_2$^{Phe}), 3.12 (dd, *J* = 13.9, 7.4 Hz, 1H, β-C*H*$_2$^{Phe}), 1.66–1.39 (m, 3H, β-C*H*$_2$^{Leu}, γ-C*H*^{Leu}), 0.86 (d, *J* = 6.3 Hz, 3H, δ-C*H*$_3$^{Leu}), 0.85 (d, *J* = 6.3 Hz, 3H, δ-C*H*$_3$^{Leu}) ppm.

^{13}C-NMR, HMBC, HSQC (101 MHz, $CDCl_3$) δ = 172.2 (*C* = O^{Leu}), 169.9 (*C* = O^{Phe}), 161.3 (d, *J* = 2.3 Hz, *C* = O^{Head}), 153.5 (d, *J* = 265.8 Hz, *C*-F), 138.2 (d, *J* = 10.0 Hz, *C*-3^{Head}), 137.1 (d, *J* = 2.9 Hz, *C*-6^{Head}), 136.1 (C_q^{Phe}), 135.4 (C_q^{Bn}), 129.5 (*C*H^{Ar}), 129.2 (d, *J* = 1.8 Hz, *C*-4^{Head}), 129.0, 128.8, 128.7, 128.4, 127.4 (CH^{Ar}), 124.8 (d, *J* = 4.8 Hz, *C*-5^{Head}), 124.0 (d, *J* = 11.8 Hz, *C*-1^{Head}), 67.3 (CH_2^{Bn}), 55.5 (*α*-CH^{Phe}), 51.3 (*α*-CH^{Leu}), 41.5 (β-CH_2^{Leu}), 38.5 (β-CH_2^{Phe}), 24.9 (γ-CH^{Leu}), 22.8, 22.1 (δ-CH_3^{Leu}) ppm.

IR: ν = 3262, 1733, 1640, 1615, 1535, 1456, 1389, 1327, 1219, 1189, 1136, 1081, 1014, 956, 854, 822, 744, 697 cm^{-1}.

ESI-HRMS (*m*/*z*) calculated for $[C_{29}H_{30}FN_3O_6Na]^+$ = 558.2016, found 558.2016.

$[\alpha]_D^{24}$ = –17.4° (c = 1.0, CH_2Cl_2).

1.9. Benzyl-N-[(4-Fluoro-3-Nitrophenyl)Carbamoyl]-L-Phenylalanyl-L-Leucinate (5)

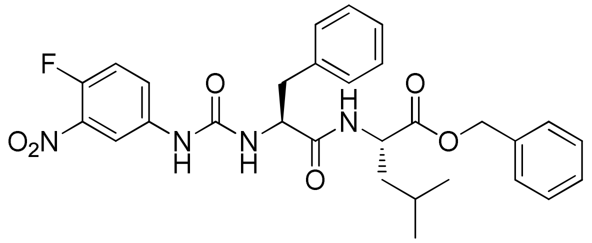

In an inert gas atmosphere, 4-fluoro-3-nitrophenylisocyanate (48 mg, 0.25 mmol) and the hydrochloride of the deprotected dipeptide **A2** (100 mg, 0.25 mmol) were dissolved in dichloromethane (3 mL). To this solution, *N,N*-diisopropylethylamine (42 µL, 0.25 mmol) in dichloromethane (1 mL) was added dropwise and the solution was stirred for 48 h. The solvent was removed under reduced pressure and the residue taken up in ethyl acetate and washed with water. The organic layer was dried over $MgSO_4$ and the solvent removed in vacuo. Crystallization from ethanol afforded the title compound (128 mg, 0.23 mmol, 93 %) as a colorless solid.

$\mathbf{R_f}$ = 0.69 (petroleum ether:ethyl acetate, 1:1).

mp: 169–172°C.

^{1}H-NMR, COSY (300 MHz, DMSO-d$_6$) δ = 9.14 (s, 1H, NH^{Head}), 8.63 (d, *J* = 7.8 Hz, 1H, NH^{Leu}), 8.31 (dd, *J* = 6.8, 2.7 Hz, 1H, *H*-3^{Head}), 7.51 (ddd, *J* = 9.1, 4.1, 2.7 Hz, 1H, *H*-6^{Head}), 7.43 (dd, *J* = 10.9, 9.1 Hz, 1H, *H*-5^{Head}), 7.38 – 7.13 (m, 10H, H^{Ar}), 6.44 (d, *J* = 8.4 Hz, 1H, NH^{Phe}), 5.17 (d, *J* = 12.6 Hz, 1H, OCH_2^{Bn}), 5.12 (d, *J* = 12.6 Hz, 1H, OCH_2^{Bn}), 4.56 (dt, *J* = 8.3, 4.5 Hz, 1H, α-CH^{Phe}), 4.44–4.34 (m, 1H, α-CH^{Leu}), 3.02 (dd, *J* = 13.9, 4.6 Hz, 1H, β-CH_2^{Phe}), 2.77 (dd, *J* = 13.9, 8.3 Hz, 1H, β-CH_2^{Phe}), 1.72–1.50 (m, 3H, β-CH_2^{Leu}, γ-CH^{Leu}), 0.89 (d, *J* = 6.1 Hz, 3H, δ-CH_3^{Leu}), 0.84 (d, *J* = 6.0 Hz, 3H, δ-CH_3^{Leu}) ppm.

^{13}C-NMR, HMBC, HSQC (75 MHz, DMSO-d$_6$) δ = 172.2 (*C* = O^{Leu}), 171.6 (*C* = O^{Phe}), 154.3 (*C* = O^{Head}), 149.1 (d, *J* = 255.7 Hz, *C*-F), 137.3 (C_q^{Phe}), 137.1 (d, *J* = 2.8 Hz, *C*-1^{Head}), 136.4 (d, *J* = 8.2 Hz, *C*-3^{Head}), 135.9 (C_q^{Bn}), 129.4, 128.5, 128.1, 128.1, 127.9, 126.4 (CH^{Ar}), 124.7 (d, *J* = 7.7 Hz, *C*-6^{Head}), 118.6 (d, *J* = 21.9 Hz, *C*-5^{Head}), 113.4 (d, *J* = 3.5 Hz, *C*-2^{Head}), 66.1 (CH$_2^{Bn}$), 53.5 (α-CH^{Phe}), 50.4 (α-CH^{Leu}), 39.7 (β-CH$_2^{Leu}$), 38.3 (β-CH$_2^{Phe}$), 24.2 (γ-CH^{Leu}), 22.8, 21.3 (δ-CH$_3^{Leu}$) ppm.

IR: ν = 3348, 1730, 1664, 1645, 1529, 1480, 1345, 1260, 1222, 1135, 958, 890, 827, 753, 738, 699 cm^{-1}.

ESI-HRMS (*m*/*z*) calculated for $[C_{29}H_{31}N_4O_6NaF]^+$ = 573.2125, found 573.2111.

$[\alpha]_D^{24}$ = +13.4° (c = 1.0, DMSO).

1.10. Benzyl-N-(2-Fluoro-5-Nitrobenzoyl)-L-Phenylalanyl-L-Leucinate (6)

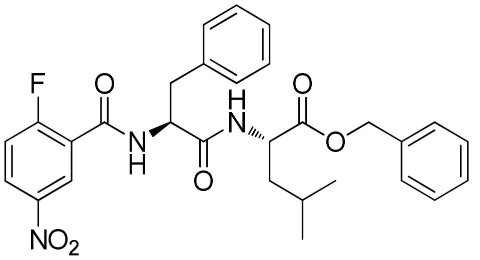

To a solution of 2-fluoro-5-nitrobenzoic acid (100 mg, 0.54 mmol) in toluene (5 mL) thionyl chloride (68 µL, 0.95 mmol) was added over a period of 15 minutes. The solution was heated to 80 °C for 12 h. After removal of the solvent, the product crystallized as a colorless solid (110 mg, 0.54 mmol, quant.). The product was used without further purification in the next step. The hydrochloride of the deprotected dipeptide **A2** (200 mg, 0.54 mmol) was dissolved in dichloromethane (3 mL) and triethylamine (75 µL, 0.54 mmol) was added. Then, a solution of acid chloride (110 mg, 0.54 mmol) in dichloromethane (2 mL) was added slowly. The reaction mixture was stirred at room temperature for 48 h before removing the solvent under reduced pressure. Purification by chromatography (SiO_2, petroleum ether:ethyl acetate (5:1)) afforded the title compound (142 mg, 0.27 mmol, 50 %) as a colorless solid.

R$_f$ = 0.65 (petroleum ether:ethyl acetate, 5:3).

mp: 163–166°C.

^{1}H-NMR, COSY (300 MHz, CDCl$_3$) δ = 8.92 (dd, *J* = 6.4, 3.0 Hz, 1H, *H*-6^{Head}), 8.36 (ddd, *J* = 9.0, 4.2, 3.0 Hz, 1H, *H*-4^{Head}), 7.45–7.21 (m, 12H, H^{Ar}, NH^{Phe}), 6.05 (d, *J* = 7.9 Hz, 1H, NH^{Leu}), 5.18 (d, *J* = 12.3 Hz, 1H, OCH_2^{Bn}), 5.14 (d, *J* = 12.3 Hz, 1H, OCH_2^{Bn}), 4.94–4.82 (m, 1H, α-CH^{Phe}), 4.62–4.52 (m, 1H, α-CH^{Leu}), 3.23 (dd, *J* = 13.8, 6.1 Hz, 1H, β-CH_2^{Phe}), 3.12 (dd, *J* = 13.8, 7.5 Hz, 1H, β-CH_2^{Phe}), 1.65–1.39 (m, 3H, β-CH_2^{Leu}, γ-CH^{Leu}), 0.85 (d, *J* = 5.8 Hz, 6H, δ-CH_3^{Leu}) ppm.

^{13}C-NMR, HMBC, HSQC (75 MHz, CDCl$_3$) δ = 172.1 (*C* = O^{Leu}), 170.0 (*C* = O^{Phe}), 160.8 (d, *J* = 3.4 Hz, *C* = O^{Head}) 144.8 (d, *J* = 2.2 Hz, *C*-5^{Head}), 136.1 (C_q^{Phe}), 135.4 (C_q^{Bn}), 129.5, 129.0, 128.9, 128.8, 128.7, 128.7, 128.5 (CH^{Ar}), 128.3 (d, *J* = 4.4 Hz *C*-6^{Head}), 127.5 (CH^{Ar}), 117.9 (d, *J* = 27.5 Hz, *C*-3^{Head}), 67.3 (CH$_2^{Bn}$), 55.4 (α-CH^{Phe}), 51.4 (α-CH^{Leu}), 41.5 (β-CH$_2^{Leu}$), 38.5 (β-CH$_2^{Phe}$), 24.9 (γ-CH^{Leu}), 22.8, 22.1 (δ-CH$_3^{Leu}$) ppm.*

IR: ν = 3263, 1735, 1649, 1547, 1529, 1389, 1347, 1331, 1276, 1241, 1188, 1140, 1082, 947, 911, 848, 790, 746, 698, 662 cm^{-1}.

ESI-HRMS (*m*/*z*) calculated for $[C_{29}H_{31}N_3O_6F]^+$ = 536.2197, found 536.2198.

$[\alpha]_D^{24}$ = –12.0° (c = 1.0, CH_2Cl_2).

*Some carbon Signals (*C*-1^{Head}, *C*-2^{Head}, *C*-4^{Head}) were not observed.

1.11. 1,2-Difluoro-4,5-dinitrobenzene (A4)

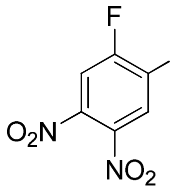

The product was synthesized according to Nitschke et al. [5]. To a mixture of sulfuric acid (6.4 mL) and nitric acid (15.9 mL) 1,2-difluorobenzene (2.00 g, 17.5 mmol) was slowly added at 0 °C. The resulting mixture was stirred 2 h at room temperature and 18 h at 100 °C. The solution was poured on ice and the precipitate was collected. Recrystallization from ethanol gave the title compound (812 mg, 4 mmol, 23 %) as a colorless solid.

R$_f$ = 0.50 (petroleum ether:ethyl acetate, 5:1).

mp: 82–84°C.

^{1}H-NMR, COSY (300 MHz, $CDCl_3$) δ = 7.85 (*app*-t, *J* = 7.5 Hz, 2H) ppm.

^{13}C-NMR, HMBC, HSQC (75 MHz, $CDCl_3$) δ = 152.0 (dd, J = 266.0, 14.9 Hz, *ipso*-*C*-1/2), 139.3 (br s, *C*-4/5), 115.8, 115.7, 115.6, 115.5, 115.4, 115.2 (m, *C*-3/6) ppm.

IR: ν = 3089, 1614, 1535, 1511, 1341, 1297, 1204, 1029, 892, 808, 790, 751, 693, 662 cm^{-1}.

FD-MS (*m*/*z*) = 204.0 (100 %, $[M]^+$).

1.12. Benzyl-N-(2-fluoro-4,5-dinitrophenyl)-L-phenylalanyl-L-leucinate (7)

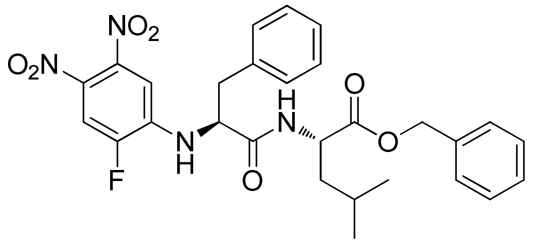

The hydrochloride of dipeptide **A2** (115 mg, 0.28 mmol) was dissolved in dichloromethane (5 mL) and triethylamine (39 µL, 0.28 mmol) was added. This solution was slowly added to a solution of 1,2-difluoro-4,5-dinitrobenzene (57 mg, 0.28 mmol) in dichloromethane (5 mL). The resulting mixture was stirred for 48 h at room temperature. Then the solvent was evaporated, the residue was taken up in ethyl acetate (20 mL) and washed with water, 0.5 M sodium bicarbonate and brine. Purification by chromatography (SiO_2, petroleum ether:ethyl acetate (5:1)) afforded the title compound (80 mg, 0.14 mmol, 52 %) as a light yellow solid. A byproduct (10 mg, 0.02 mmol, 7 %) was isolated in which the nitro group was substituted instead of the fluorine.

R$_f$ = 0.70 (petroleum ether:ethyl acetate, 5:3).

mp: 65–67°C.

^{1}H-NMR, COSY (300 MHz, $CDCl_3$) δ = 7.72 (d, *J* = 10.5 Hz, 1H, *H*-3^{Head}), 7.40–7.26 (m, 8H, *H*^{Ar}), 7.22–7.16 (m, 2H, *H*^{Ar}), 6.64 (d, *J* = 7.1 Hz, 1H, *H*-6^{Head}), 6.22 (d, *J* = 8.4 Hz, 1H, N*H*^{Leu}), 5.43 (dd, *J* = 6.4, 3.6 Hz, 1H, N*H*^{Phe}), 5.16 (d, *J* = 12.2 Hz, 1H, OC*H*$_2$^{Bn}), 5.11 (d, *J* = 12.2 Hz, 1H, OC*H*$_2$^{Bn}), 4.71–4.60 (m, 1H, α-C*H*^{Leu}), 4.19–4.10 (m, 1H, α-C*H*^{Phe}), 3.24 (dd, *J* = 14.0, 5.5 Hz, 1H, β-C*H*$_2$^{Phe}), 3.11 (dd, *J* = 14.0, 7.6 Hz, 1H, β-C*H*$_2$^{Phe}), 1.66–1.40 (m, 3H, β-C*H*$_2$^{Leu}, γ-C*H*^{Leu}), 0. 90 (d, *J* = 6.3 Hz, 3H, δ-C*H*$_3$^{Leu}), 0.89 (d, *J* = 6.2 Hz, 3H, δ-C*H*$_3$^{Leu}) ppm.

^{13}C-NMR, HMBC, HSQC (75 MHz, $CDCl_3$) δ = 172.2 (*C* = O^{Leu}), 169.6 (*C* = O^{Phe}), 149.4 (d, *J* = 250.7 Hz, *ipso*-*C*-2^{Head}) 142.7 (d, *J* = 2.9 Hz, *C*-5^{Head}), 140.5 (d, *J* = 12.7 Hz, *C*-1^{Head}), 135.2 (*C*$_q$^{Bn}), 135.1 (*C*$_q$^{Phe}), 130.3 (d, *J* = 7.8 Hz, *C*-4^{Head}), 129.4, 129.3, 128.8, 128.7, 128.5, 128.1 (*C*^{Ar}), 112.7 (d, *J* = 25.2 Hz, *C*-3^{Head}), 107.2 (d, *J* = 4.8 Hz *C*-6^{Head}), 67.6 (CH$_2$^{Bn}), 58.9 (α-CH^{Phe}), 51.3 (α-CH^{Leu}), 41.3 (β-CH$_2$^{Leu}), 39.3 (β-CH$_2$^{Phe}), 25.0 (γ-CH^{Leu}), 22.8, 21.9 (δ-CH$_3$^{Leu}) ppm.

IR: ν = 3065, 2959, 1734, 1655, 1619, 1546, 1456, 1379, 1326, 1195, 1149, 1108, 1031, 884, 842, 802, 748, 698, 658 cm^{-1}.

ESI-HRMS (*m*/*z*) calculated for $[C_{28}H_{29}N_4O_7NaF]^+$ = 575.1918, found 575.1918.
$[\alpha]_D^{24}$ = –13.7° (c = 1.0, CH_2Cl_2).

1.13. N-(tert-Butoxycarbonyl)-L-Phenylalanyl-L-Leucine

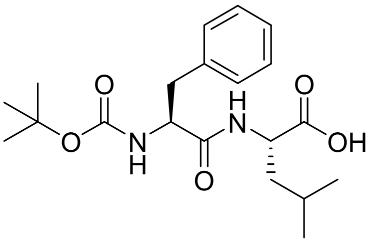

Benzyl *N*-(*tert*-butoxycarbonyl)-L-phenylalanyl-L-leucinate (120 mg, 0.26 mmol) was dissolved in THF (5 mL). Pd/C (20 mg) was added and the mixture was stirred under a hydrogen atmosphere for 2 h. The suspension was filtered over a pad of celite and washed with THF (15 mL). The solvent was evaporated under reduced pressure and the remaining colorless oil (89 mg, 0.26 mmol) was used without further purification.

ESI-MS: m/z = 2.9 min, 279.1 (100%, [M - Boc]$^+$, calc. 279.2).

1.14. L-Phenylalanyl-L-Leucine Trifluoroacetic Acid Salt

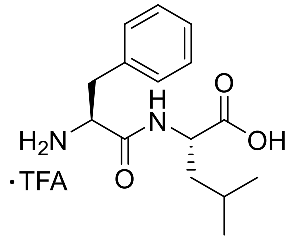

Crude *N*-(*tert*-butoxycarbonyl)- L-phenylalanyl-L-leucine (89 mg, 0.26 mmol) was dissolved in dichloromethane (2 mL) and TFA (1 mL) was added dropwise at room temperature. After stirring for 1 h, the solvents were removed in a stream of nitrogen. Remaining solvent was removed by co-evaporation with chloroform, toluene and ethanol (5 mL each) to yield a colorless solid (88 mg, 0.22 mmol).

ESI-MS: m/z = 0.7 min, 279.1 (100%, [M + H]$^+$, calc. 279.2).

1.15. N-(2-Fluoro-4,5-dinitrophenyl)-L-phenylalanyl-L-leucine (8)

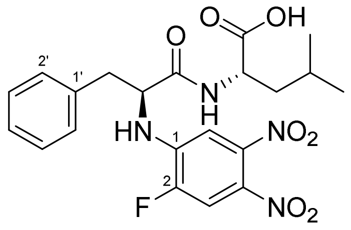

Crude L-phenylalanyl-L-leucine trifluoroacetic acid salt (23 mg, 0.081 mmol, 1 eq.), 1,2-difluoro-4,5-dinitrobenzene (25 mg, 0.122 mmol, 1.5 eq.) were dissolved in ethanol (3 mL) and 1 drop of triethylamine was added. The solution turned into light yellow and after 10 min a precipitate formed. After stirring for 17 h at room temperature, a significant amount of starting material was still present, so the suspension was heated to 80 °C for 21 h in a closed tube. After that time, all solids were dissolved and the solution had an intense yellow color. After evaporating the solvent, column chromatography (SiO_2, DCM:MeOH; 20:1 to 10:1) afforded a product mixture also containing the nitro substituted byproduct. Preparative HPLC (ACE C_{18}PFP, 5 μm, 150 mm × 30 mm, 37.5 mL/min, MeCN:water 1:1) gave the pure product (4 mg, 0.009 mmol, 11 %).

R$_f$ = 0.15 ($CHCl_3$:MeOH 10:1)

^{1}H-NMR, COSY (400 MHz, $CDCl_3$) δ = 7.68 (d, *J* = 10.4 Hz, 1H, *H*-3^{Head}), 7.38–7.26 (m, 3H, H^{Ar}), 7.24–7.17 (m, 2H, H^{Ar}), 6.86 (d, *J* = 7.0 Hz, 1H, *H*-6^{Head}), 6.59 (d, *J* = 8.2 Hz, 1H, NH^{Leu}), 5.30 (t, *J* = 4.6 Hz, 1H, NH^{Phe}), 4.61 (s, 1H, α-CH^{Leu}), 4.53–4.06 (m, 2H, O*H*, α-CH^{Phe}), 3.31 (dd, *J* = 14.1, 4.9 Hz, 1H, β-CH_2^{Phe}), 3.17 (dd, *J* = 14.1, 7.6 Hz, 1H, β-CH_2^{Phe}), 1.66 (m, 1H, γ-CH^{Leu}), 1.53 (m, 2H, β-CH_2^{Leu}), 0.94 (*app*-s, 3H, δ-CH_3^{Leu}), 0.93 (*app*-s, 3H, δ-CH_3^{Leu}) ppm.

^{13}C-NMR, HSQC, HMBC (101 MHz, $CDCl_3$): δ = 176.1 (COOH), 171.1 (*C* = O^{Phe}), 149.5 (d, *J* = 250.4 Hz, *C*-2^{Head}), 142.6 (d, *J* = 3.0 Hz, *C*-5^{Head}), 141.0 (d, *J* = 12.5 Hz, *C*-1^{Head}), 135.0 (*C*-1′), 130.3 (d, *J* = 7.7 Hz, *C*-4^{Head}), 129.4 (*C*-2′, *C*-6′), 129.2 (*C*-3′, *C*-5′), 128.1 (*C*-4′), 112.5 (d, *J* = 25.2 Hz, *C*-3^{Head}), 108.0 (d, *J* = 4.5 Hz, *C*-6^{Head}), 59.4 (α-CH^{Phe}), 51.2 (α-CH^{Leu}), 40.8 (β-CH_2^{Leu}), 39.0 (β-CH_2^{Phe}), 25.0 (γ-CH^{Leu}), 22.9, 21.7 (δ-CH_3^{Leu}) ppm.

IR: ν = 3380, 3086, 3031, 2960, 2872, 1720, 1656, 1619, 1546, 1469, 1326, 1240, 1213, 1154, 1110 cm^{-1}.

ESI-MS (*m*/*z*): 463.2 (100) [M + H]$^+$, 485.1 (23) [M + Na]$^+$.

ESI-HRMS (*m*/*z*) calculated for $[C_{21}H_{23}FN_4NaO_7]^+$ = 485.1443, found 485.1434.

$[\alpha]_D^{22}$ = -94.9 (c = 0.27, $CHCl_3$).

2. Analysis of the Reaction Products of the Reaction with LMW Thiol

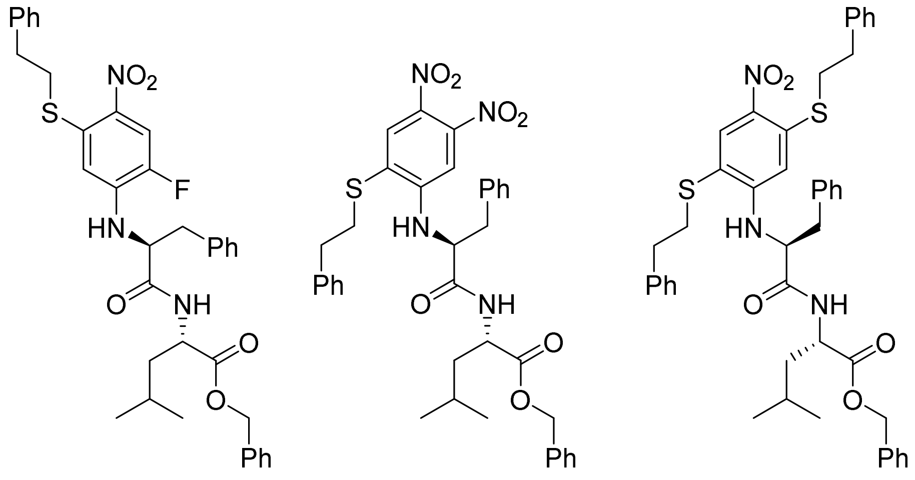

10 11 12

(10) (2*S*)-2-[Benzyl(*N*-{2-fluoro-4-nitro-4-[(2-phenylethyl)sulfanyl]phenyl}-L-phenylalanyl)amino]-4-methylpentanoate; (11) (2*S*)-2-[Benzyl(*N*-{4,5-dinitro-2-[(2-phenylethyl)sulfanyl]phenyl}-L-phenylalanyl)amino]-4-methylpentanoate; (12) (2*S*)-2-[Benzyl(*N*-{4-nitro-2,4-bis[(2-phenylethyl)sulfanyl]phenyl}-L-phenylalanyl)amino]-4-methylpentanoate.

Benzyl-*N*-(2-fluoro-4,5-dinitrophenyl)-L-phenylalanyl-L-leucinate (**7**, 47 mg, 85 μmol) was dissolved in ethanol (1 mL) and triethylamine (24 μL, 170 μmol) was added. While stirring phenylethanethiol (23 μL, 170 μmol) was added. After 28 h, starting material was still present. Another portion of base and thiol (170 μmol each) was added and the reaction mixture was stirred in a closed tube at 80 °C for 24 h. After evaporating the solvent, column chromatography (SiO_2, cyclohexane:ethyl acetate (5:1)) afforded a product mixture containing all three possible substitution products. Preparative HPLC (ACE C_{18}PFP, 5 μm, 150 mm × 30 mm, 37.5 mL/min, MeCN:water 7:3) gave the pure products as amorphous oils (8 mg, 12 μmol, 14 % (**10**); 2 mg, 3 μmol, 4 % (**11**); 21 mg, 28 μmol, 33 % (**12**)).

2.1. (2S)-2-[Benzyl(N-{2-fluoro-4-nitro-4-[(2-phenylethyl)sulfanyl]phenyl}-L-phenylalanyl)amino]-4-methylpentanoate (10)

R$_f$ = 0.27 (cyclohexane:ethyl acetate 10:3).

^{1}H-NMR, COSY (600 MHz, $CDCl_3$) δ = 7.95 (d, *J* = 11.4 Hz, 1H, *H*-3^{Head}), 7.35–7.17 (m, 15H, *H*^{Ar}), 6.42 (d, *J* = 7.7 Hz, 1H, *H*-6^{Head}), 6.40 (d, *J* = 8.4 Hz, 1H, N*H*^{Leu}), 4.99–4.97 (m, 1H, N*H*^{Phe}), 4.97 (s, 2H, OC*H*$_2$^{Bn}), 4.65–4.58 (m, 1H, *α*-C*H*^{Leu}), 4.09 (dt, *J* = 7.5, 5.2 Hz, 1H, *α*-C*H*^{Phe}), 3.26 (dd, *J* = 14.1, 5.3 Hz, 1H, β-C*H*$_2$^{Phe}), 3.19–3.15 (m, 1H, β-C*H*$_2$^{Phe}), 3.14–3.09 (m, 2H, CH_2C*H*$_2$S), 3.01–2.98 (m, 2H, C*H*$_2$$CH_2$S), 1.60–1.57 (m, 1H, β-C*H*$_2$^{Leu}), 1.50–1.47 (m, 1H, γ-C*H*^{Leu}), 1.46–1.43 (m, 1H, β-C*H*$_2$^{Leu}), 0.90 (d, *J* = 6.3, 3H, δ-C*H*$_3$^{Leu}), 0.87 (d, *J* = 6.4, 3H, δ-C*H*$_3$^{Leu}) ppm.

^{13}C-NMR, HSQC, HMBC (151 MHz, $CDCl_3$) δ = 172.2 (*C* = O^{Leu}), 170.8 (*C* = O^{Phe}), 147.1 (d, *J* = 241.9 Hz, *ipso*-*C*-2^{Head}), 140.3 (d, *J* = 12.0 Hz, *C*-1^{Head}), 139.7 (CH_2CH_2*C*^{Ar}), 138.0 (d, *J* = 2.2 Hz, *C*-5^{Head}), 135.3 (d, *J* = 7.0 Hz, *C*-4^{Head}), 135.3 (*C*$_q$^{Phe}), 135.2 (*C*$_q$^{Bn}), 129.4, 129.2, 128.8, 128.7, 128.7, 128.6, 128.2, 127.9, 126.9 (CH^{Ar}), 113.2 (d, *J* = 23.3 Hz, *C*-3^{Head}), 107.8 (d, *J* = 2.3 Hz, *C*-6^{Head}), 67.3 (CH$_2$^{Bn}), 59.4 (*α*-CH^{Phe}), 51.0 (*α*-CH^{Leu}), 41.1 (β-CH$_2$^{Leu}), 38.9 (β-CH$_2$^{Phe}), 34.0 (CH_2CH_2S), 33.6 (CH_2CH_2S), 24.9 (γ-CH^{Leu}), 22.9, 21.8 (δ-CH$_3$^{Leu}) ppm.

IR: ν = 3358, 2957, 1613, 1528, 1455, 1270, 1177, 1151, 1100, 733, 697 cm^{-1}.

ESI-MS (*m/z*): 644.4 (100) [M + H]$^+$, 666.3 (23) [M + Na]$^+$.

ESI-HRMS (*m/z*) calculated for $[C_{36}H_{39}FN_3O_5S]^+$ = 644.2589, found 644.2585.

$[\alpha]_D^{25}$ = –50.3° (c = 0.6, $CHCl_3$).

2.2. (2S)-2-[Benzyl(N-{4,5-dinitro-2-[(2-phenylethyl)sulfanyl]phenyl}-L-phenylalanyl)amino]-4-methylpentanoate (11)

R$_f$ = 0.26 (cyclohexane:ethyl acetate 10:3).

^{1}H-NMR, COSY (600 MHz, $CDCl_3$) δ = 7.98 (s, 1H, *H*-3^{Head}), 7.38–7.17 (m, 15H, *H*^{Ar}), 6.51 (s, 1H, *H*-6^{Head}), 6.23 (d, *J* = 5.4 Hz, 1H, N*H*^{Phe}), 6.20 (d, *J* = 8.5 Hz, 1H, N*H*^{Leu}), 5.10 (d, *J* = 12.2 Hz, 1H, OC*H*$_2$^{Bn}), 5.14 (d, *J* = 12.2 Hz, 1H, OC*H*$_2$^{Bn}), 4.68–4.63 (m, 1H, *α*-C*H*^{Leu}), 4.10 (dt, *J* = 8.0, 5.4 Hz, 1H, *α*-C*H*^{Phe}), 3.29 (dd, *J* = 14.1, 5.2 Hz, 1H, β-C*H*$_2$^{Phe}), 3.08 (dd, *J* = 14.2, 8.0 Hz, 1H, β-C*H*$_2$^{Phe}), 2.94 (dt, *J* = 12.7, 7.5 Hz, 1H, CH_2C*H*$_2$S), 2.86 (ddd, *J* = 12.7, 8.2, 6.5 Hz, 1H, CH_2C*H*$_2$S), 2.80–2.76 (m, 2H, C*H*$_2$$CH_2$S), 1.63–1.59 (m, 1H, β-C*H*$_2$^{Leu}), 1.52–1.48 (m, 1H, γ-C*H*^{Leu}), 1.48–1.42 (m, 1H, β-C*H*$_2$^{Leu}), 0.89 (d, *J* = 6.4, 3H, δ-C*H*$_3$^{Leu}), 0.87 (d, *J* = 6.4, 3H, δ-C*H*$_3$^{Leu}) ppm.

^{13}C-NMR, HSQC, HMBC (151 MHz, $CDCl_3$) δ = 172.2 (*C* = O^{Leu}), 169.9 (*C* = O^{Phe}), 151.3 (*C*-1^{Head}), 145.8 (*C*-5^{Head}), 138.8 (CH_2CH_2*C*^{Ar}), 135.3 (*C*$_q$^{Phe}), 135.2 (*C*$_q$^{Bn}), 131.7 (*C*-2^{Head}), 130.6 (*C*-3^{Head}), 129.4, 128.8, 128.7, 128.6, 128.5, 128.0, 127.1 (CH^{Ar}), 122.6 (*C*-2^{Head}), 105.3 (*C*-6^{Head}), 67.6 (CH$_2$^{Bn}), 59.6 (*α*-CH^{Phe}), 51.1 (*α*-CH^{Leu}), 41.3 (β-CH$_2$^{Leu}), 39.1 (β-CH$_2$^{Phe}), 36.3 (CH_2CH_2S), 35.8 (CH_2CH_2S), 25.0 (γ-CH^{Leu}), 22.8, 21.9 (δ-CH$_3$^{Leu}) ppm.

IR: ν = 3314, 2959, 2931, 1591, 1545, 1367, 1324, 1268, 1150, 699 cm^{-1}.

ESI-MS (*m/z*): 671.4 (77) [M + H]$^+$, 693.4 (53) [M + Na]$^+$.

ESI-HRMS (*m/z*) calculated for $[C_{36}H_{39}N_4O_7S]^+$ = 671.2534, found 671.2528.

$[\alpha]_D^{25}$ = –38.0° (c = 0.2, $CHCl_3$).

2.3. (2S)-2-[Benzyl(N-{4-nitro-2,4-bis[(2-phenylethyl)sulfanyl]phenyl}-L-phenylalanyl)amino]-4-methylpentanoate (12)

R$_f$ = 0.29 (cyclohexane:ethyl acetate 10:3).

^{1}H-NMR, COSY (600 MHz, $CDCl_3$) δ = 8.35 (s, 1H, *H*-3^{Head}), 7.34–7.15 (m, 18H, *H*^{Ar}), 7.06–7.03 (m, 2H, *H*^{Ar}), 6.39 (d, *J* = 8.3 Hz, 1H, N*H*^{Leu}), 6.33 (s, 1H, *H*-6^{Head}), 6.08 (d, *J* = 4.5 Hz, 1H, N*H*^{Phe}), 4.95 (s, 2H, OC*H*$_2$^{Bn}), 4.62–4.57 (m, 1H, *α*-C*H*^{Leu}), 4.08 (dt, *J* = 8.9, 4.7 Hz, 1H, *α*-C*H*^{Phe}), 3.33 (dd, *J* = 14.2, 4.7 Hz, 1H, β-C*H*$_2$^{Phe}), 3.21–3.13 (m, 2H, CH_2C*H*$_2$S), 3.08 (dd, *J* = 14.2, 8.4 Hz, 1H, β-C*H*$_2$^{Phe}), 3.03–2.99 (m, 2H, C*H*$_2$$CH_2$S), 2.75–2.56 (m, 4H, C*H*$_2$C*H*$_2$S), 1.57–1.53 (m, 1H, β-C*H*$_2$^{Leu}), 1.47–1.43 (m, 1H, γ-C*H*^{Leu}), 1.43–1.39 (m, 1H, β-C*H*$_2$^{Leu}), 0.87 (d, *J* = 6.4, 3H, δ-C*H*$_3$^{Leu}), 0.83 (d, *J* = 6.4, 3H, δ-C*H*$_3$^{Leu}) ppm.

^{13}C-NMR, HSQC, HMBC (151 MHz, $CDCl_3$) δ = 172.1 (*C* = O^{Leu}), 171.2 (*C* = O^{Phe}), 151.1 (*C*-1^{Head}), 143.2 (*C*-5^{Head}), 139.7 (CH_2CH_2*C*^{Ar}), 139.4 (CH_2CH_2*C*^{Ar}), 136.5(*C*-4^{Head}), 135.6 (C$_q$^{Phe}), 135.1 (*C*$_q$^{Bn}), 134.7 (*C*-3^{Head}), 129.3, 129.2, 128.8, 128.7, 128.7, 128.6, 128.6, 128.2, 127.8, 126.8, 126.8 (CH^{Ar}), 115.2 (*C*-2^{Head}), 106.2 (*C*-6^{Head}), 67.3 (CH$_2$^{Bn}), 60.2 (*α*-CH^{Phe}), 51.0 (*α*-CH^{Leu}), 41.1 (β-CH$_2$^{Leu}), 39.0 (β-CH$_2$^{Phe}), 36.9 (CH_2CH_2S), 35.9 (CH_2CH_2S), 33.9 (CH_2CH_2S), 33.3 (CH_2CH_2S), 25.0 (γ-CH^{Leu}), 22.8, 21.9 (δ-CH$_3$^{Leu}) ppm.

IR: ν = 3339, 3029, 2957, 2928, 1741, 1682, 1582, 1546, 1510, 1498, 1470, 1455, 1324, 1298, 1257, 1190, 1152, 1123, 752, 698 cm^{-1}.

ESI-MS (*m*/*z*): 762.6 (100) $[M + H]^+$, 784.4 (53) $[M + Na]^+$.

ESI-HRMS (*m*/*z*) calculated for $[C_{44}H_{48}N_3O_5S_2]^+$ = 762.3030, found 762.3023.

$[\alpha]_D^{25}$ = +1.0° (c = 0.5, $CHCl_3$).

3. Calculation of the Chemical Shifts / Determination of the Regiochemistry of the Addition Products

Conformational searches were performed using Spartan'10 [17]. DFT calculations were performed using Gaussian 16, Rev. A.03 [18]. All structures were confirmed as local minima by vibrational frequency analysis (N_{imag} = 0).

Relative Configuration Determination (DP4+): Conformational candidates were generated using a thorough or sparse systematic search algorithm at MMFF [19] and PM6[20] levels. The geometries were reoptimized and vibrational frequencies were calculated at the B3LYP/6-31G(d) level [21–27]. Duplicates were removed and conformers within a relative energy range of up to 4.5 kcal mol^{-1} were selected. NMR shielding tensors were calculated using GIAOs [28] at the mPW1PW91/6-31+G(d,p) [25–27,29] level with IEFPCM solvation [30] for chloroform. Boltzmann-weighted average shielding tensors were generated and compared with the experimental ^1H and ^{13}C NMR data in the DP4+ framework to obtain the DP4+ probabilities [31].

Input lines

Relative Configuration Determination (DP4+):

MMFF/PM6 Conformational Analysis:

SEARCHMETHOD = THOROUGH FINDBOATS KEEPALL CONF_SELECTION_RULE = 5 or

SEARCHMETHOD = SPARSE FINDBOATS KEEPALL CONF_SELECTION_RULE = 5

DFT Reoptimisation and Frequency Calculation:

#p opt = tight freq = noraman b3lyp 6-31g(d)

NMR Shielding Tensor Calculation

#p nmr = giao mpw1pw91 6-31+g(d,p) scrf = (iefpcm,solvent = chloroform)

Table S1 (DP4+)-results for product 12.

	5-nitro	*4-nitro*
*s*DP4+ (H data)	100.00%	0.00%
*s*DP4+ (C data)	0.00%	100.00%
*s*DP4+ (all)	100.00%	0.00%
*u*DP4+ (H data)	0.00%	100.00%
*u*DP4+ (C data)	0.00%	100.00%
*u*DP4+ (all)	0.00%	100.00%
DP4+ (H data)	100.00%	0.00%
DP4+ (C data)	0.00%	100.00%
DP4+ (all data)	0.00%	100.00%

4. Mass Spectrometry

Lyophilized rhodesain was reconstituted at 4 mg/mL in 50 mM Na acetate, pH 5.5, 200 mM NaCl, 5 mM EDTA. For mass spectrometric analysis, the protein was further diluted in the same buffer (to a final concentration of 850 nM) and reduced with DTT for 1 h at room temperature. After the addition of the inhibitors at a final concentration of 0.1 mM, samples were analyzed by LC-MS using a nanoAcquity UPLC system (Waters Corporation) coupled to a nano-ESI-Q-TOF mass spectrometer (Synapt G2-S HDMS, Waters Corporation). Rhodesain without compound served as control. Protein-drug complexes were loaded onto a 200 µm x 5 cm PepSwift Monolithic PS-DVB column from Dionex (Thermo Scientific) using direct injection mode. For LC separation, two mobile phases were used. Mobile phase A contained 0.1% formic acid (FA) and 3% DMSO in ultrapure water, whereas mobile phase B consisted of 0.1% FA and 3% DMSO in ACN. A gradient of 10-90% mobile phase B was run over 7 minutes at a flow rate of 2000 nL/min. Column temperature was set to 45 °C. After separation, the column was rinsed with 90% of mobile phase B and reequilibrated at initial conditions. All MS analyses were conducted in positive-mode ESI.

5. NMR Materials and Methods

For ^{19}F NMR, compounds **7** (800 µM) or **8** (400 µM) were dissolved in rhodesain sample buffer (50 mM NaAcetate, pH 5.5, 200 mM NaCl, 5 mM Na-EDTA, 1 mM TCEP) with 10% d_6-DMSO from a 40 mM inhibitor stock in d_6-DMSO. Spectra were recorded at 303 K on a Bruker Avance 3 600 MHz spectrometer with a Prodigy TCI cryoprobe (Bruker, Karlsruhe). Because ^{1}H and ^{19}F are measured in the same coil, protons were not decoupled during ^{19}F acquisition. Spectra were recorded with 512 scans (cpd **8**) or with 3k scans (cdp **7**) due to solubility issues. Unlabeled rhodesain was purified from *P. pastoris* as described previously and lyophilized until needed (see below). For NMR measurements, the lyophilized protein was resuspended in rhodesain buffer sample. For the time trace experiments, 2.1 nmol rhodesain (4 µM final conc.) was added to a NMR sample containing 800 µM of cpd. **7** and spectra recorded every 2 minutes. For observing the bound inhibitor, spectra of 400 µM cpd. **8** with 150 or 450 µM rhodesain were recorded (3k scans).

6. Enzyme and hydrolysis assays

6.1. Expression of Rhodesain:

Rhodesain with a S172A mutation and without the C-terminal domain beginning at Thr343 was expressed and purified from *Pichia pastoris* as described previously [7,8].

6.2. Hydrolysis Assays:

Rhodesain and rhodesain inactivated with K11777 were dissolved in 99 µL assay buffer (50 mM NaOAc, pH 5.5, 200 mM, 5 mM EDTA, 5 mM DTT) to a final concentration of 0.4 mg/mL. Afterwards, 1 µL of 10 mg/mL cpd. **7** dissolved in DMSO were added and the mixture was incubated at room temperature for 24 h. As a negative control, 1 µL cpd. **7** was added to 99 µL assay buffer without any protease. The reaction was quenched by addition of 100 µL ACN and heated up to 95 °C for 10 min. The solution was centrifuged for 10 min at 17,000 *g* and the supernatant was filtrated through a 0.22 µm syringe filter prior to LC-MS analysis.

The LC-MS analysis of the reaction mixtures was performed at an HP Agilent 1100 system coupled to an Agilent LC/MSD ion trap. An Agilent Poroshell 120 EC-C18 (150 x 2.10 mm) column at 40°C was used for separation with an isocratic flow of 30% ACN and 70% H_2O with 0.1% formic acid. The method showed a dead time of 0.3 min at a flow rate of 0.7 mL/min.

Unconverted cpd. **7** and hydrolysis product **8** were eluted as reference compounds as a mixture of pure inhibitor samples (t(**8**) = 0.9 min; t(**7**) = 2.1 min). The found masses fitted to the theoretical masses: [**7**+H^+] = 553.3 *m*/*z*, [**7**+Na^+] = 575.4 *m*/*z*, [**8**+H^+] = 463.3 *m*/*z*, [**8**+Na^+] = 485.3 *m*/*z*. A complete conversion of ester **7** to acid **8** was observed in the case of catalytically active rhodesain. A control proved that the ester **7** was not hydrolyzed by the reaction buffer. Furthermore, no conversion was observed for rhodesain irreversibly inactivated by K11777. For the latter experiment, 40.2 µM rhodesain was incubated in a mixture of 8.7 mM K11777 with 5% DMSO for 2 h at room temperature until no activity was observed in the substrate assay. Insoluble inhibitor was removed by centrifugation and the supernatant was used for the hydrolysis assay as described above.

6.3. Enzyme assays / Rhodesain and Cathepsins

The assays were performed as described previously [7,8] by incubating rhodesain in 190 µL assay buffer (50 mM sodium acetate pH 5.5, 5 mM EDTA, 200 mM NaCl, 0.005% Brij) with 5 µL of an inhibitor DMSO stock solution of a given concentration for 10 min at room temperature. The reaction was started by adding 5 µL of 400 µM Z-Phe-Arg-7-amino-4-methylcumarin (Z-Phe-Arg-AMC) in DMSO. The released AMC was excited at 380 nm and the upcoming fluorescence measured at 460 nm at 25 °C over time. The final substrate concentration was 10 µM.

Quenching of the fluorescence of the released AMC by the inhibitors was accounted for as described previously [9].

The cathepsin L assays were performed accordingly in 50 mM Tris buffer (pH 6.5, 5 mM EDTA, 200 mM NaCl, 0.005% Brij) and 250 µM Z-Phe-Arg-AMC concentration (final concentration: 6.25 µM).

The cathepsin B (Calbiochem) assays were performed accordingly in 50 mM Tris buffer pH 6.5, 5 mM EDTA, 200 mM NaCl and 0.005% Brij leading to final enzyme and substrate concentrations of 484 nM and 100 µM.

Final inhibitor concentrations ranged from 0.001 µM to 100.0 µM.

6.4. Enzyme Assays / Sortase A

The sortase A assays were performed as described previously [32] by incubating the recombinantly expressed *S. aureus* SrtA (1 µM) with 25 µM of the FRET-pair substrate Abz-LPETG-Dap(Dnp)-OH and 0.5 mM tetraglycine in assay buffer (50 mM Tris, 150 mM NaCl, 5 mM $CaCl_2$, pH 7.50). Reactions were initiated by addition of SrtA and monitored for 30 min at 25 °C with λ_{ex} 320 nm/λ_{em} 430 nm.

6.5. *Enzyme Assays / DENV PR*

The assays were performed as described previously [32].

6.6. *Dilution Assays:*

Dilution assays were performed by adding 50 µL of cpd. **8** (0.1 mM in DMSO) to rhodesain (0.37 mM final concentration) in 950 µL assay buffer (50 mM sodium acetate pH 5.5, 5 mM EDTA, 200 mM NaCl) followed by 45 min incubation at room temperature. In a control, the cpd. **8** solution was replaced by 50 µL pure DMSO and treated equally. 5 µL of 400 mM Z-Phe-Arg-AMC were added to 195 µL of rhodesain-**8** or rhodesain-DMSO stock solutions and the upcoming fluorescence was measured over time to assure complete inhibition (see Figure SI1)).

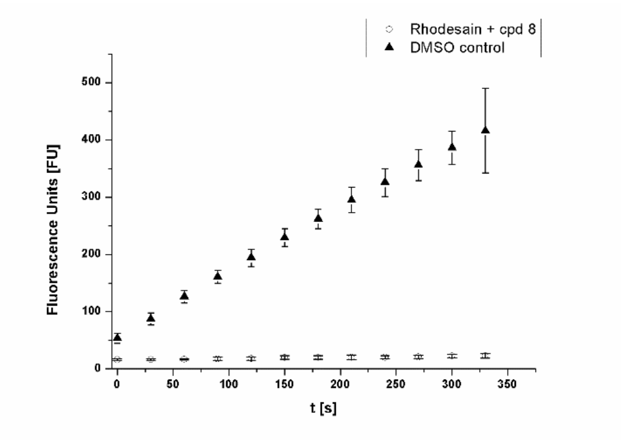

Figure SI1: Progress curves for substrate hydrolysis by rhodesain incubated with inhibitor 8 (°) or DMSO (▲).

For the dilution assay, 2 mL of the rhodesain-**8** stock solution were diluted with 193 mL 50 mM sodium acetate buffer (pH 5.5 with 5 mM EDTA, 200 mM NaCl 5 mM DTT) and the recovery of the enzymatic activity was measured immediately after adding 5 mL of 400 µM Z-Phe-Arg-AMC (see Figure SI2).

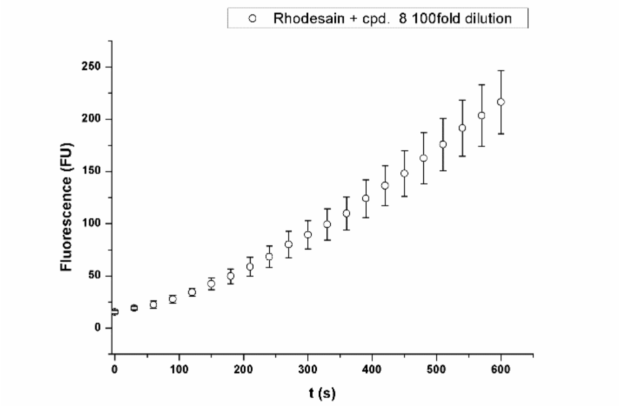

Figure S2: Progress curve for substrate hydrolysis by rhodesain, completely inhibited with inhibitor 8, after dilution. The recovery of the enzymatic activity proves the reversibility of the inhibition.

7. *T. b. brucei* Cell Survival Assay

Toxicity of **7** and **8** against trypanosomes (*T. b. brucei* 449 cell line) were determined via an ATPlite assay as described previously [10–12] using the cellular ATP levels as a proxy for cell viability. **7** (5 mM stock solution in DMSO) and **8** (50 mM stock solution in DMSO) were first diluted

1:3 in medium, followed by a 1:10 dilution step in a microplate and ten subsequent 1:2 dilution steps. 90 µL HMI-9 medium containing 2500 cells/mL were distributed in 96-well microplates (PerkinElmer). 10 µL of the 1:2 dilution step preparations of the tested compounds were added to the 90 µL cell suspension leading to final concentrations from 16.67 µM to 32.55 nM for **7** and 166.67 µM to 325.52 nM for **8** in the microplates. As a negative control, addition of 0.3% of DMSO corresponding to the highest DMSO concentration added by compound application was used. 10% DMSO was used as a positive control, since at this DMSO concentration, all cells die. Measurements were carried out as two sets of triplicates incubated at 37 °C for 24 h and 48 h. 50 µL of ATPlite 1 step solution (PerkinElmer) was added to each well of the microplate and luminescence measured at room temperature with an Infinite® M200 PRO plate reader (Tecan Trading AG). The measured values were plotted against the compound concentrations top yield the dose-response curve. The EC_{50} values were calculated using GraFit version 5.013 (Erithacus Software Ltd.).

8. Docking Procedures

Docking experiments were performed using the crystal structure of rhodesain bound to the covalent inhibitor K11777 (pdb 2p7u) [13]. To define the binding site, all residues within a 6.5 Å shell around K11777 were selected. All water molecules present in the crystal structure were omitted. Generation of 3D-coordinates and energy minimization of the ligands were accomplished with the Molecular Operating Environment (MOE) using the MMFF94x force field [14]. Docking calculations were run with FlexX (version 2.3.2) and ranked with the built-in empirical scoring function.[15] Selected docking solutions were visualized with PyMOL (version 2.3.0) [16].

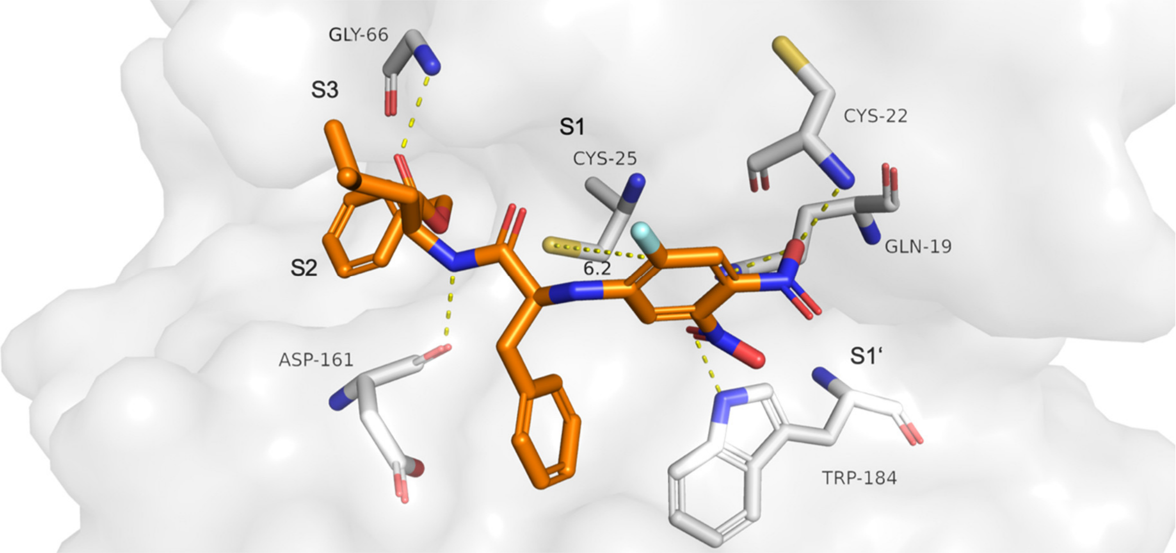

Figure S3. Highest ranked docking solution of ester **7** (score –20.56). The substituted aromatic ring binds to the S1′ region and is not located close to the nucleophilic cysteine (distance 6.2 Å). Light grey: solvent accessible surface of rhodesain, grey: carbon atoms of rhodesain amino acid residues, orange: carbon atoms of ester **7**, blue: nitrogen, red: oxygen, yellow: sulfur, cyan: fluorine.

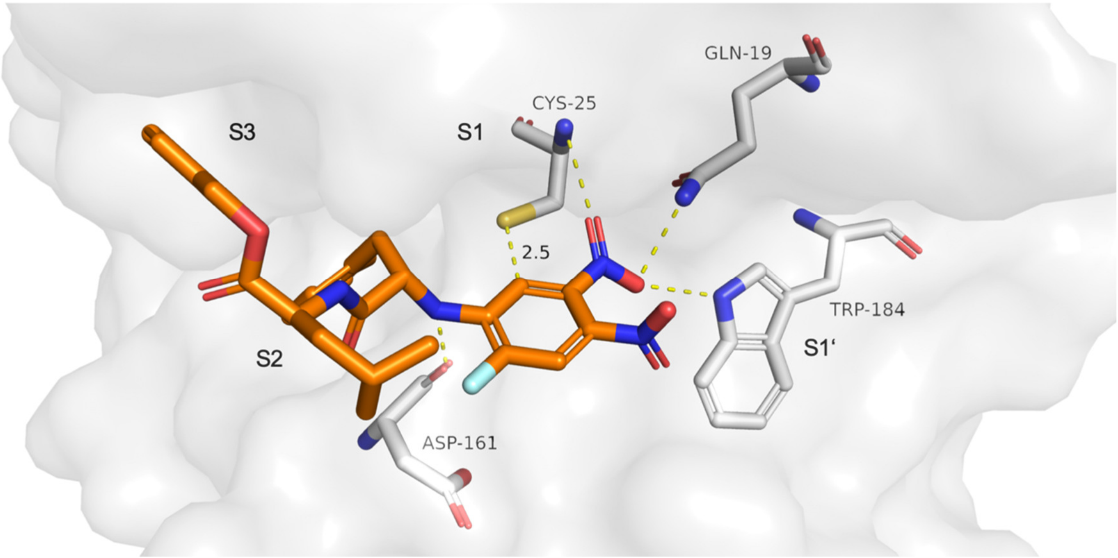

Figure S4: Docking solution of ester 7 with similar binding mode compared to acid 8 (score –18.26). The substituted aromatic ring is located in close proximity to the nucleophilic cysteine (2.5 Å). One nitro group forms hydrogen bonds to Gln-19 and Trp-184. The phenylalanine side chain is placed in the S2 pocket and the benzyl ester group occupies the S3 pocket. Light grey: solvent accessible surface of rhodesain, grey: carbon atoms of rhodesain amino acid residues, orange: carbon atoms of ester **7**, blue: nitrogen, red: oxygen, yellow: sulfur, cyan: fluorine.

9. NMR Spectra of the Compounds

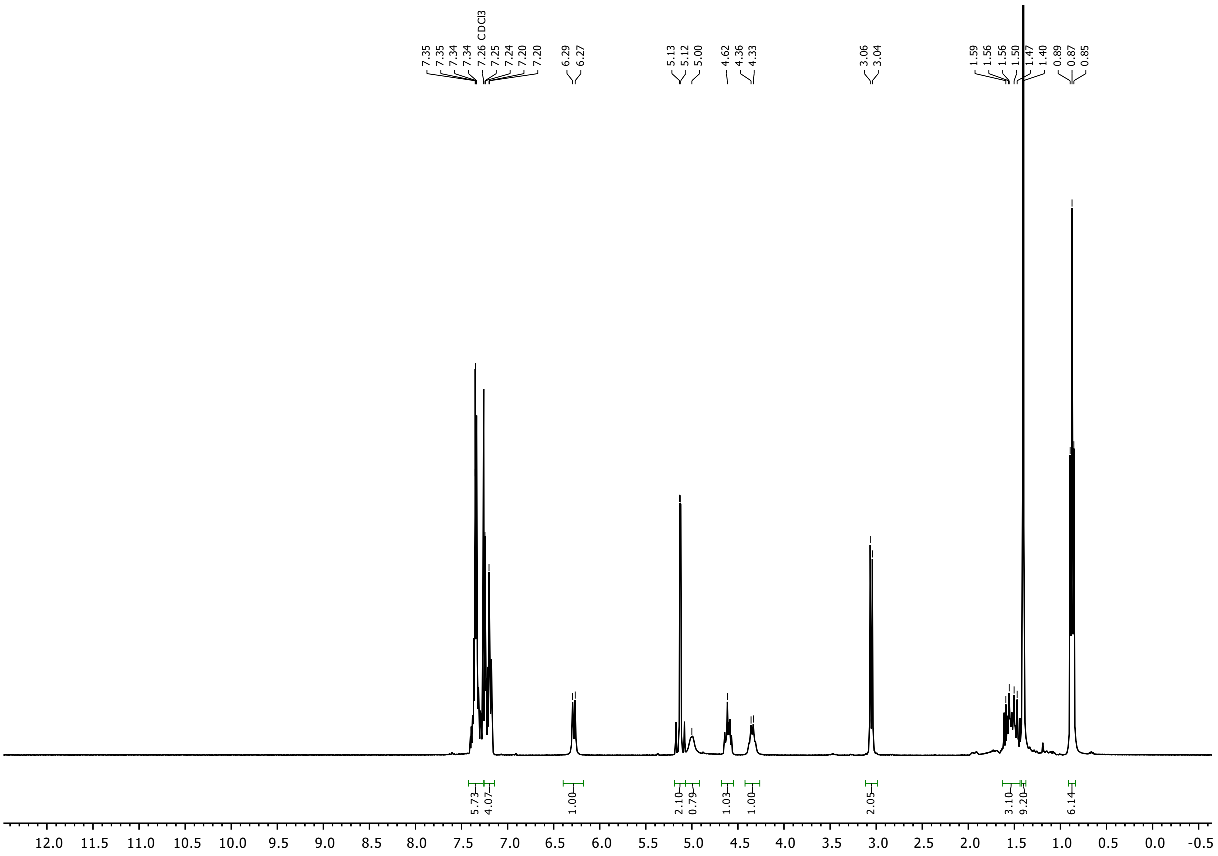

¹H-NMR (300 MHz, $CDCl_3$) spectrum of A1.

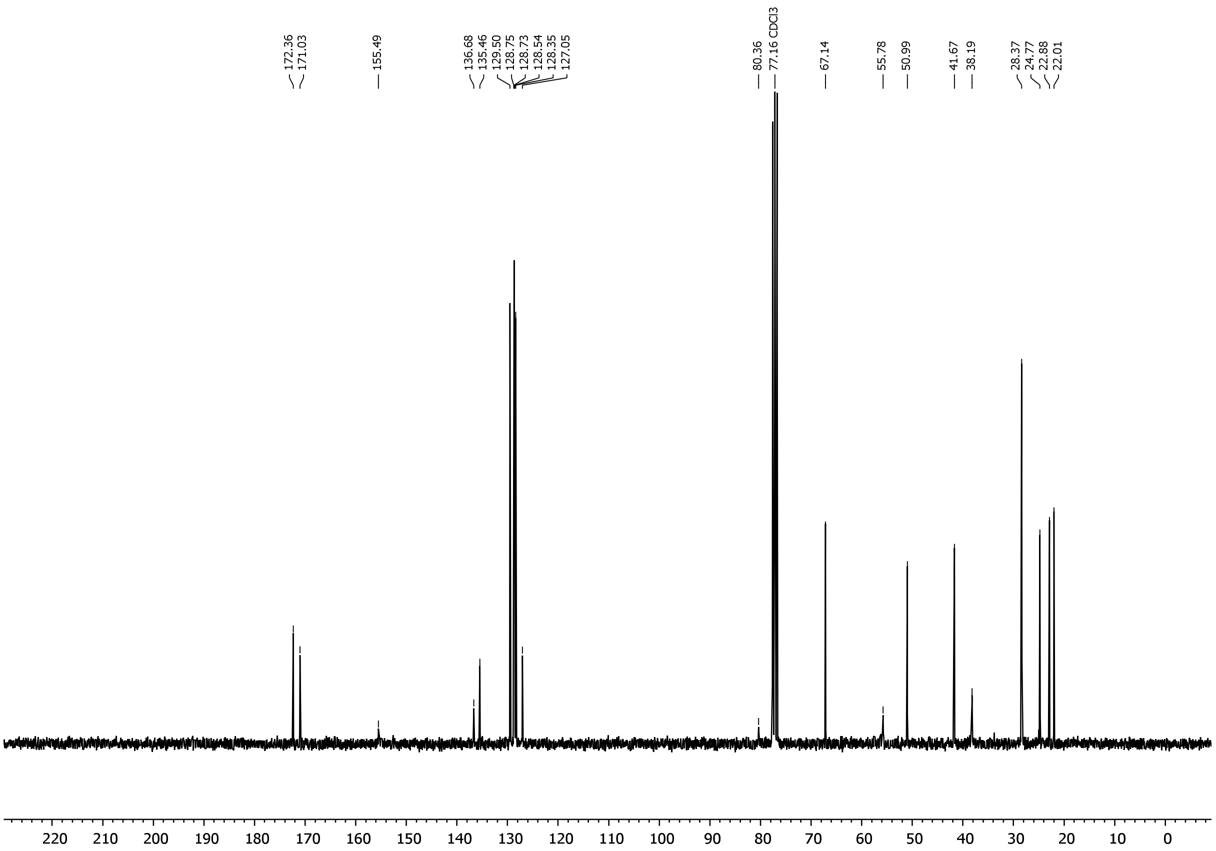

^{13}C-NMR (75 MHz, $CDCl_3$) spectrum of A1.

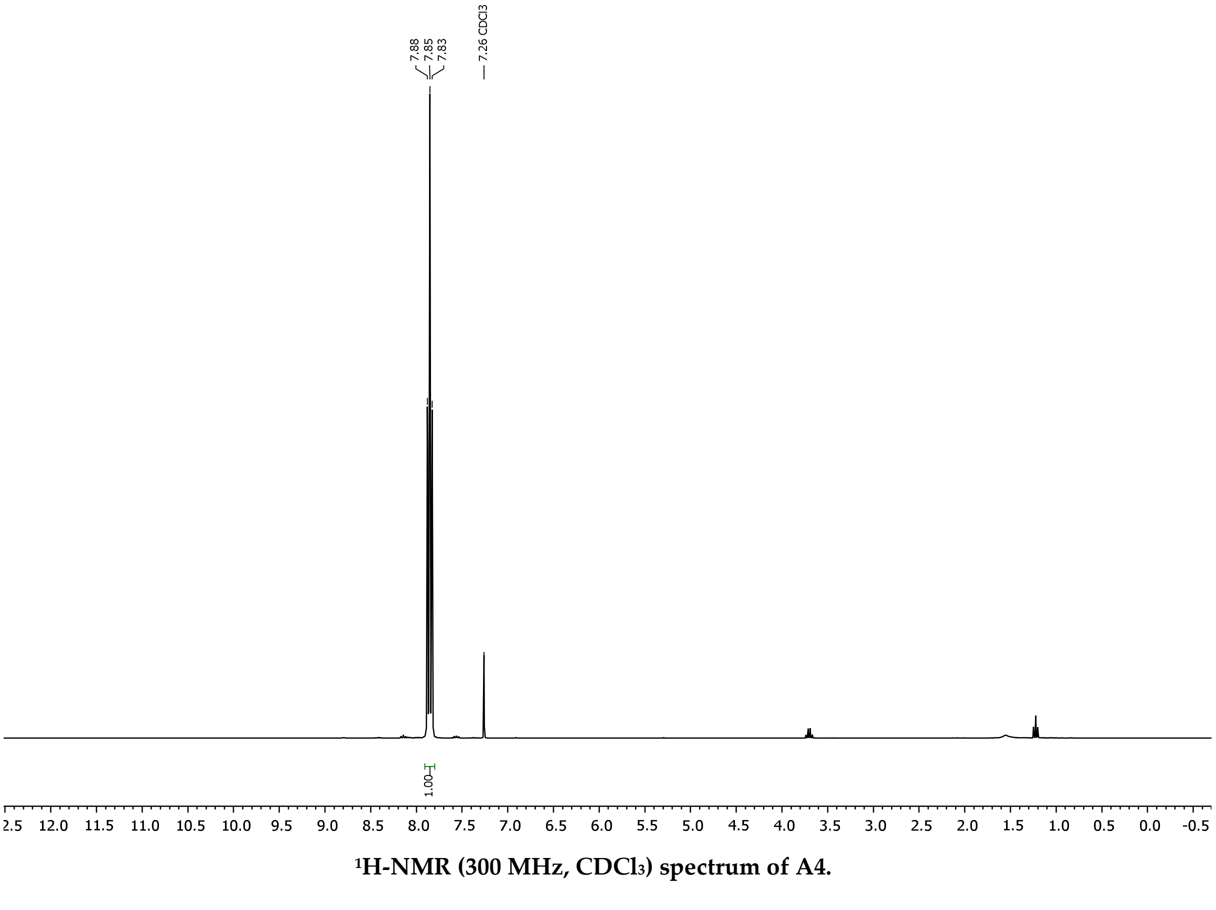

^{1}H-NMR (300 MHz, $CDCl_3$) spectrum of A4.

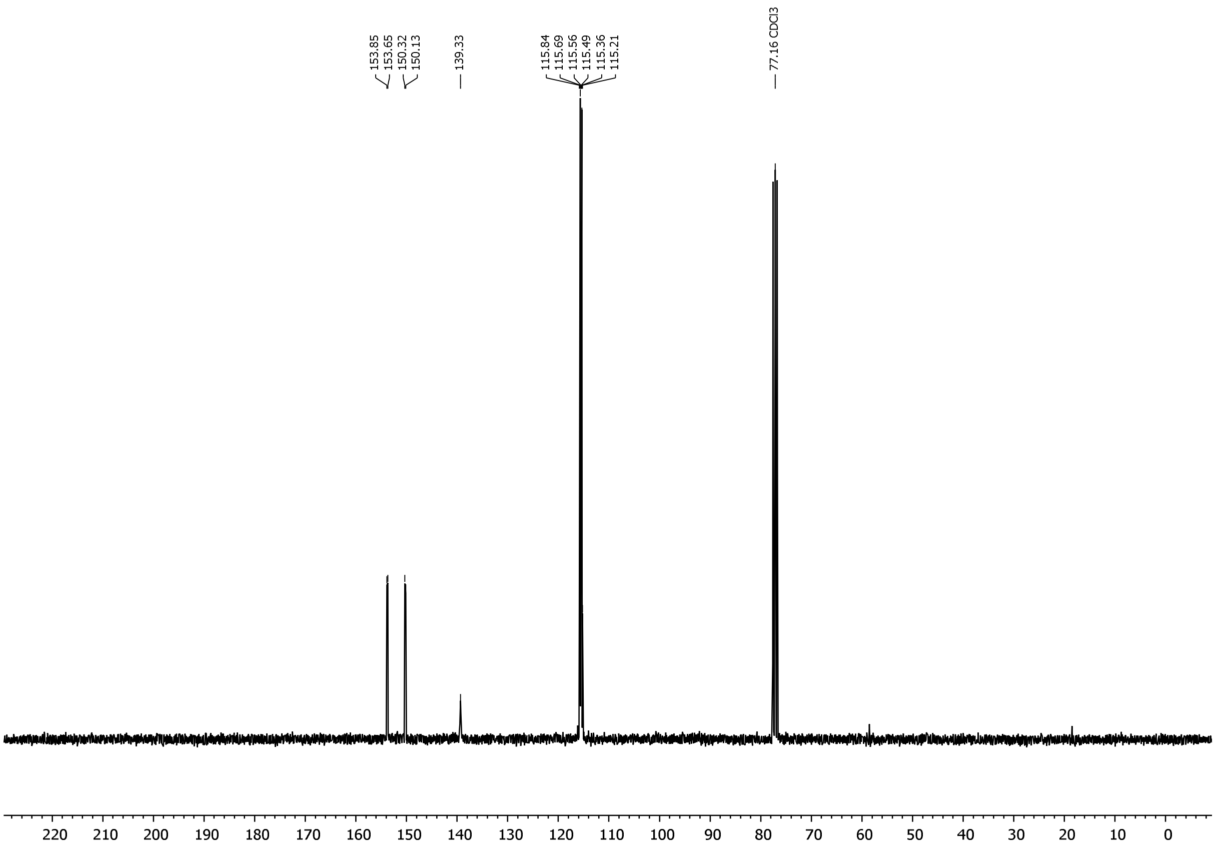

^{13}C-NMR (75 MHz, $CDCl_3$) spectrum of A4.

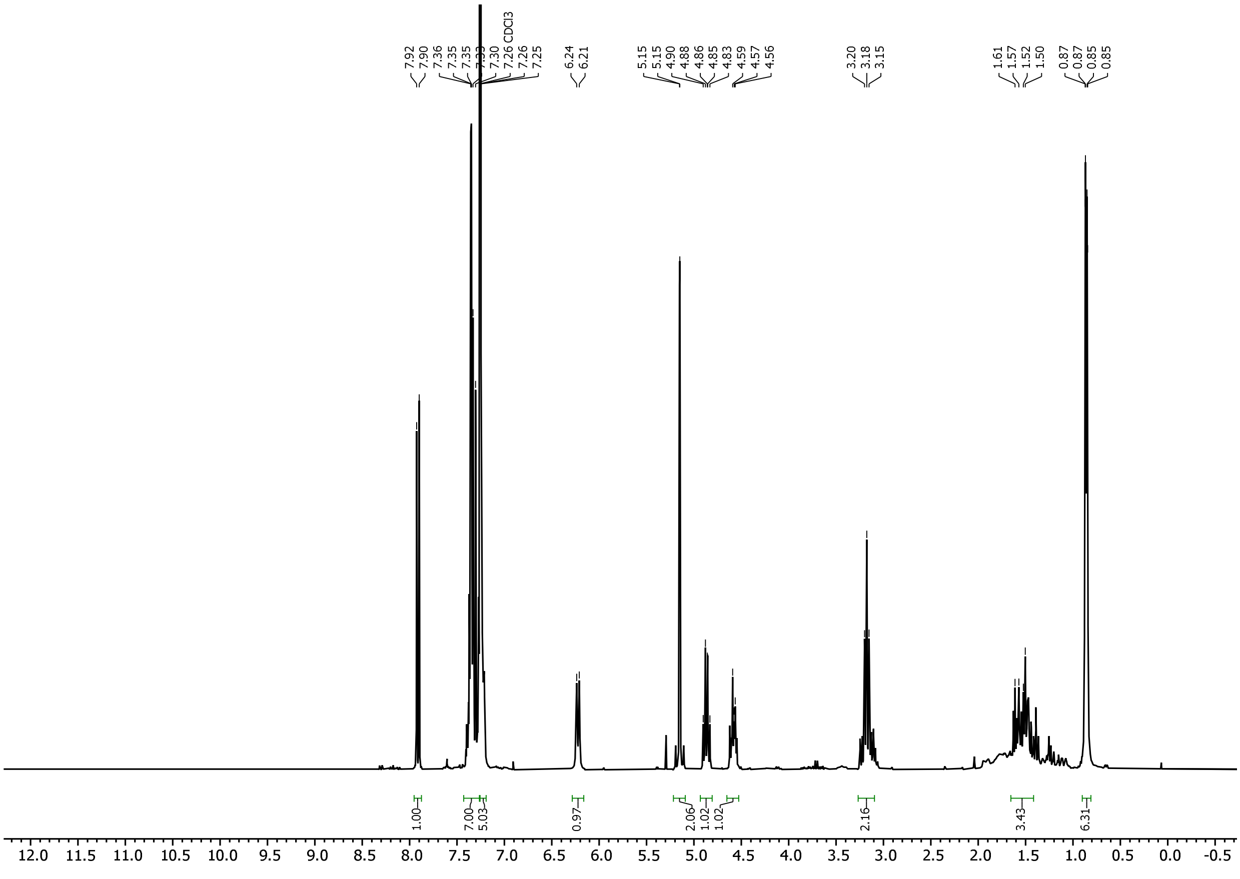

^{1}H-NMR (300 MHz, $CDCl_3$) spectrum of 1.

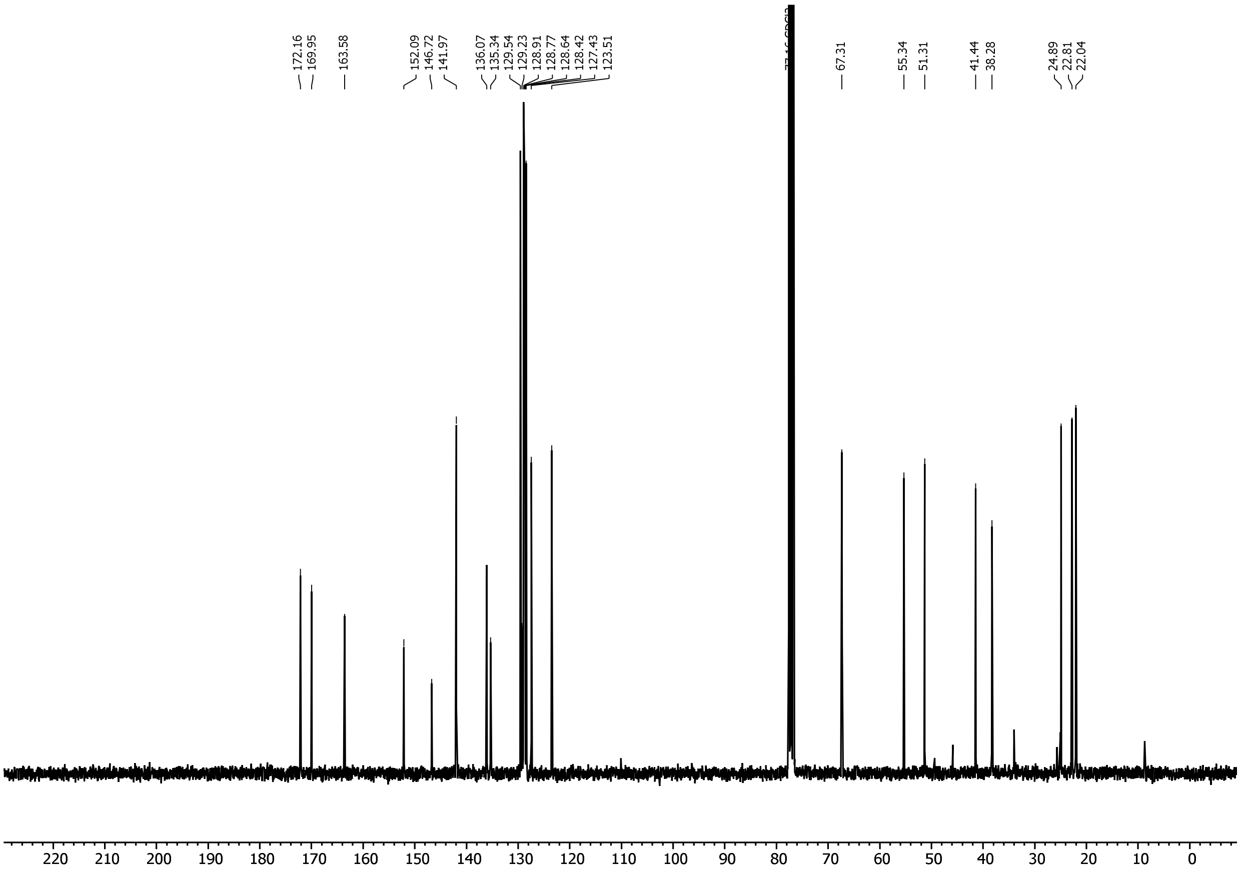

^{13}C-NMR (75 MHz, CDCl$_3$) spectrum of 1.

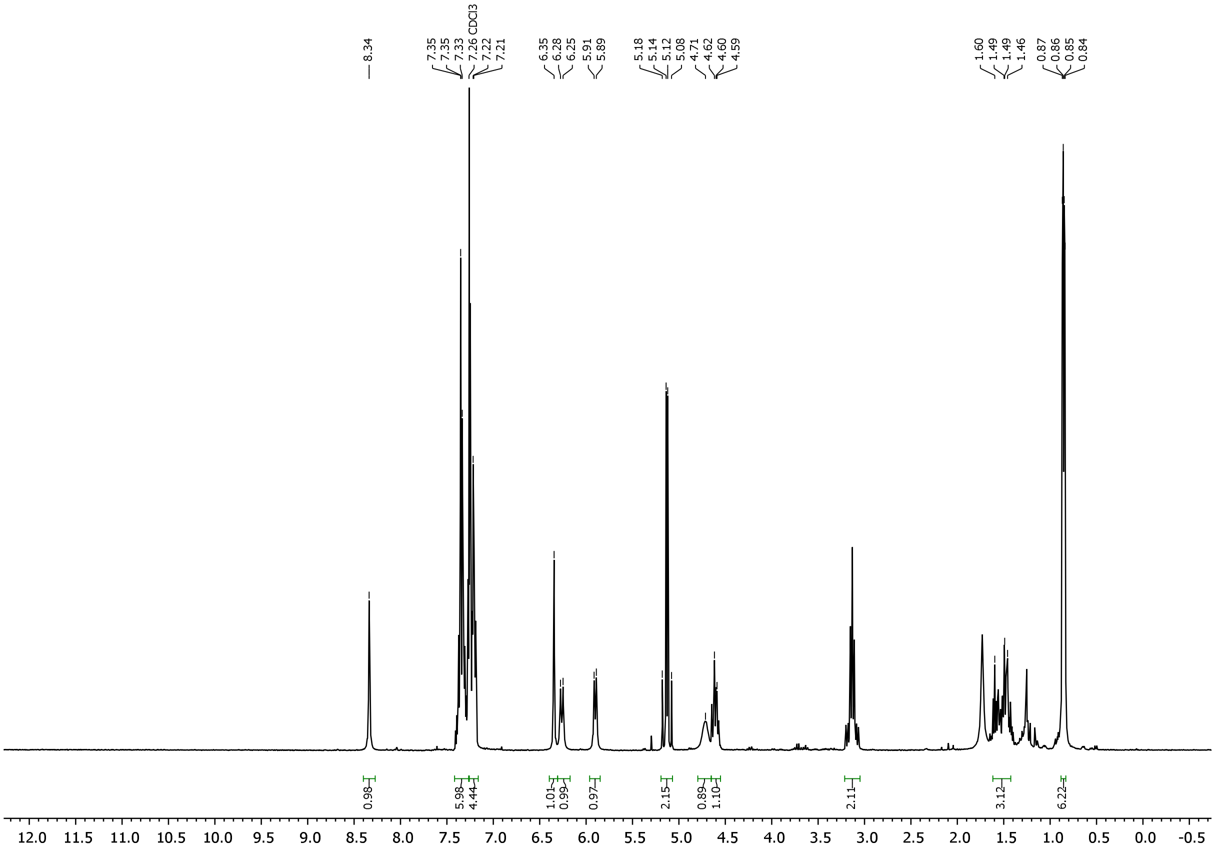

^{1}H-NMR (300 MHz, CDCl$_3$) spectrum of 2.

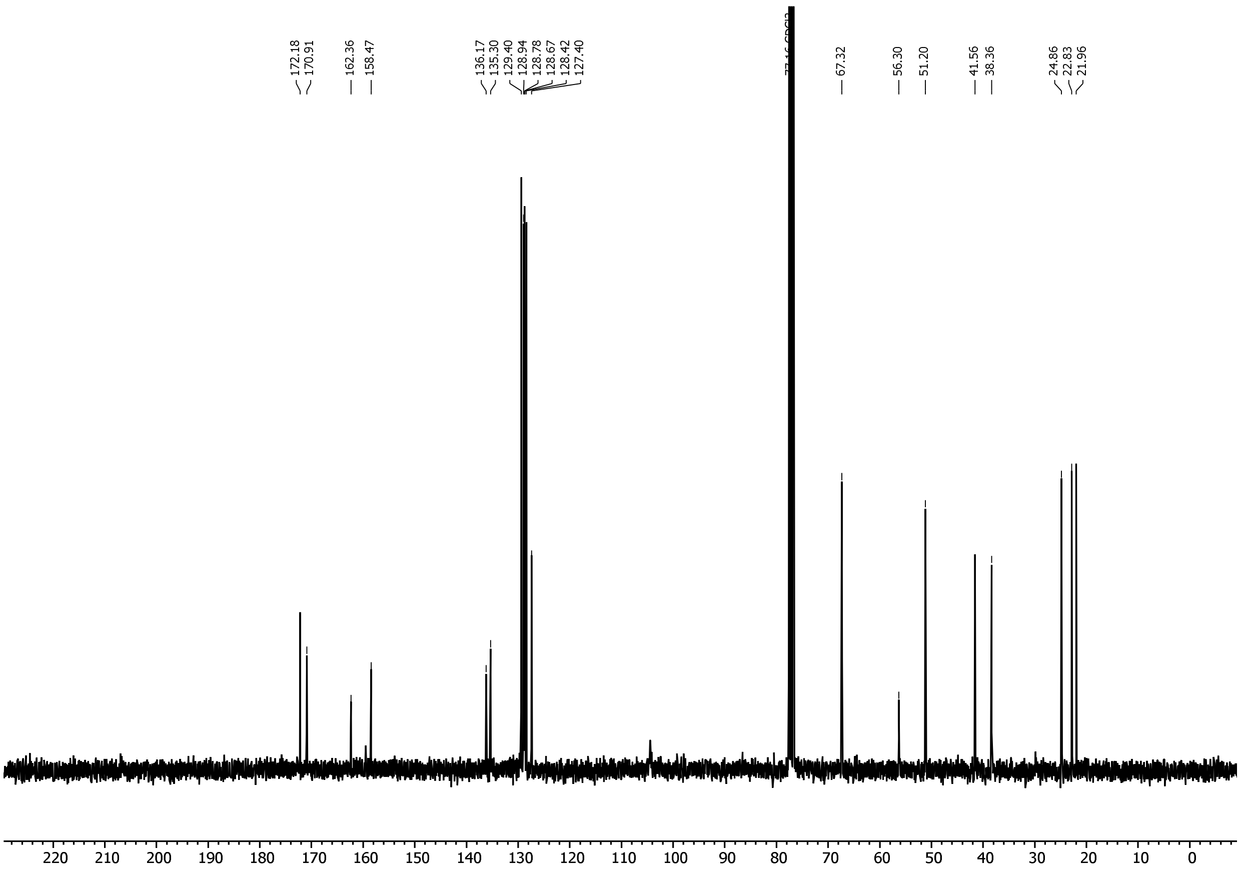

^{13}C-NMR (75 MHz, $CDCl_3$) spectrum of 2.

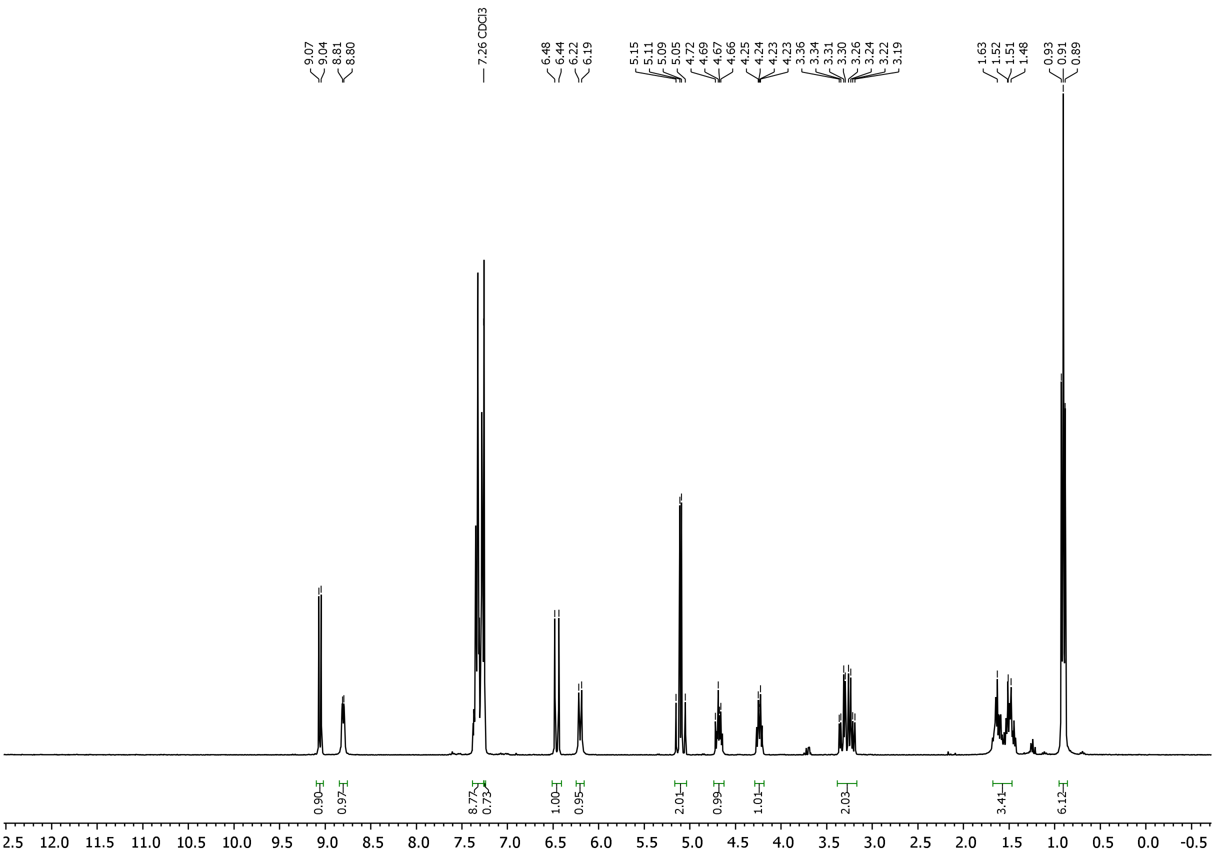

^{1}H-NMR (300 MHz, $CDCl_3$) spectrum of 3.

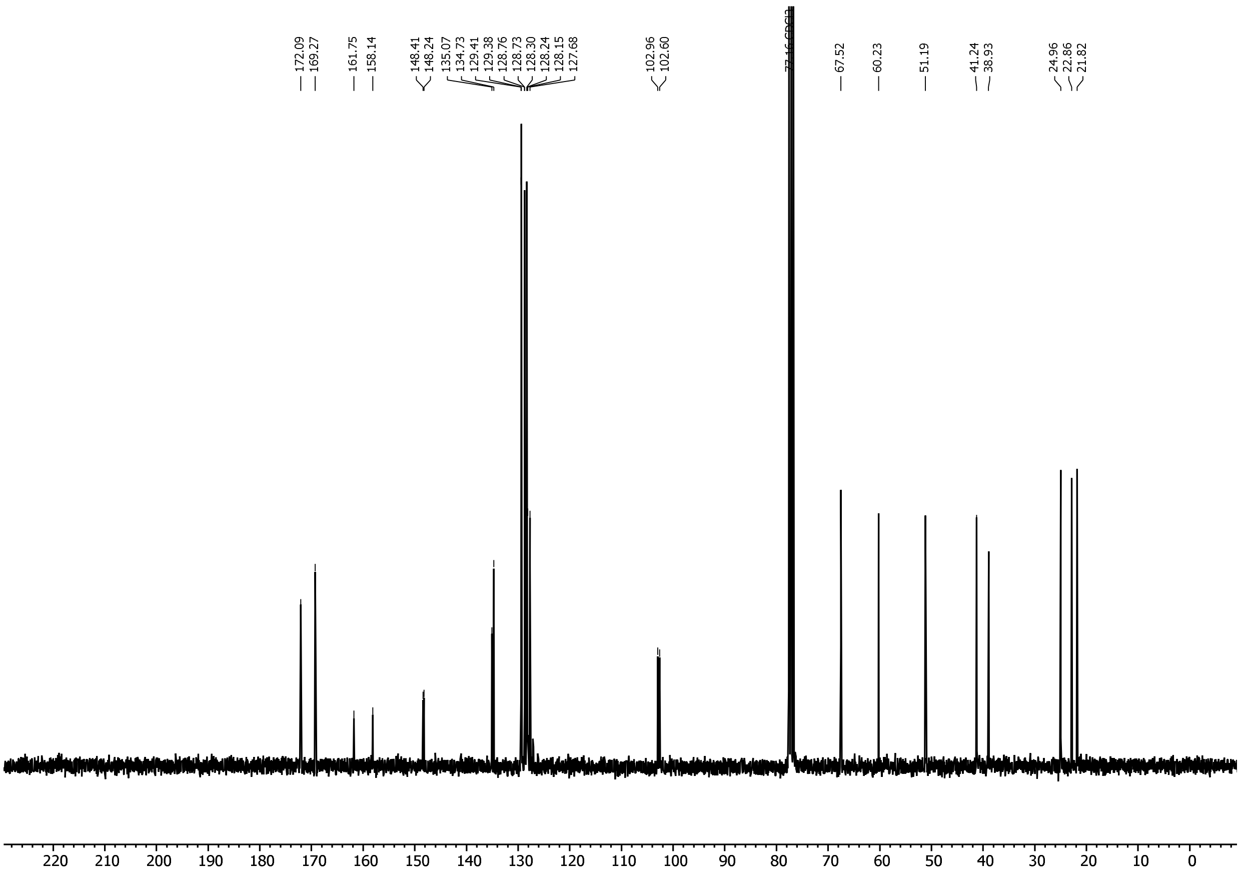

^{13}C-NMR (75 MHz, $CDCl_3$) spectrum of 3.

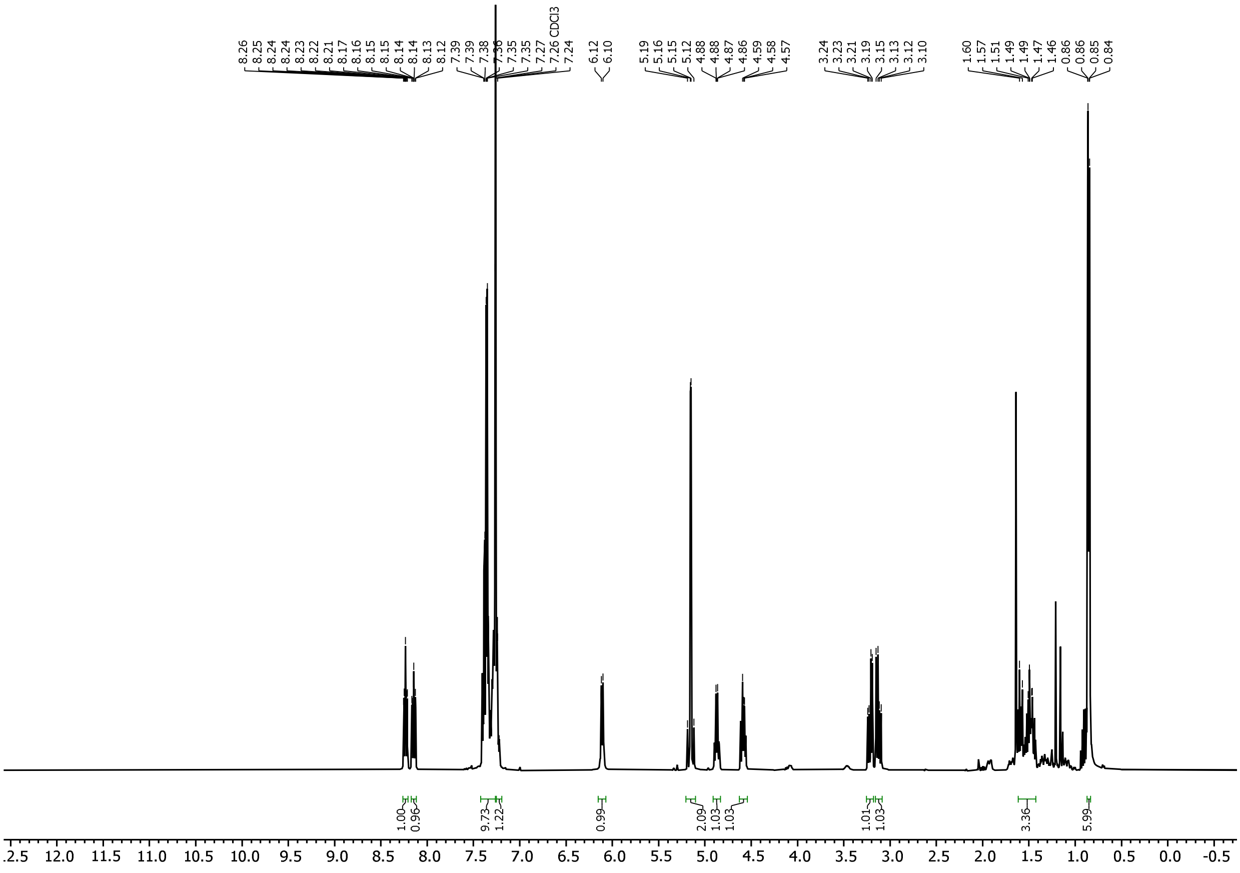

^{1}H-NMR (400 MHz, $CDCl_3$) spectrum of 4.

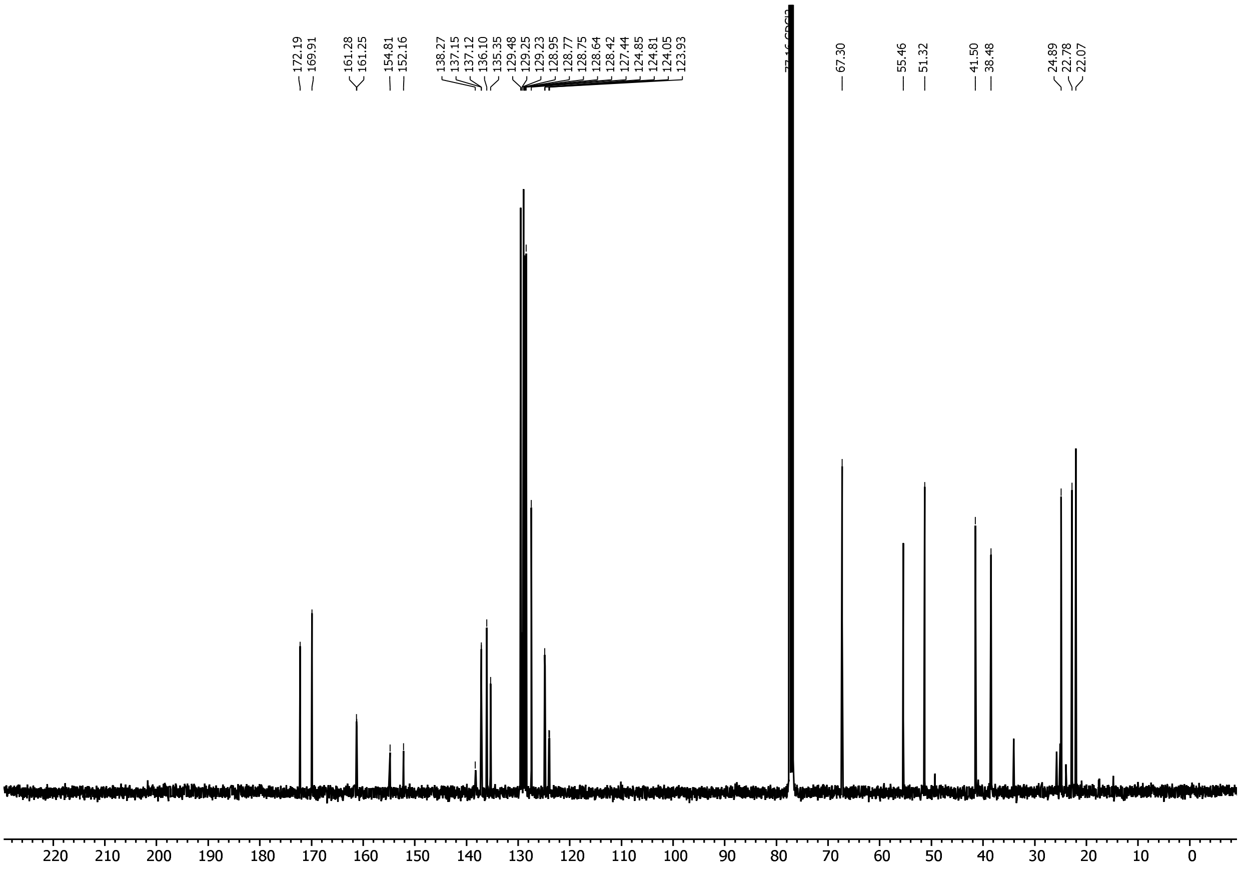

^{13}C-NMR (101 MHz, $CDCl_3$) spectrum of 4.

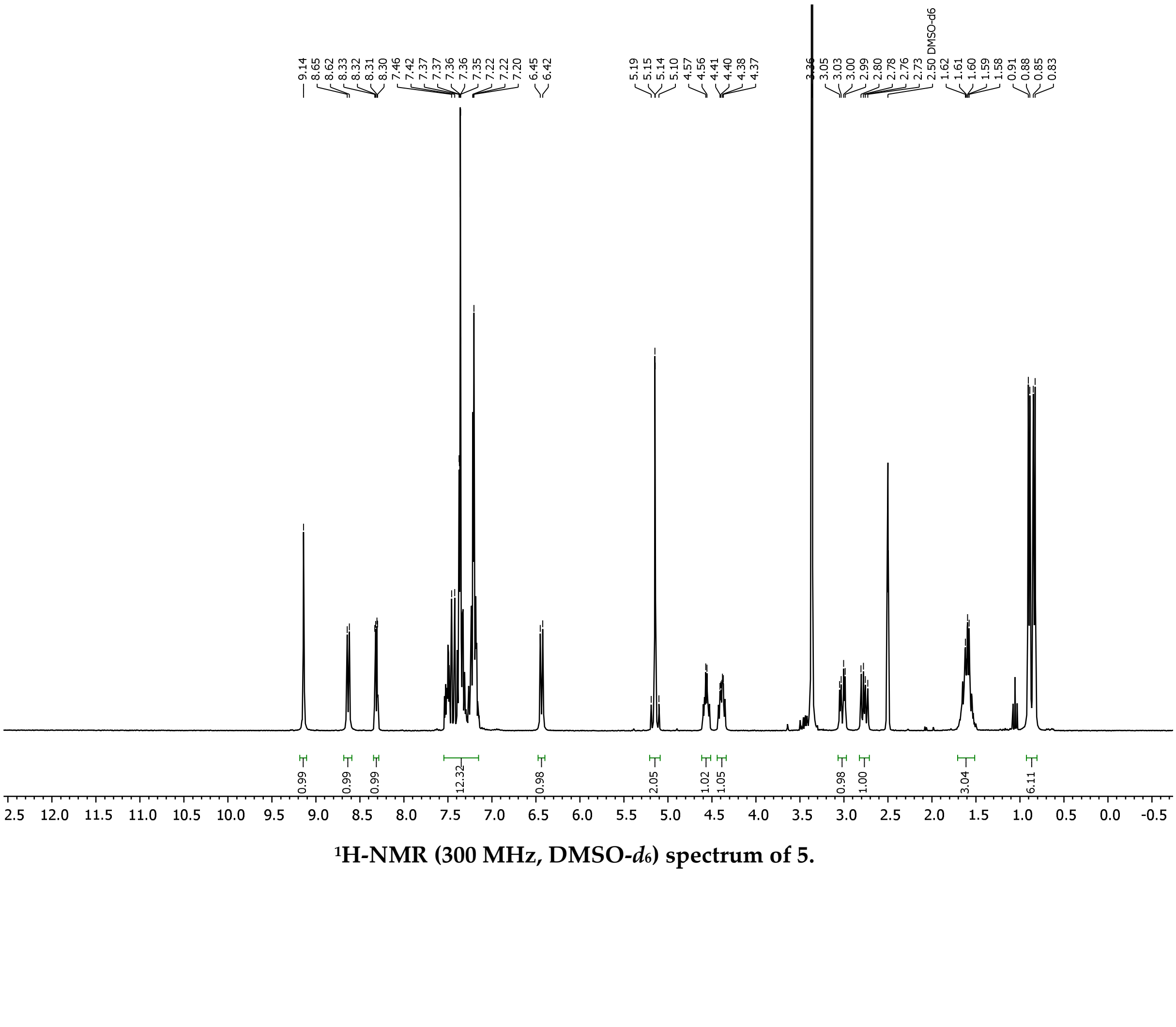

^{1}H-NMR (300 MHz, DMSO-d_6) spectrum of 5.

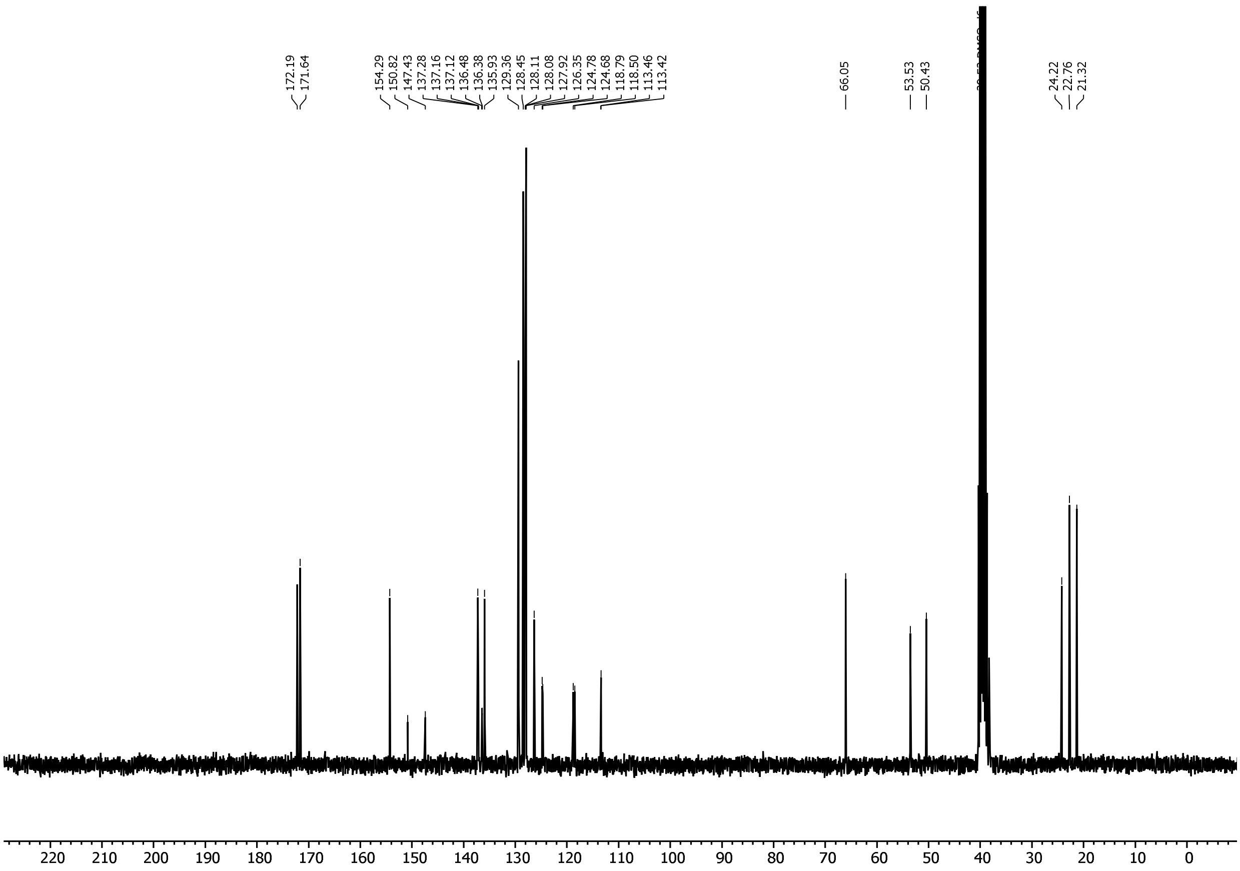

^{13}C-NMR (75 MHz, DMSO-*d*6) spectrum of 5.

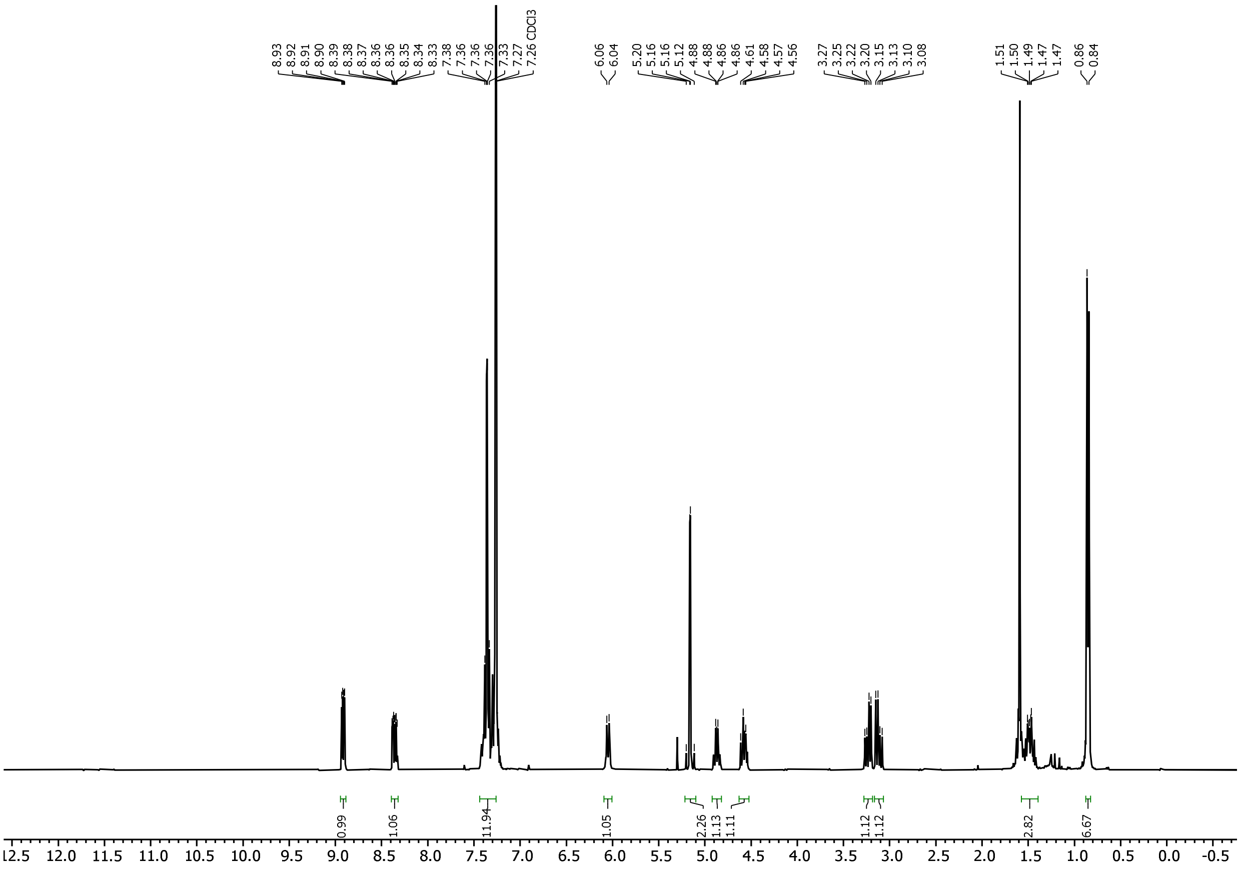

^{1}H-NMR (300 MHz, CDCl3) spectrum of 6.

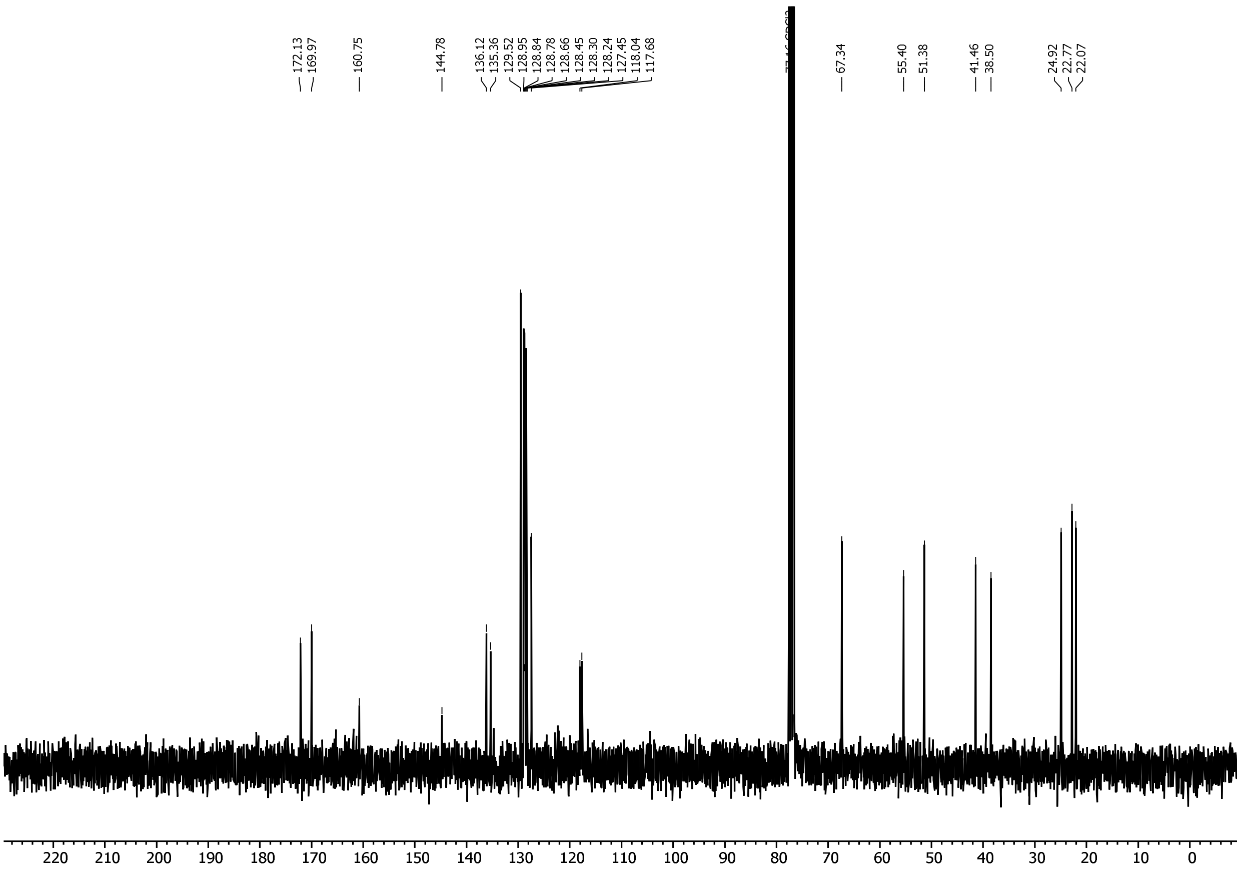

^{13}C-NMR (75 MHz, $CDCl_3$) spectrum of 6.

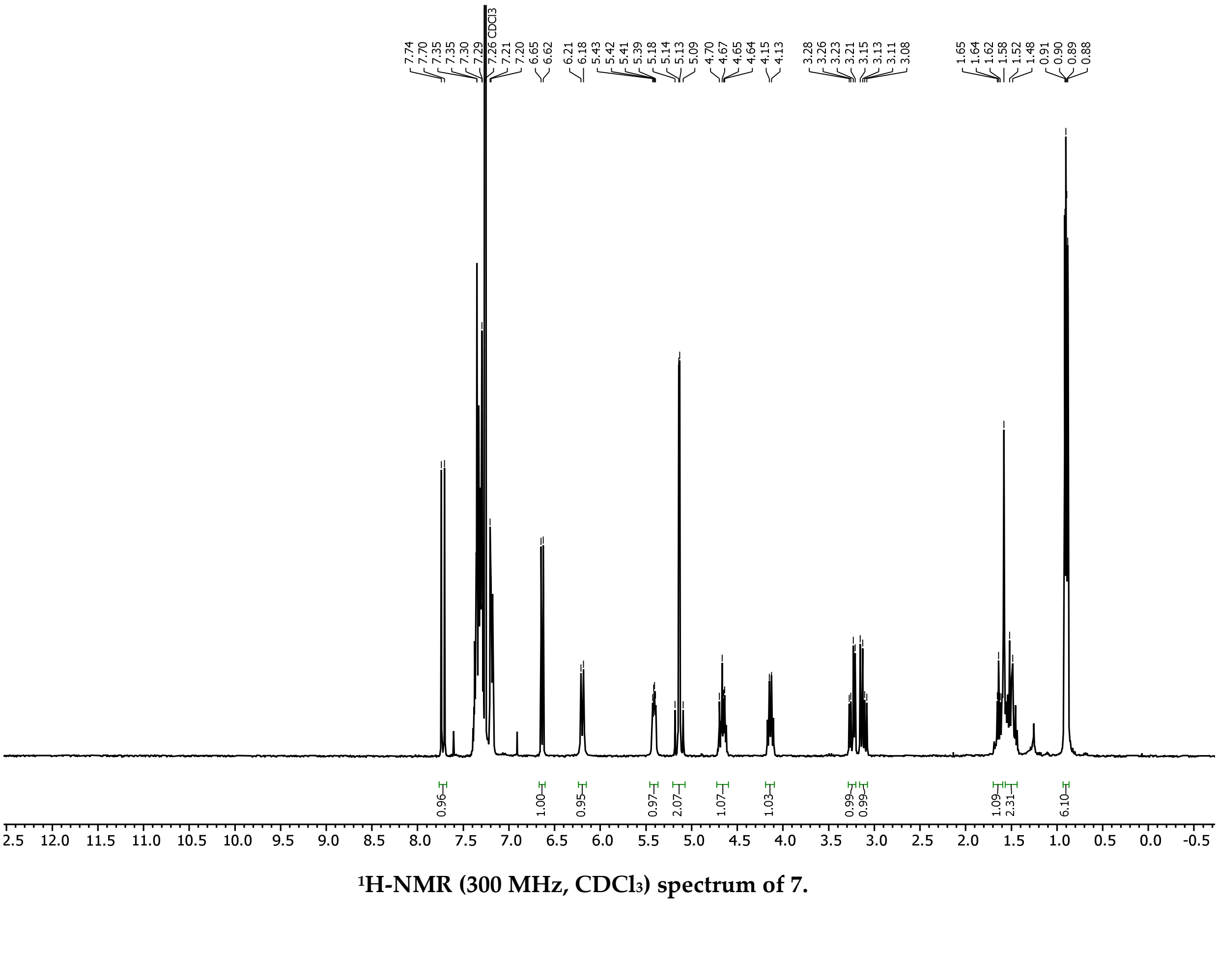

^{1}H-NMR (300 MHz, $CDCl_3$) spectrum of 7.

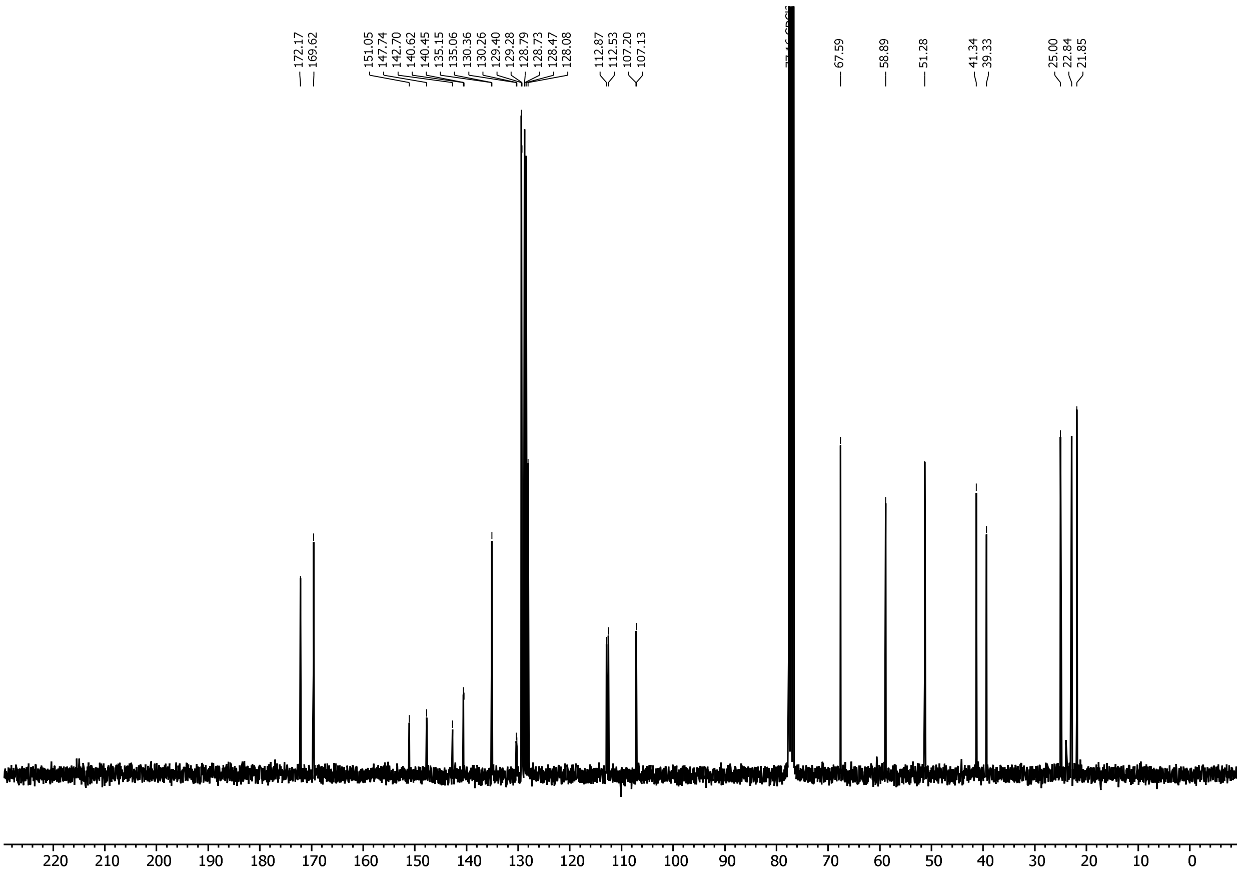

^{13}C-NMR (75 MHz, CDCl$_3$) spectrum of 7.

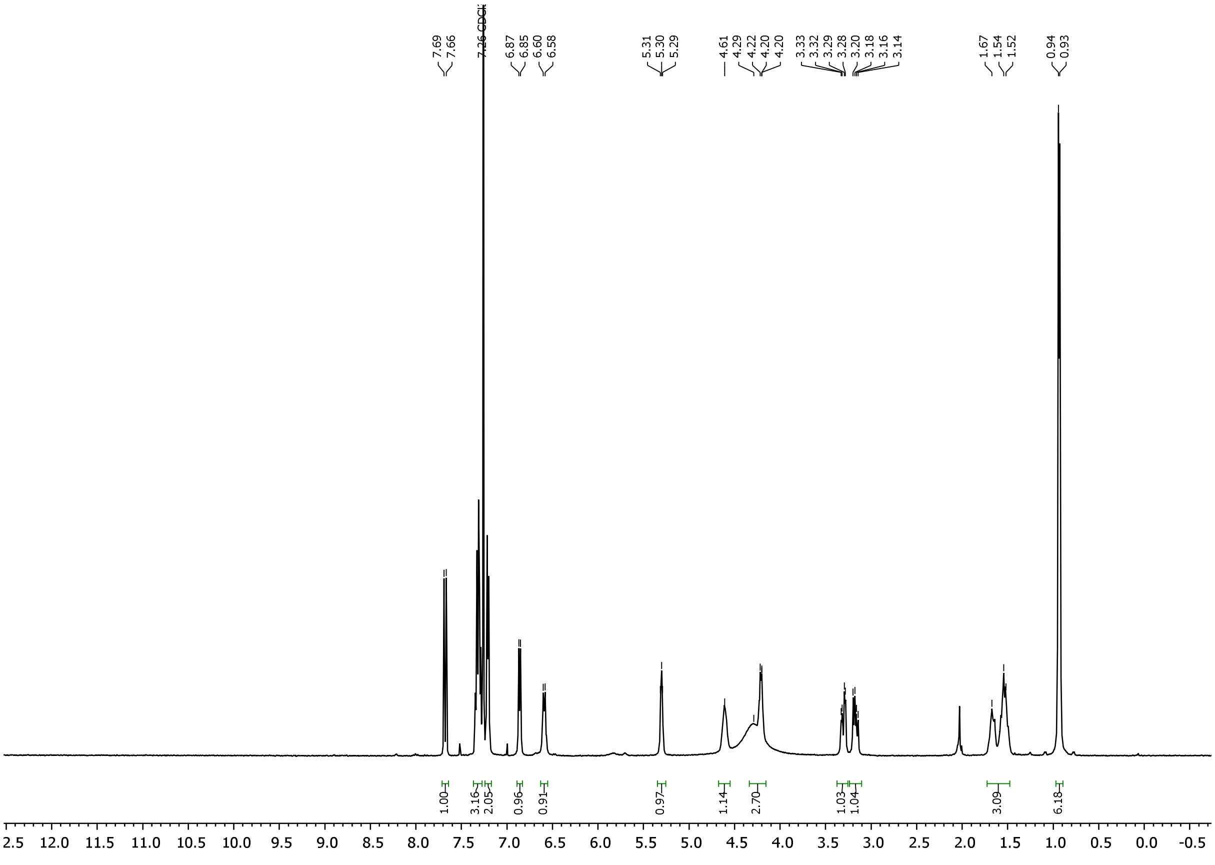

^{1}H-NMR (400 MHz, CDCl$_3$) spectrum of 8.

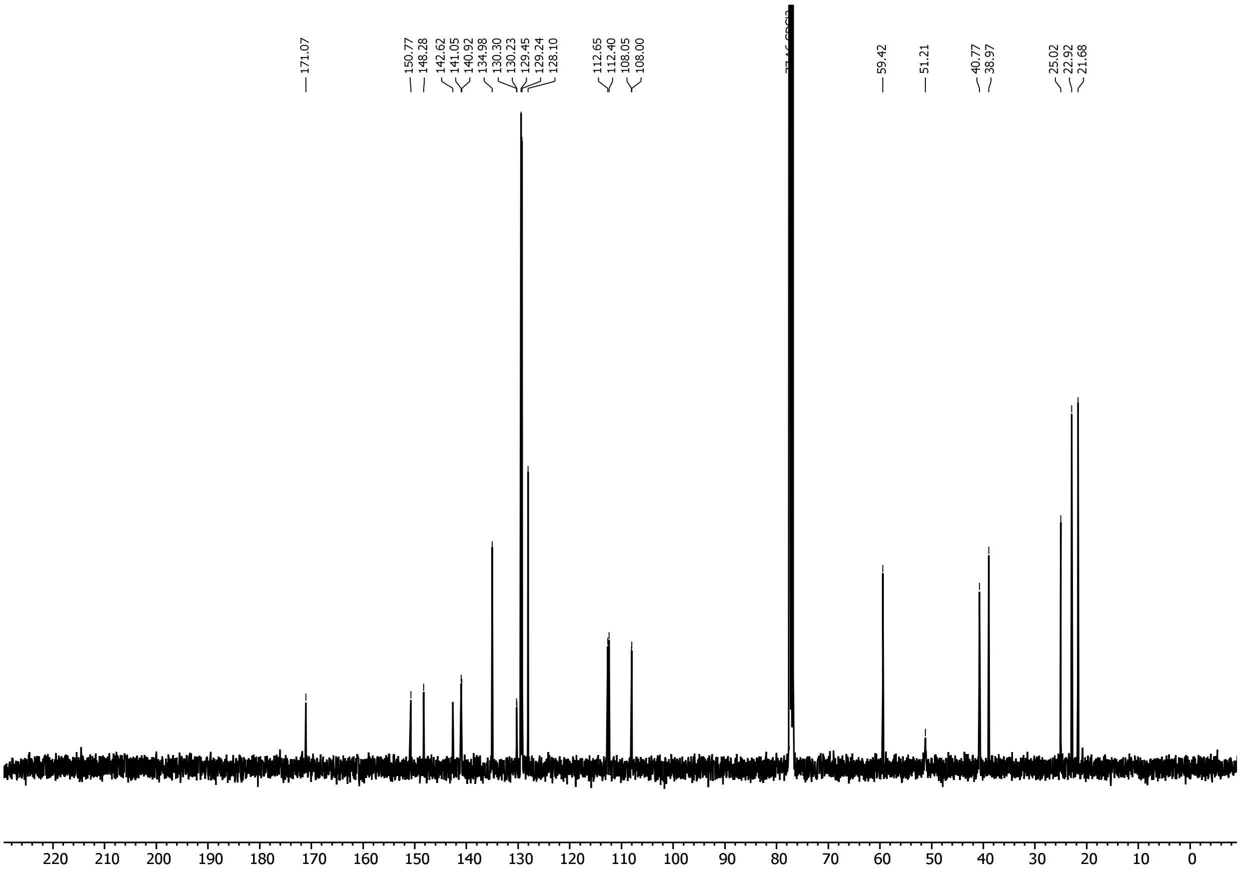

^{13}C-NMR (101 MHz, $CDCl_3$) spectrum of 8.

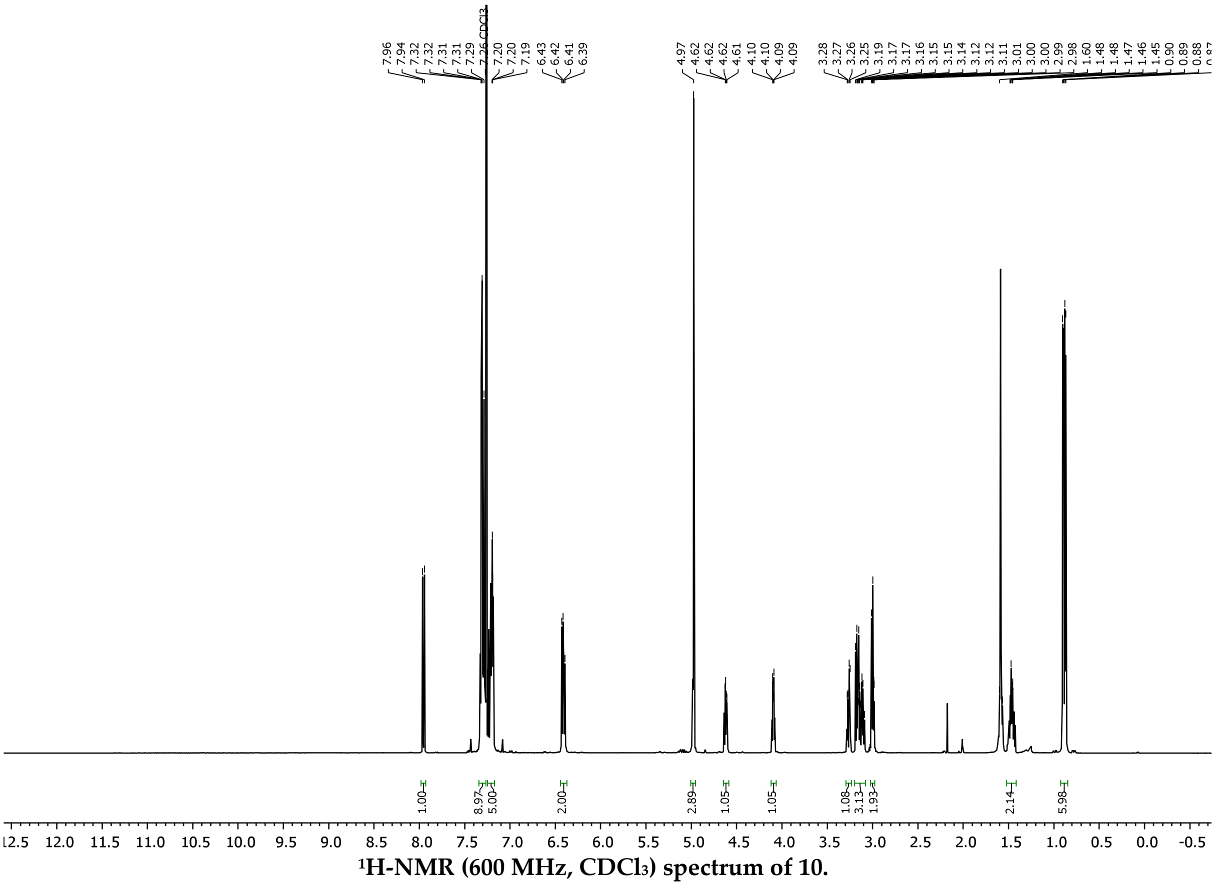

^{1}H-NMR (600 MHz, $CDCl_3$) spectrum of 10.

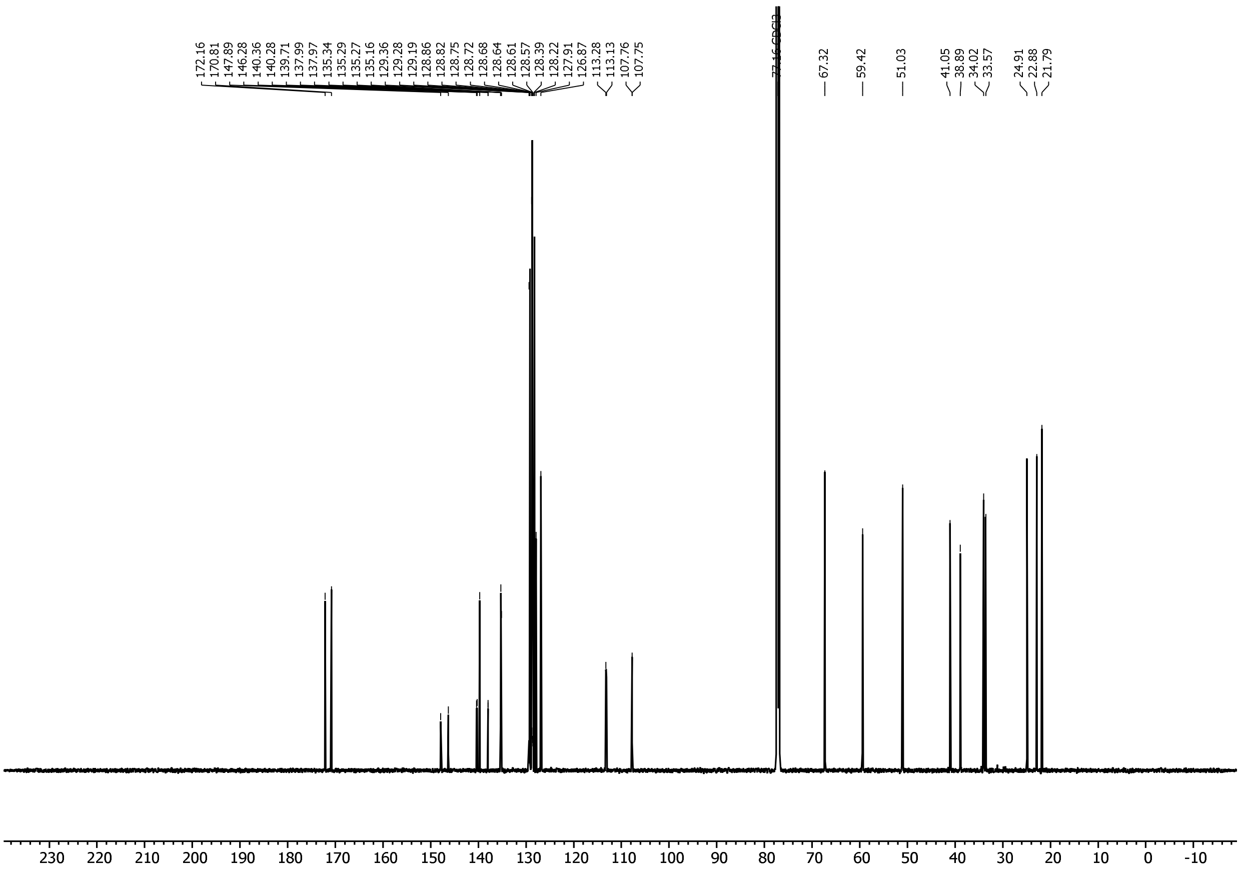

^{13}C-NMR (151 MHz, CDCl$_3$) spectrum of 10.

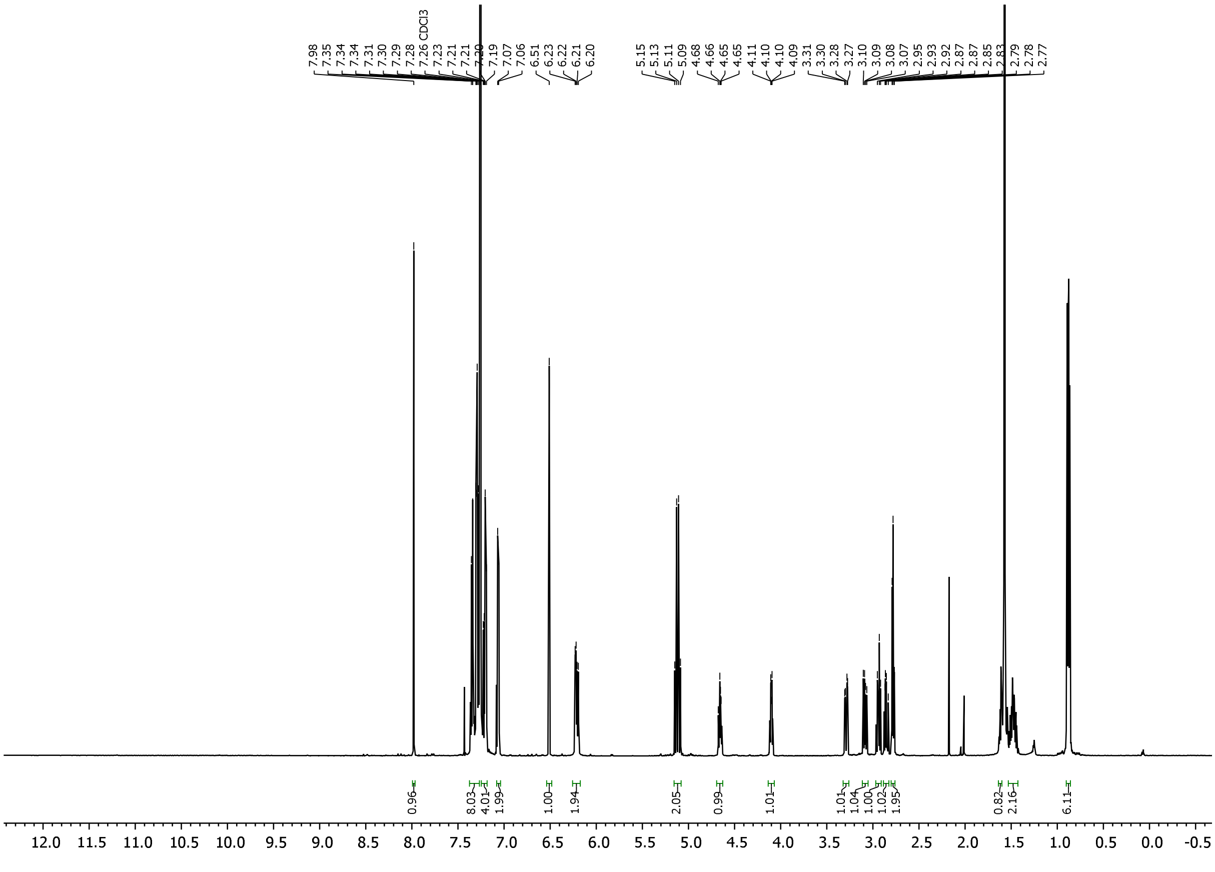

^{1}H-NMR (600 MHz, CDCl$_3$) spectrum of 11.

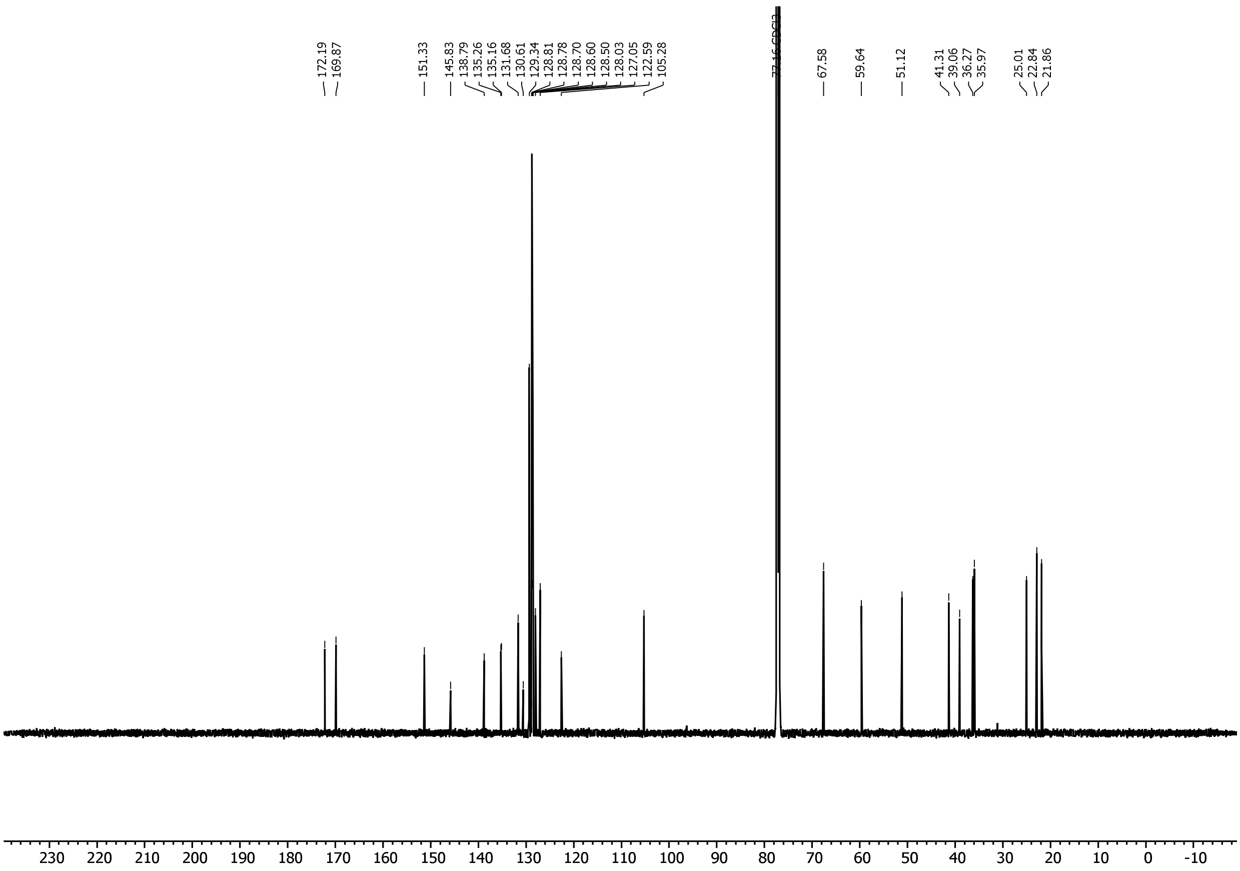

^{13}C-NMR (151 MHz, $CDCl_3$) spectrum of 11.

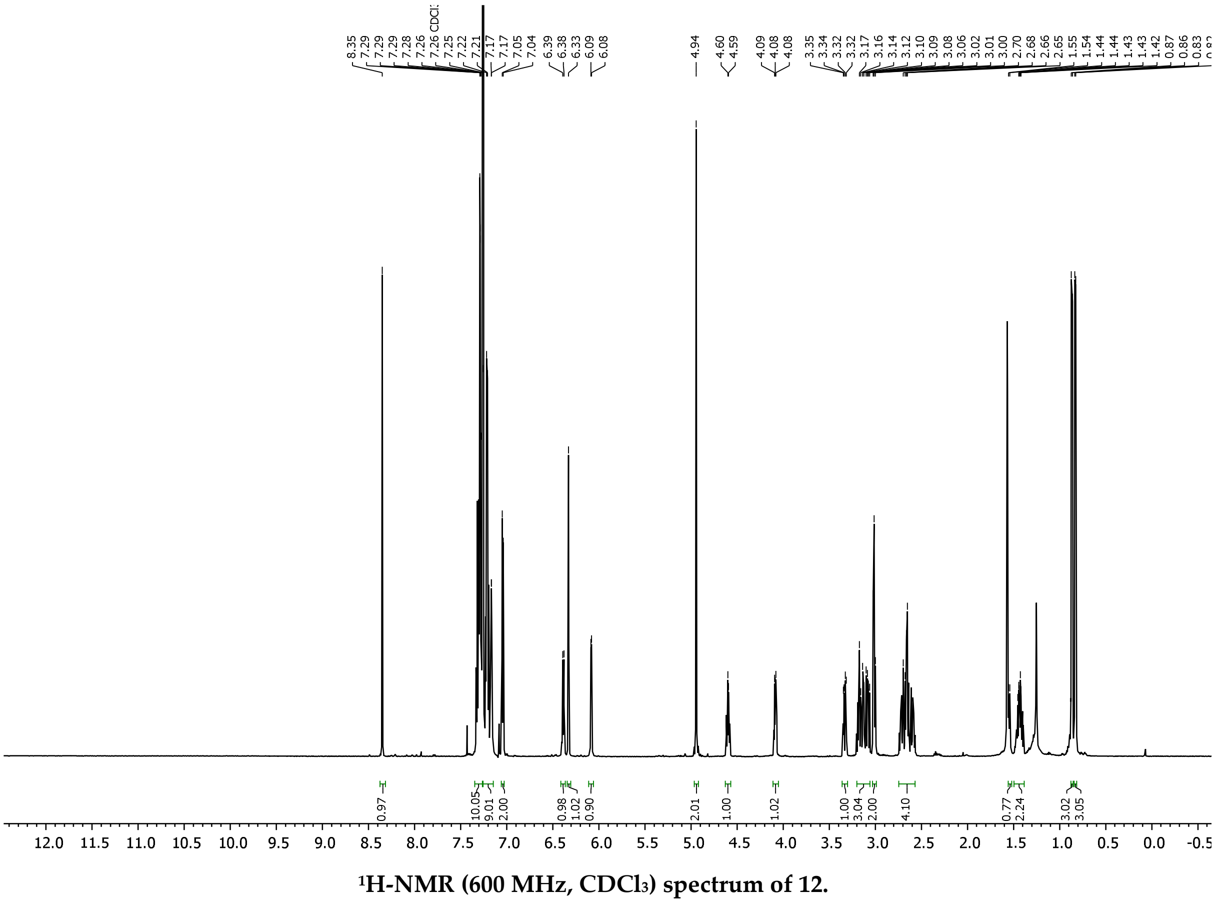

^{1}H-NMR (600 MHz, $CDCl_3$) spectrum of 12.

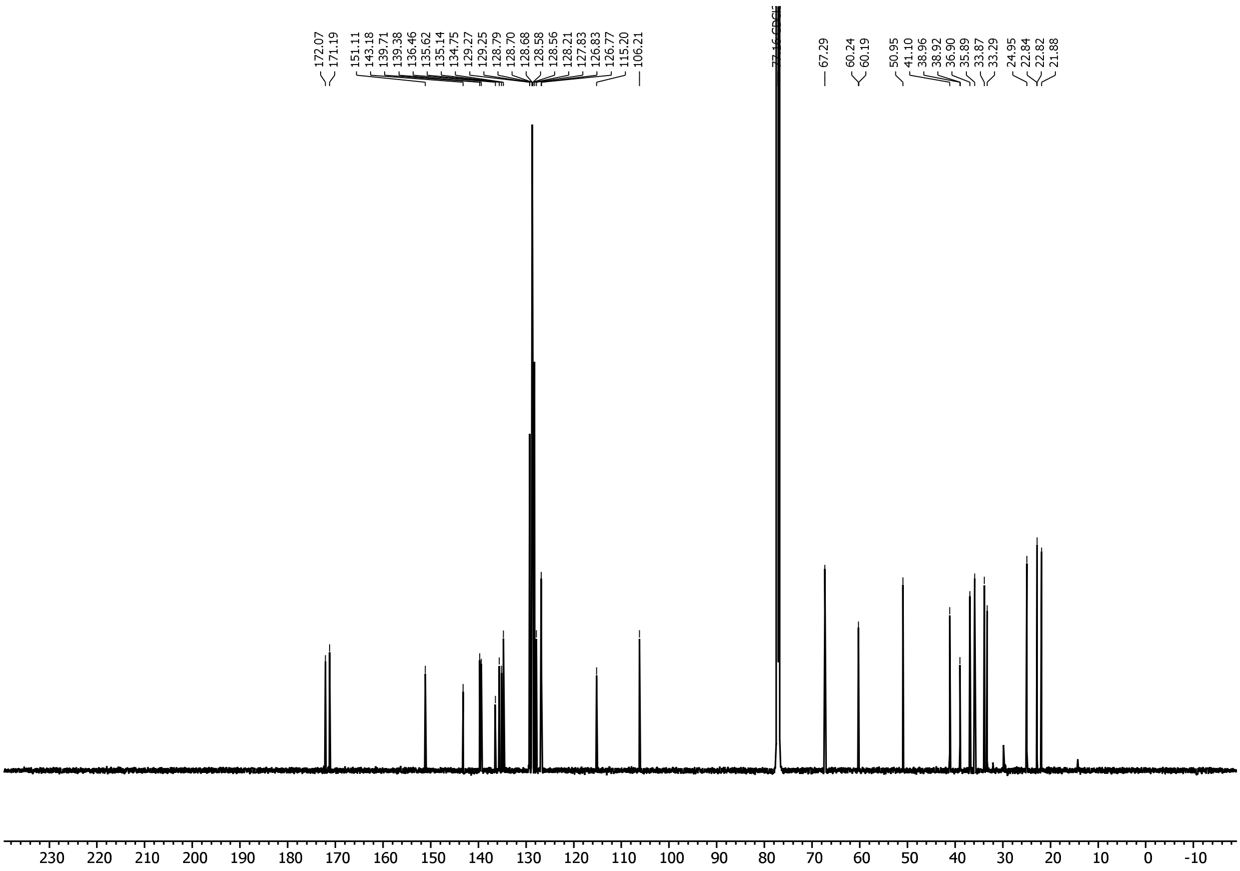

^{13}C-NMR (151 MHz, $CDCl_3$) spectrum of 12.

References

1. Gottlieb, H.E.; Kotlyar, V.; Nudelman, A.J. NMR Chemical Shifts of Common Laboratory Solvents as Trace Impurities. *Org. Chem.* **1997**, *62*, 7512–7515.
2. Lippke, G.; Thaler, H. Die spezifische Drehung des Sorbits und des Sorbit-Molybdat-Komplexes. *Starch - Stärke* **1970**, *22*, 344–351.
3. Clausen, K.; Thorsen, M.; Lawesson, S.-O.; Spatola, A.F.J. Studies on amino acids and peptides. Part 6. Methods for introducing thioamide bonds into the peptide backbone: synthesis of the four monothio analogues of leucine enkephalin. *Chem. Soc. Perkin Trans. 1* **1984**, doi: 10.1039/P19840000785.
4. Han, G.; Tamaki, M.; Hruby, V.J.J. Fast, efficient and selective deprotection of the tert-butoxycarbonyl (Boc) group using HCl/dioxane (4 M). *Pept. Res.* **2001**, *58*, 338–341.
5. de Hatten, X.; Asil, D.; Friend, R.H.; Nitschke, J.R. Aqueous Self-Assembly of an Electroluminescent Double-Helical Metallopolymer. *J. Am. Chem. Soc.* **2012**, *134*, 19170–19178.
6. Neelgund, G.M.; Budni, M.L. Studies on Charge-Transfer Complexes of 2,3-Dicyano-1,4-naphthoquinone with Some Ring and *N*-Substituted Anilines. *Monatsh. Chem.* **2004**, *135*, 1395–1407.
7. Schirmeister, T.; Kesselring, J.; Jung, S.; Schneider, T.H.; Weickert, A.; Becker, J.; Wook, L.; Bamberger, D.; Wich, P.R.; Distler, U.; Tenzer, S.; Johe, P.; Hellmich, U.A.; Engels, B. Quantum Chemical-Based Protocol for the Rational Design of Covalent Inhibitors. *J. Am. Chem. Soc.* **2016**, *138*, 8332–8335.
8. Latorre, A.; Schirmeister, T.; Kesselring, J.; Jung, S.; Johé, P.; Hellmich, U.A.; Heilos, A.; Engels, B.; Krauth-Siegel, L.R.; Dirdjaja, N.; Bou-Iserte, L.; Rodríguez, S.; González, F.V. Dipeptidyl Nitroalkenes as Potent Reversible Inhibitors of Cysteine Proteases Rhodesain and Cruzain. *ACS Med. Chem. Lett.* **2016**, *7*, 1073–1076.
9. Ludewig, S.; Kossner, M.; Schiller, M.; Baumann, K.; Schirmeister, T. Enzyme kinetics and hit validation in fluorimetric protease assays. *Curr. Top. Med. Chem.* **2010**, *10*, 368–382.
10. Cunningham, M.P.; Vickerman, K. Antigenic Analysis in the *Trypanosoma brucei* Group, Using the Agglutination Reaction. *Trans. R. Soc. Trop. Med. Hyg.* **1962**, *56*, 48–59.
11. Crouch, S.P.; Kozlowski, R.; Slater, K.J.; Fletcher, J. The Use of ATP Bioluminescence as a Measure of Cell Proliferation and Cytotoxicity. *J. Immunol. Methods* **1993**, *160*, 81–88.
12. Wagner, A.; Le T. A.; Brennich, M.; Klein, P.; Bader, N. ; Diehl, E.; Paszek, D.; Weickhmann, A.K.; Dirdjaja, N.; Krauth-Siegel, R.L.; Engels, B.; Opatz, T.; Schindelin, H.A. Inhibitor-Induced Dimerization of an Essential Oxidoreductase from African Trypanosomes. *Angew. Chem. Int. Ed.* **2019**, *58*, 3640–3644.
13. Kerr, I.D.; Lee, J.H.; Farady, C.J.; Marion, R.; Rickert, M.; Sajid, M.; Pandey, K.C.; Caffrey, C.R.; Legac, J.; Hansell, E.; McKerrow, J.H.; Craik, C.S.; Rosenthal, P.J.; Brinen, L.S. Vinyl Sulfones as Antiparasitic Agents and a Structural Basis for Drug Design. *J Biol Chem* **2009**, *284*, 25697–25703.
14. *Molecular Operating Environment (MOE)*, 2019.01, Chemical Computing Group Inc., 1010 Sherbrooke St. West, Suite #910, Montreal, QC, Canada, H3A 2R7, 2019.
15. *LeadIT/FlexX*, version 2.3.2; BioSolveIT GmbH, St. Augustin, Germany, 2018.
16. *The PyMOL Molecular Graphics System*, version 2.3.0; Schrödinger, LCC, 2019.
17. *Wavefunction, Inc.*,version 0.9, Irvine, CA, USA, 2009.
18. *Gaussian 16*, revision A.03; Gaussian, Inc.: Wallingford, CT, 2016.
19. Halgren,T.A. Merck molecular force field. I. Basis, form, scope, parameterization, and performance of MMFF94. *J. Comput. Chem.* **1996**, *17*, 490–519.
20. Stewart, J.J.P. Optimization of parameters for semiempirical methods V: Modification of NDDO approximations and application to 70 elements. *J. Mol. Model.* **2007**, *13*, 1173–1213.
21. Vosko, S.H.; Wilk, L.; Nusair, M. Accurate spin-dependent electron liquid correlation energies for local spin density calculations: a critical analysis. *Can. J. Phys.* **1980**, *58*, 1200–1211.
22. Lee, C.; Yang, W.; Parr, R.G. Development of the Colle-Salvetti correlation-energy formula into a functional of the electron density. *Phys. Rev. B* **1988**, *37*, 785–789.
23. Becke, A.D. Density-functional thermochemistry. III. The role of exact exchange. *J. Chem. Phys.* **1993**, *98*, 5648–5652.
24. Stephens, P.J.; Devlin, F.J.; Chabalowski, C.F.; Frisch, M.J. Ab Initio Calculation of Vibrational Absorption and Circular Dichroism Spectra Using Density Functional Force Fields. *J. Phys. Chem.* **1994**, *98*, 11623–11627.
25. Krishnan, R.; Binkley, J.S.; Seeger, R.; Pople, J.A. Self-consistent molecular orbital methods. XX. A basis set for correlated wave functions. *J. Chem. Phys.* **1980**, *72*, 650–654.

26. Clark, T.; Chandrasekhar, J.; Spitznagel, G.W.; Schleyer, P. v. R. Efficient diffuse function-augmented basis sets for anion calculations. III. The 3-21+G basis set for first-row elements, Li–F. *J. Comput. Chem.* **1983**, *4*, 294–301.
27. Frisch, M.J.; Pople, J.A.; Binkley, J.S . Self-consistent molecular orbital methods 25. Supplementary functions for Gaussian basis sets. *J. Chem. Phys.* **1984**, *80*, 3265–3269.
28. Ditchfield, R. Self-consistent perturbation theory of diamagnetism. *Molecular Physics* **1974**, *27*, 789–807.
29. Adamo, C.; Barone, V. Exchange functionals with improved long-range behavior and adiabatic connection methods without adjustable parameters: The *m*PW and *m*PW1PW models. *J. Chem. Phys.* **1998**, *108*, 664–675.
30. Tomasi, J.; Mennucci, B.; Cancès, E. The IEF version of the PCM solvation method: an overview of a new method addressed to study molecular solutes at the QM ab initio level. *J. Mol. Struct.: THEOCHEM* **1999**, *464*, 211–226.
31. Grimblat, N.; Zanardi, M.M.; Sarotti, A.M. Beyond DP4: an Improved Probability for the Stereochemical Assignment of Isomeric Compounds using Quantum Chemical Calculations of NMR Shifts. *J. Org. Chem.* **2015**, *80*, 12526–12534.
32. Barthels, F.; Marincola, G.; Marciniak, T.; Konhäuser, M.; Hammerschmidt, S.; Bierlmeier, J.; Distler, U.; Wich, P.R.; Tenzer, S.; Schwarzer, D.; Ziebuhr, W.; Schirmeister, T. Asymmetric Disulfanylbenzamides as Irreversible and Selective Inhibitors of Staphylococcus aureus Sortase A. *ChemMedChem* **2020**, accepted, doi.org/10.1002/cmdc.201900687.
33. Millies, B.; Hammerstein, v. F.; Gellert, A.; Hammerschmidt, S.; Barthels, F.; Göppel, U.; Immerheiser, M.; Elgner, F.; Jung, N.; Basic, M.; Kersten, C.; Kiefer, W.; Bodem, J.; Hildt, E.; Windbergs, M.; Hellmich, U.A.; Schirmeister, T. Proline-Based Allosteric Inhibitors of Zika and Dengue Virus NS2B/NS3 Proteases. *J. Med. Chem.* **2019**, *62*, 11359–11382.

© 2020 by the authors. Licensee MDPI, Basel, Switzerland. This article is an open access article distributed under the terms and conditions of the Creative Commons Attribution (CC BY) license (http://creativecommons.org/licenses/by/4.0/).
